# Safety and immunogenicity of a synthetic nanoparticle-based, T cell priming peptide vaccine against dengue in healthy adults in Switzerland: a double-blind, randomized, vehicle-controlled, phase 1 study

**DOI:** 10.1016/j.ebiom.2023.104922

**Published:** 2023-12-20

**Authors:** Alix Miauton, Régine Audran, Juliette Besson, Hélène Maby-El Hajjami, Maxime Karlen, Loane Warpelin-Decrausaz, Loredana Sene, Sylvain Schaufelberger, Vincent Faivre, Mohamed Faouzi, Mary-Anne Hartley, François Spertini, Blaise Genton

**Affiliations:** a Tropical, Travel and Vaccination Clinic, Centre for Primary Care and Public Health (Unisanté), Lausanne, Switzerland; b Division of Immunology and Allergy, Centre Hospitalier Universitaire Vaudois (CHUV), Lausanne, Switzerland; c Clinical Trial Unit, Centre Hospitalier Universitaire Vaudois (CHUV) and University of Lausanne, Lausanne, Switzerland; d Research Support Unit, Centre for Primary Care and Public Health (Unisanté), Lausanne, Switzerland; e Information Systems and Digital Transformation, Centre for Primary Care and Public Health (Unisanté), Lausanne, Switzerland; f Biostatistics Unit, Centre for Primary Care and Public Health (Unisanté), Lausanne, Switzerland

**Keywords:** Dengue vaccine, Dengue virus, T cell immunity, Nanoparticle-based vaccine

## Abstract

**Background:**

Vaccines that minimize the risk of vaccine-induced antibody-dependent enhancement and severe dengue are needed to address the global health threat posed by dengue. This study assessed the safety and immunogenicity of a gold nanoparticle (GNP)-based, multi-valent, synthetic peptide dengue vaccine candidate (PepGNP-Dengue), designed to provide protective CD8+ T cell immunity, without inducing antibodies.

**Methods:**

In this randomized, double-blind, vehicle-controlled, phase 1 trial (NCT04935801), healthy naïve individuals aged 18–45 years recruited at the Centre for primary care and public health, Lausanne, Switzerland, were randomly assigned to receive PepGNP-Dengue or comparator (GNP without peptides [vehicle-GNP]). Randomization was stratified into four groups (low dose [LD] and high dose [HD]), allocation was double-blind from participants and investigators. Two doses were administered by intradermal microneedle injection 21 days apart. Primary outcome was safety, secondary outcome immunogenicity. Analysis was by intention-to-treat for safety, intention-to-treat and per protocol for immunogenicity.

**Findings:**

26 participants were enrolled (August–September 2021) to receive PepGNP-Dengue (LD or HD, n = 10 each) or vehicle-GNP (LD or HD, n = 3 each). No vaccine-related serious adverse events occurred. Most (90%) related adverse events were mild; injection site pain and transient discoloration were most frequently reported. Injection site erythema occurred in 58% of participants. As expected, PepGNP-Dengue did not elicit anti-DENV antibodies of significance. Significant increases were observed in specific CD8+ T cells and dengue dextramer+ memory cell subsets in the LD PepGNP-Dengue but not in the HD PepGNP-Dengue or vehicle-GNP groups, specifically PepGNP-activated CD137+CD69+CD8+ T cells (day 90, +0.0318%, 95% CI: 0.0088–0.1723, *p* = 0.046), differentiated effector memory (TemRA) and central memory (Tcm) CD8+ T cells (day 35, +0.8/10^5^ CD8+, 95% CI: 0.19–5.13, *p* = 0.014 and +1.34/10^5^ CD8+, 95% CI: 0.1–7.34, *p* = 0.024, respectively).

**Interpretation:**

Results provide proof of concept that a synthetic nanoparticle-based peptide vaccine can successfully induce virus-specific CD8+ T cells. The favourable safety profile and cellular responses observed support further development of PepGNP-Dengue.

**Funding:**

Emergex Vaccines Holding Limited.

**Research in context**

**Evidence before this study**

It is estimated that almost half of the world’s population is at risk of contracting infection with dengue virus (DENV), and approximately 100 million people develop a symptomatic infection each year. Having a safe and effective vaccine against the four DENV serotypes is a global health priority. Antibody-inducing DENV vaccines carry the risk of inducing antibody-dependent enhancement (ADE) of disease, therefore other vaccine types than the existing ones are needed. Data in murine models and humans indicate that cellular immunity is essential for heterotypic DENV immunity, and potentially decreases the risk of ADE, highlighting the potential value of a CD8+ T cell-inducing vaccine. On May 1, 2023, we searched the PubMed online database for articles published using the terms “dengue”, “vaccine” and “clinical trial”, without any time or language restriction. Our search returned 140 articles. The tetravalent DENV vaccines already licensed (CYD-TDV and TAK-003) or in development (LATV-TV003/TV005, currently in phase 3, and TDEN) are mainly live attenuated. A purified inactivated vaccine (DPIV) has been studied in a phase 2 trial; a DNA vaccine (TVDV) and a protein subunit vaccine (V180) have been tested in phase 1 trials. These vaccines are all expected to induce humoral immunity.

**Added value of this study**

The particularity of our study is to assess the safety and immunogenicity in humans of a DENV vaccine specifically designed to elicit a cytotoxic CD8+ T cell immune response, without inducing humoral immunity. The nanoparticle-based structure of PepGNP-Dengue is also innovative. Overall, the investigational vaccine appeared to be safe. Similar local reactogenicity, at least partly related to the vehicle, was recently described in another study with intradermal gold nanoparticles. PepGNP-Dengue did not induce significant humoral response against all four DENV serotypes–as expected–and elicited (or boosted) dengue-specific cellular immunity, primarily in participants in the low dose group.

**Implications of all the available evidence**

These findings support further investigation of this innovative dengue vaccine, which in addition to being a T cell priming vaccine, may also potentially act to prevent ADE of disease. This is also potentially a promising approach for vaccine development for other diseases with likelihood of antibody-dependent disease enhancement and for which CD8+ T cell-mediated immunity is important.

## Introduction

The four dengue viruses (DENV 1-4) transmitted by the *Aedes* mosquito cause approximately 390 million infections per year, of which 96 million are symptomatic,^1^ and an estimated 500,000 require hospitalization.^2^ The incidence of dengue disease has risen dramatically in recent years with increasingly explosive outbreaks, partly due to urbanization and the geographic expansion of the vector facilitated by climate change.^3^^,^^4^

With dengue declared as one of the top ten threats to global health,^5^ having a safe and effective vaccine is one of the cornerstones identified in the WHO Global Strategy for Dengue Prevention and Control.^6^ The ideal vaccine should provide high protection against all four serotypes, be given as a single dose, and induce long-term immunity, with no serious adverse event (SAE).^6^ No vaccine currently fulfils these criteria. One of the licensed dengue vaccines, CYD-TDV (Dengvaxia®; Sanofi Pasteur) has been associated with an increased risk of severe dengue in individuals who were seronegative before vaccination, presumably due to antibody-dependent enhancement (ADE).^7^^,^^8^ Similar to a first natural infection, sub-protective levels (immediately after vaccination or with waning immunity) of vaccine-induced cross-reactive antibodies do not neutralize heterotypic DENV but rather facilitate its cellular invasion, increasing the risk of severe dengue. Vaccination with CYD-TDV is therefore recommended only in individuals with evidence of previous dengue infection.^9^ Although a second vaccine (TAK-003, QDENGA®; Takeda) has recently been approved for use in individuals without the need for pre-vaccination testing, there are concerns that safety data are insufficient and that the possibility of ADE cannot be ruled out.^10^^,^^11^

Studies in murine models suggest that CD8+ T cells are essential for protection against heterotopic DENV infections and potentially reduce the risk of ADE.^12^^,^^13^ Data in humans also indicate that DENV-specific T cells are associated with protective immunity.^14^ It has been suggested that CYD-TDV fails to elicit a robust T cell response, thereby potentiating ADE.^15^ Inducing effective and long-lasting T cell immunity is, therefore, an important requirement for vaccination against DENV.^16^

PepGNP-Dengue is a candidate vaccine that has been designed to elicit a specific cytotoxic CD8+ T cell response, without inducing a humoral one. It is composed of synthetic T cell-selective multi-valent DENV peptides carried on ultrasmall gold nanoparticles (GNPs), chosen for their immunogenic properties,^17^ layered with carbohydrates mimicking a bacterial pattern (‘self-adjuvanting’ vaccine) and designed to mimic the process of antigen presentation during natural infection.^18^ Their intradermal administration aims to partially mimic a natural infection from a mosquito bite, target skin antigen-presenting cells (APCs), and induce a protective local CD8+ response.

The safety and immunogenicity of PepGNP-Dengue have been demonstrated in preclinical *in vitro* and *in vivo* studies. In a clinical trial setting, intradermal administration of peptide-conjugated GNPs has been reported to be safe and well tolerated.^19^ Here, we report the results of naNO-DENGUE, a phase 1 randomized, double-blind clinical trial, whose primary objective was to evaluate the safety, tolerability, and reactogenicity of two different doses of PepGNP-Dengue administered for the first time to healthy participants. A secondary objective was to assess the immunogenicity of PepGNP-Dengue, by assessing the evidence of a CD8+ T cell-mediated immune response as a surrogate of protection against severe dengue, and evaluating antibody responses (absence of) against the four DENV serotypes.

## Methods

### Study design and participants

naNO-DENGUE was a single-centre, vehicle-controlled, randomized, double-blind, dose-finding phase 1 trial to assess the safety and immunogenicity of PepGNP-Dengue (low dose [LD] and high dose [HD]), administered in two doses 21 days apart.

The study took place in a non-dengue endemic setting, at the Centre for primary care and public health and Clinical Trial Unit of the Centre Hospitalier Universitaire Vaudois (CHUV), Lausanne, Switzerland. Participants were recruited through advertisements posted in communication networks of local academic and hospital institutions. Eligible participants were healthy men and women aged 18–45 years residing in Switzerland, with normal clinical and laboratory findings at the screening visit. Exclusion criteria included: history of flavivirus infection or previous vaccination against Japanese encephalitis, yellow fever, or dengue; past residence or planned trip during the trial to a highly endemic region for flavivirus (excepting tick-borne encephalitis [TBE] and West Nile virus); hypersensitivity to gold; pregnant or lactating women, or those with childbearing potential. Since the study took place at a time of high prevalence of SARS-CoV-2 infections, if there was a clinical suspicion of COVID-19 on the days of vaccination, the participant was required to undergo a SARS-CoV-2 PCR or rapid antigen test, with vaccination delayed until a negative result was confirmed. The comprehensive list of inclusion and exclusion criteria is detailed in Appendix A and the protocol is available online.

### Ethics

This study was approved by the local ethics committee (Commission cantonale (Vaud) d'éthique de la recherche sur l'être humain, 2020-02258) and by Swiss regulatory authorities (Swissmedic, 2021DR1042). All participants provided written informed consent. This study is registered at ClinicalTrials.gov, NCT04935801.

### Randomization and masking

Eligible participants were enrolled and randomly assigned to receive PepGNP-Dengue or vehicle-GNP on the day of the first vaccination (day 0). Vehicle-GNP was chosen as a control to better isolate the effect of the DENV peptides from that of the gold nanoparticles. Four computer-generated stratified randomization lists (LD and HD, pioneers and followers) were created by CHUV pharmacists. The protocol followed a risk-minimizing dose-escalation strategy, where enrolment and randomization were carried out sequentially, conditional on favourable interim safety analyses. Since this is a first-in-human trial, only one participant was vaccinated per day for the first three ‘pioneer’ participants in each dosage group (randomization by block of three, 2:1, vera, comparator). After a first safety review of the three LD ‘pioneers’ performed by a Pharmacovigilance clinical research organisation (CRO), ten LD ‘follower’ participants were randomized by block of five (8:2, vera, comparator) and vaccinated. An independent data and safety monitoring committee (DSMC), comprising one vaccinologist, two clinical pharmacologists, and one statistician, performed a planned safety analysis prior to dose escalation, reviewing the safety data of the thirteen LD participants before proceeding to LD group second vaccinations and HD group first vaccinations. Safety data of the three HD ‘pioneers’ were reviewed by the Pharmacovigilance CRO before randomizing (block of 5, 8:2, vera, comparator) and vaccinating the ten HD ‘followers’. In addition, specific safety holding rules were defined to trigger immediate suspension of the trial (where restart would have been subject to DSMC review and Ethics Committee approval). These rules are detailed in Appendix B.

Only pharmacists responsible for vaccine product reconstitution (but not involved in collection or production of safety and immunological data) had access to the allocation list. The investigators, sponsor, and participants were masked to group assignment. PepGNP-Dengue and vehicle-GNP syringes were identical in volume and appearance. The clinical and immunological teams remained blinded until the respective databases were frozen.

### Procedures

Dengue peptides in PepGNP-Dengue were selected using an immunoproteomics approach, validated in *in vitro* studies.^20^ Briefly, a human leukocyte antigen (HLA)-typed human cell line was infected with DENV. Peptides expressed on major histocompatibility complex (MHC) class I of infected cells (‘ligandome’, i.e., targets of the CD8+ T cells during natural dengue infection) were extracted and identified using mass spectrometry analysis. Nine selected HLA-class I dengue peptides (Supplementary Table S1) were synthesized with a proteasome cleavage sequence (AAY) in N-term and a linker (3-mercapto propionyl) to facilitate binding to a GNP. Gold core approximately 1.6 nm in diameter was passivated with equimolar amounts of N-acetyl glucosamine (2-[2-Mercaptoethoxy]ethyl 2-acetamido-2-deoxy-β-d-glucopyranoside) and alpha-galactose (2-Mercaptoethyl α-d-galactopyranoside). The total size of the vaccine construct was approximately 5–6 nm. For the top dose of 7.5 nmol total peptide (i.e., all nine peptides combined), 44.5 μg of gold, 19.4 μg of GlcNAc, and 15 μg of α-Gal were also administered.

DENV peptides and GNPs were manufactured according to current good manufacturing practices by CSBio (USA) and Ardena-Oss (Netherlands), respectively. The final drug product (PepGNP-Dengue and vehicle-GNP) was lyophilized and packed by Symbiosis Pharmaceutical Services Limited (UK) and stored at −20 °C. CHUV pharmacists reconstituted the final freeze-dried powder product with water for injection for administration.

Two doses (0.05 ml each) were administered intradermally 21 days apart in the deltoid region of the upper arm using the Nanopass MicronJet600 microneedle. A second injection was given to promote the generation of CD8+ T cells with innate and help-independent recall capacities, cytotoxic T effector programme and increased tissue invasiveness (i.e., tissue-resident T memory [TRM] cells).^21^ The intradermal route was preferred due to the low dosage required and to best mimic immune cell recruitment during natural infection by a mosquito bite.^22^^,^^23^ The LD formulation contained GNP alone with a gold content of 14.8 μg (LD vehicle-GNP), or with 2.5 nmol of DENV ligandome peptides (LD PepGNP-Dengue); the HD formulation contained GNP alone with a gold content of 44.5 μg (HD vehicle-GNP) or with 7.5 nmol of DENV ligandome peptides (HD PepGNP-Dengue). Dosages were selected based on preclinical testing, with the selected peptide doses found to be within the bounds of, and approximately in the centre of, the efficacy range tested in HLA transgenic animals. Participants were followed at regular intervals for up to six months after the first injection (days 1, 7, and 14 after each injection and on days 60, 90, and 180). Visits on days 1, 22, and 60 were performed remotely (by phone), while others consisted of on-site interviews with blood sample collection.

Reactogenicity and safety were assessed 1 h post-injection and at the above-mentioned time points. Solicited local (pain, tenderness, erythema, and swelling) and systemic reactions (fever, headache, nausea, diarrhoea, fatigue, myalgia) occurring up to 7 and 14 days after each injection, respectively, were recorded by participants in diary cards. Participants were given a ruler to measure the size of local erythema/swelling and a thermometer to record their temperature daily. The investigators assessed unsolicited adverse events (AEs) and adverse events of special interest (AESIs) (Appendix C) in terms of timing, duration, severity, seriousness, and relatedness with the study product during the entire study period. Safety laboratory analyses, including haematology, kidney, and liver function tests were performed at screening and on days 7 and 14 after each injection. The investigators assessed the severity of AEs and their relationship with the study product according to predefined scales and guidelines.

Immunological analyses were performed on days 0, 21, 35, 90 and 180. Humoral immune responses against dengue virus particle DENV1-4, or NS1 and tick-borne encephalitis virus (TBE) were measured using commercial ELISA kits (anti-dengue virus types 1-4 IgG, # EI 266a-9601-1 G; anti-dengue virus NS1 type 1-4 IgG, # EI 266a-9601-2 G; Euro Immun, Lübeck, DE; FSME/TBE ELISA IgG/IgM, # EC117.00, Virotech Diagnostics, Rüsselheim, DE; Appendix D1a). A rapid diagnostic test (RDT, Bioline Dengue Duo (NS1 Ag + Ab combo), Standard Diagnostics, KR) was performed at screening to exclude dengue-exposed individuals, and at days 14 and 35 (two weeks after each injection) to assess the DENV-specific antibody response. The non-vaccine-specific response was evaluated using a custom tetanus toxoid (TT) ELISA (detailed in Appendix D1b).

Circulating CD8+ T cell responses induced by PepGNP-Dengue were determined by cytometry: i) by measurement of activation-induced markers (AIM) at baseline and days 21, 35, 90, and 180 and ii) using HLA class I dextramers at baseline, day 35 and 180. Class I HLA typing was performed on whole blood by RT-PCR, using the kit LinkSeq HLA ABCDRDQDP SABR (Linkage Bioscience).

For AIM, the frequency of CD8+ cells expressing the activation co-markers CD107a+CD25+ and/or CD137+CD69+ was measured upon stimulation of peripheral blood mononuclear cells (PBMCs) with DENV peptides or PepGNP-Dengue, adapted from published methods.^24^^,^^25^ PBMC, 1 to 2 million cells per well in duplicate, were stimulated for 24 h with 2 μg/ml of a pool of 11 dengue peptides (Appendix D1c, Supplementary Table S1), 0.6 μM of PepGNP-Dengue, an equivalent concentration of vehicle-GNP (3.6 μg/ml of gold), 5 μg/ml of staphylococcal enterotoxin B (SEB, # S4881, Sigma) or unstimulated (X-vivo-15 0.3% DMSO), as controls, in presence of anti-CD107a-PE. After washing, cells were stained with markers described in Supplementary Table S3 in the presence of 50 μg/ml of human Immunoglobulin G (IgG) (Privigen®, CSL Behring, CH) for 20 min at 4 °C. After washing, cells were resuspended in FACS Lysing solution (BD, # 349202) (10 min, RT) and then resuspended in phosphate-buffered saline (PBS) 0.1% bovine serum albumin (BSA) 2 mM EDTA and stored at 4 °C before acquisition. The acquisition was performed on an Attune nxt or an LSR Fortessa and analysis performed on Flow-Jo v10.8.1. Gating strategy is shown in Supplementary Figure S1.

For dextramer, we used eight dengue (four HLA-A∗0201, three B∗0702, and one A∗0301) and control dextramers (MHC Dextramer®, Immudex, DK) described in Supplementary Table S2. The method was adapted from Dolton.^26^ PBMC taken at various timepoints were distributed in 96-well V bottom plates, 3 million cells per well, one to three wells per timepoint, depending on the HLA type of the individual. For each volunteer, a pool of PBMC from different timepoints was used for positive and negative control wells. First, cells were incubated for 30 min at 37 °C with 25 μL of dasatinib 50 nM (Sigma, # CDS023389, CAS 302962-49-8). Then, 55 μL of PBS-0.5% BSA containing 1–6 dextramers were added and cells stained for 10 min at RT. Pools of dextramers were buffered with PBS 10× and completed with 1.78 μM biotin (Sigma, #B4639, CAS 58-85-5). Finally, cells were stained with 20 μL of 5× concentrated markers described in Supplementary Table S3, in the presence of 50 μg/ml human IgG (Privigen®, CSL Behring, CH) for 20 min at 4 °C. After washings, cells were resuspended in FACS Lysing buffer (BD) (5 min, RT), then resuspended in PBS 0.1% BSA 2 mM EDTA. The acquisition was performed on an LSR Fortessa and analysis performed on Flow-Jo v10.8.1. Gating strategy is shown in Supplementary Figure S2.

### Outcomes

Primary outcomes were frequency and severity of solicited local and systemic AEs 7 and 14 days after each vaccination, respectively; occurrence of unsolicited AEs, AESIs, and SAEs during the entire trial period; and change from baseline for safety laboratory measures.

Secondary outcomes were frequency of CD8+ T cells specific to PepGNP-Dengue peptides at baseline, day 21, 35, 90, and 180 after the first vaccination, and antibody titre to all four DENV serotypes.

Exploratory outcomes were HLA-related and in-depth characterisation of the dengue-specific CD8+ T cell response.

### Statistics

Since this is a first-in-human study, the number of participants needed to be limited. The sample size was not based on statistical hypothesis testing but was computed to detect AEs with a high incidence rate. Having ten participants per group exposed to PepGNP-Dengue allowed an 80% power of detecting an AE with a true incidence of 7.5% across all exposed participants (LD and HD combined) or 20% within a single dose group. Analysis was by intention-to-treat (ITT) for safety and both ITT and per protocol (PP) for immunogenicity, based on actual results with no missing data imputed.

AEs are described with absolute and relative frequencies (%) according to the group to which participants were randomized. Mean, standard deviation (SD), minimum, maximum, median, and quartiles were used for continuous safety laboratory variables. Safety analyses are descriptive only; because of the small sample size, no formal statistical hypotheses were tested.

For AIM analyses, results are expressed as relative frequencies (%) of CD8+ T cells co-expressing CD107a+CD25+ and CD137+CD69+ or positive for at least one of the 2 co-marker combinations. Results in unstimulated conditions were subtracted from stimulated results. Change from baseline was defined as the delta post- minus pre-vaccination response. The cut-off for positive response was defined as the mean +2 SD of the DENV-specific AIM+CD8+ T cell response in all volunteers at day 0. A responder was defined as a participant with a positive delta AIM+CD8+ T cell response and a response above the cut-off.

For dextramer analyses, results are expressed as the number of CD8+ T cells DENV dextramer positive (D-dextr+)/10^5^ CD8+ T cells. Change from baseline was defined as the ratio of post- to pre-vaccination response. The cut-off for positive response was defined as the mean +2 SD of the D-dext+ CD8+ response in all volunteers at day 0 and as the mean ratio +2 SD in the vehicle-GNP group at day 35. A responder was defined as a participant with a frequency of specific CD8+ and a ratio above their respective cut-off.

For immunological analyses, median and 95% confidence intervals (CI) are presented. Non-parametric intra-group comparisons were conducted using the Friedman test, while inter-group comparisons utilised the Kruskal–Wallis test. Dunn’s multiple comparisons test was employed for comparison with day 0 or at each timepoint. The Fisher’s exact test was used to compare the number of responders. A significance level of 0.05 was set. LD and HD vehicle-GNP participants were pooled in the group vehicle-GNP (n = 6).

Demographic, clinical, and safety data were collected and managed using REDCap electronic data capture tools hosted at Unisanté, then analysed with STATA 16 and Excel. Immunogenicity data were analysed with Excel and GraphPrism (version 9.1.0).

### Role of funders

Emergex Vaccines Holding Limited contributed to the study design, data interpretation, and manuscript revision. All study data were available to authors upon request. The principal investigator made the final decision to submit the findings for publication.

## Results

### Subjects

**Fig. 1:**
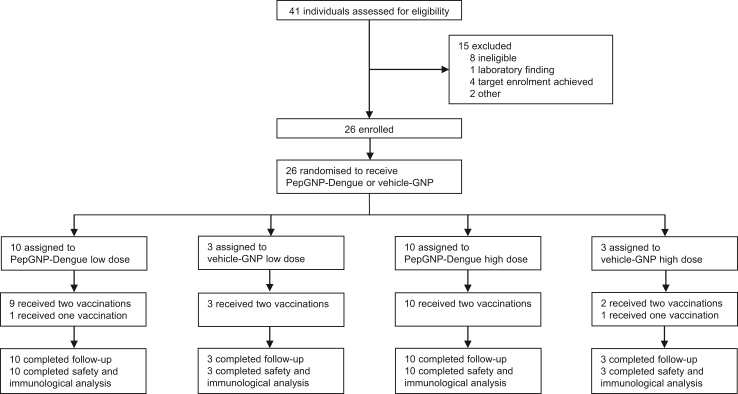
**naNO-DENGUE trial profile.** Participants were enrolled according to a dose-escalation protocol and randomly assigned within each dose group to receive two doses of PepGNP-Dengue (gold nanoparticles and dengue peptides) or vehicle-GNP (gold nanoparticles only). Two participants did not receive the second vaccination: one because of consent withdrawal (PepGNP-Dengue low dose group) and one because of grade 3 erythema at the injection site (vehicle-GNP high dose group). All participants completed 180 days of follow-up. GNP = gold nanoparticles.

From 41 individuals screened, 26 participants were enrolled from the 2nd August to the 9th September 2021, receiving two doses of either PepGNP-Dengue LD (n = 10), vehicle-GNP LD (n = 3), PepGNP-Dengue HD (n = 10) or vehicle-GNP HD (n = 3) 21 days apart (Fig. 1). Our aim was to include a balanced number of men and women according to their self-reported sex. Close to one-half (12 [46%]) of the study population were female. The majority of participants (23 [88%]) were Caucasian, and the mean age was 29 (range 20–43) years (Table 1). Two participants did not receive the second vaccination: one LD PepGNP-Dengue-recipient for personal reasons, and one HD vehicle-GNP-recipient due to grade 3 local erythema after the first injection. Both remained included in the study. All participants completed the 180-day study follow-up, with only one missed telephone visit the day after the second vaccination for the two participants not vaccinated.

**Table 1:** Summary of demographic and baseline characteristics by treatment group.

	LD PepGNP-Dengue (n = 10)	LD vehicle-GNP (n = 3)	HD PepGNP-Dengue (n = 10)	HD vehicle-GNP (n = 3)	All participants (n = 26)
Sex					
Female	4 (40%)	2 (67%)	5 (50%)	1 (33%)	12 (46%)
Male	6 (60%)	1 (33%)	5 (50%)	2 (67%)	14 (54%)
Ethnicity					
Other	1 (10%)	0 (0%)	1 (10%)	1 (33%)	3 (12%)
Caucasian	9 (90%)	3 (100%)	9 (90%)	2 (67%)	23 (88%)
Age, years					
Mean	30.9 (5.8)	25.7 (1.5)	28.5 (6.4)	29.7 (6.1)	29.2 (5.7)
Range	23–40	24–27	20–43	23–35	20–43
BMI, kg/m^2^					
Mean	25.3 (2.9)	22.5 (2.0)	23.5 (5.2)	26.8 (6.8)	24.5 (4.3)
Range	22.0–31.2	20.5–24.5	18.4–35.6	21.2–34.3	18.4–35.6
Current smokers	3 (30%)	0 (0%)	4 (40%)	0 (0%)	7 (27%)

Data are n (%) or mean (SD) unless stated otherwise.

BMI = body-mass index; LD = low dose; HD = high dose; GNP = gold nanoparticles.

### Safety

**Fig. 2:**
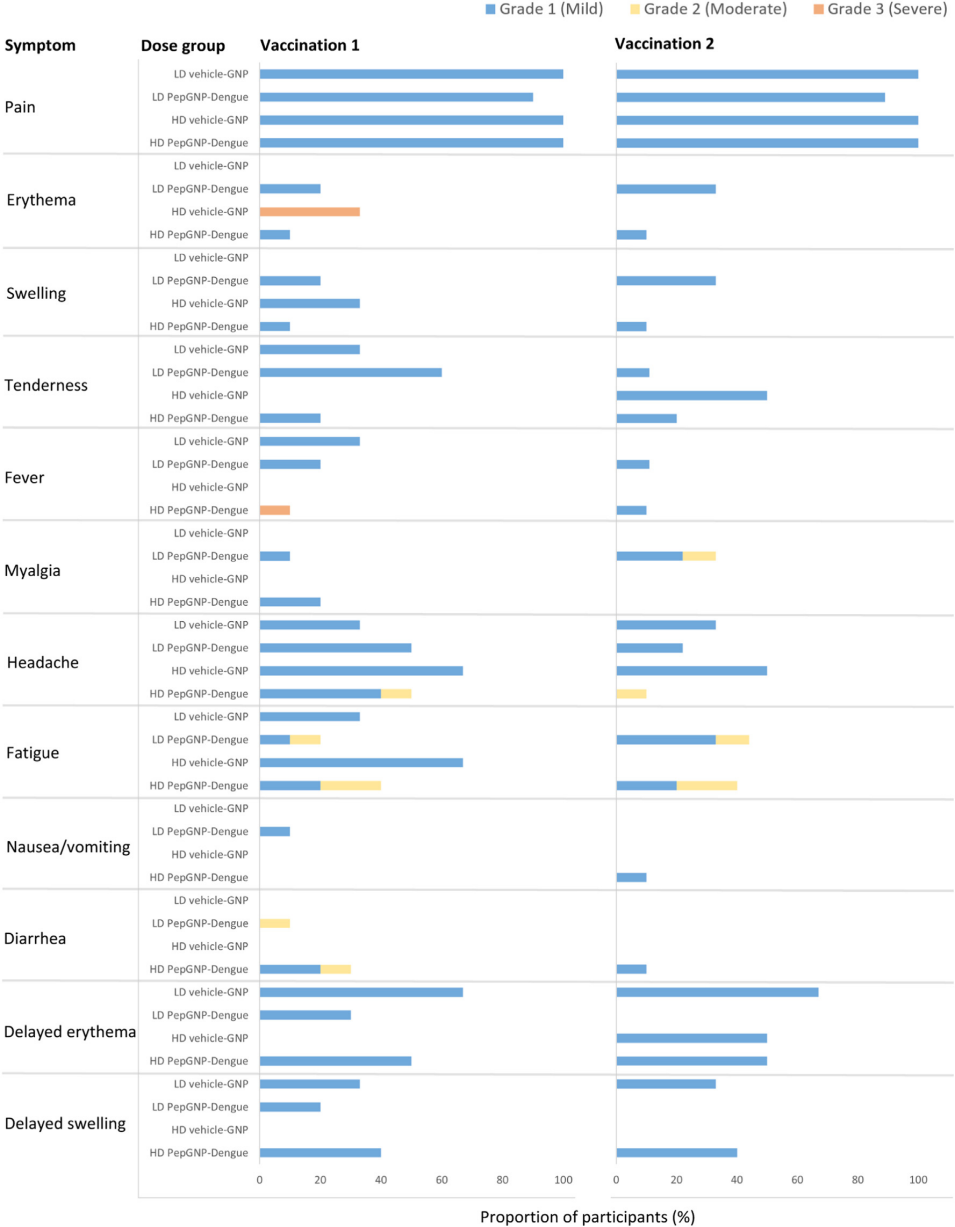
**Local and systemic solicited reactogenicity∗ and delayed erythema and swelling.** n = 3 in the LD and HD vehicle-GNP groups, except for the second vaccination of the HD vehicle-GNP group in which n = 2 (one participant not vaccinated due to grade 3 local erythema after first injection). n = 10 in the LD and HD PepGNP-Dengue groups, except for the second vaccination of the LD PepGNP-Dengue group in which n = 9 (one participant not vaccinated for personal reasons). ∗Solicited local and systemic reactogenicity: signs/symptoms occurring within 7- and 14-days post-vaccination, respectively. GNP = gold nanoparticles; LD = low dose; HD = high dose.

Vaccinations appeared to be safe and well-tolerated. One SAE was reported, an alcohol intoxication, not related to the vaccine (Table 2). No holding rule has been triggered. Most (90%) of the related AEs were mild, and more frequently detected after the first injection than the second. The frequency of AEs was comparable in men and women. Local reactogenicity was similar across the two dosages tested and between PepGNP-Dengue and vehicle-GNP groups (Table 2). Mild injection site pain was the most frequently reported symptom and resolved within 24 h in all cases. Three (30%) participants in the LD PepGNP-Dengue group and one (10%) in the HD PepGNP-Dengue group experienced mild local erythema and/or swelling within seven days after each vaccination (Fig. 2). One participant in the HD vehicle-GNP group developed severe local erythema (≤12 cm diameter) and swelling that peaked five days after vaccination, associated with mild pruritus. Punch biopsy revealed a lymphohistiocytic infiltrate and eosinophils, consistent with an eczematiform reaction. Topical corticosteroid cream and oral antihistamine therapy were administered with rapid improvement. The DSMC advised against second vaccination for this participant at that point.

**Table 2:** Safety profile.

	LD PepGNP-Dengue (n = 10)	LD vehicle-GNP (n = 3)	HD PepGNP-Dengue (n = 10)	HD vehicle-GNP (n = 3)
**Solicited local adverse event**^a^				
Grade 1	10 (100%)	3 (100%)	10 (100%)	3 (100%)
Grade 3	0 (0%)	0 (0%)	0 (0%)	1 (33%)
**Solicited systemic adverse event**^b^				
Grade 1	6 (60%)	3 (100%)	8 (80%)	2 (67%)
Grade 2	3 (30%)	0 (0%)	4 (40%)	0 (0%)
Grade 3	0 (0%)	0 (0%)	1 (10%)	0 (0%)
**Unsolicited local reaction**^c^				
Grade 1	9 (90%)	3 (100%)	10 (100%)	2 (67%)
**Unsolicited systemic reaction**^c^				
Grade 1	8 (80%)	2 (67%)	10 (100%)	3 (100%)
Grade 2	3 (30%)	1 (33%)	2 (20%)	2 (67%)
Grade 3	0 (0%)	0 (0%)	1 (10%)	0 (0%)
**Adverse event of special interest**^c^	0 (0%)	0 (0%)	3 (30%)	1 (33%)
**Serious adverse event**^c^	0 (0%)	1 (33%)	0 (0%)	0 (0%)
**Death**^c^	0 (0%)	0 (0%)	0 (0%)	0 (0%)

Includes participants with at least one clinical or laboratory adverse event. Data are n (%).

LD = low dose; HD = high dose; GNP = gold nanoparticles.

a Within 7 days of any injection.

b Within 14 days of any injection.

c During the 180-day study follow-up.

At least one solicited systemic AE was reported in 15 (75%) participants in the PepGNP-Dengue groups, and in 5 (83%) participants in the vehicle-GNP groups, mostly mild. The most common symptoms were headache (in ten [50%] and four [67%] participants in the PepGNP-Dengue and vehicle-GNP groups, respectively) and fatigue (in nine [45%] and three [50%] participants in the PepGNP-Dengue and vehicle-GNP groups, respectively). Overall, 19% of participants had an axillary temperature ≥37.5 °C within 14 days of each vaccination, including one in the HD PepGNP-Dengue group (39.1 °C) graded as severe, which resolved within 24 h. Grade 2 (moderate) related solicited and unsolicited systemic AEs were more common in the HD [nine (69%) participants] than in the LD group [six (46%) participants], with a similar frequency between the PepGNP-Dengue and vehicle-GNP groups.

With regard to unsolicited related local AEs, 24 (92%) participants reported transient discoloration (slight bluish to greyish, usually one centimetre in diameter) at the injection site (caused by the black-coloured nanoparticles), which spontaneously resolved the day after injection. Three (30%) LD PepGNP-Dengue, two (67%) LD vehicle-GNP, five (50%) HD PepGNP-Dengue, and one (33%) HD vehicle-GNP participants experienced delayed local erythema and/or swelling at one or both injection sites (diameter ≤20 mm in all but one participant [30 mm]), occurring more than seven days (and even several weeks) post-vaccination. Lesions gradually improved spontaneously, but two participants reported a slight transient increase in erythema and/or swelling after another vaccination (influenza or COVID-19). At the last visit (day 180), 14 (54%) participants had persistent local erythema and/or swelling.

The majority of laboratory AEs considered related to the study intervention were mild and not clinically significant. One individual (HD PepGNP-Dengue group) developed a grade 3 increase in liver tests (aspartate and alanine aminotransferase levels), with transient fatigue and abdominal discomfort (both symptoms having been reported only for a few hours on a single day). Values became out-of-range on day 35, peaked on day 43, and fully recovered by day 64. As investigations failed to reveal any alternative aetiology (viral, toxic, autoimmune, or obstructive), the event was considered possibly related to the study product.

Three related AESIs were reported (all grade 3), namely the aforementioned injection site erythema, pyrexia, and transient increase in liver enzymes.

### Immunogenicity

#### Antibody responses

**Fig. 3:**
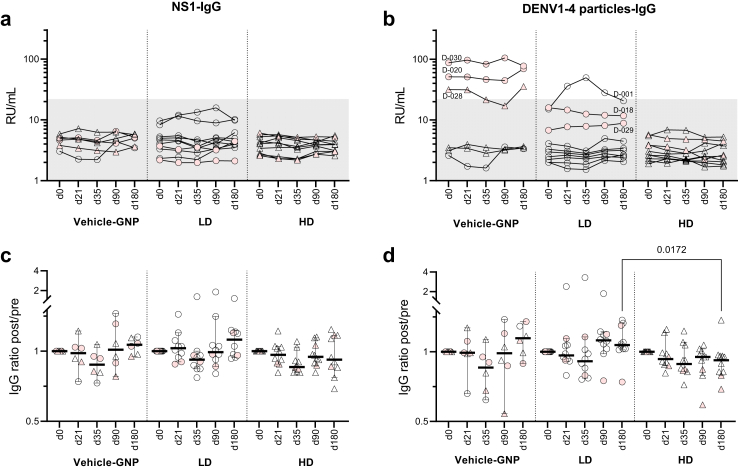
**Anti-DENV1-4 IgG response∗ to PepGNP-Dengue vaccination.** Kinetics of anti-NS1 (a and c) and anti-DENV1-4 particles (b and d) IgG levels are shown in groups vehicle-GNP (n = 6), LD PepGNP-Dengue (n = 10) and HD PepGNP-Dengue (n = 10). In (a and b), antibody titres (RU/ml) above the grey zone are positive (>22 RU/ml). In (c and d), fold change from baseline; bars indicate medians and 95% confidence intervals. Intra-group comparisons with day 0 used non-parametric Friedman tests; inter-group comparisons used non-parametric Kruskal–Wallis tests at each timepoint. *p* values <0.05 are indicated. Pink symbols indicate participants with TBE positive response. ∗Measured by enzyme-linked immunosorbent assay. LD = low dose (○); HD = high dose (▵); GNP = gold nanoparticles; TBE = tick-borne encephalitis.

PepGNP-Dengue did not induce an anti-DENV humoral response, except for one participant. Samples taken pre- and post-vaccination were all negative for anti-NS1 1-4 IgG (Fig. 3a). Positive responses with RDT (one HD PepGNP and one LD vehicle-GNP participant at days 14 and 35, respectively) and ELISA for DENV 1-4 IgG (three vehicle-GNP participants, Fig. 3b) were correlated with previous TBE vaccination (Supplementary Figure S3a). One LD PepGNP-Dengue participant (D-001), not previously TBE vaccinated, seroconverted at day 21 and remained positive for DENV 1-4 IgG at days 35 and 90. The LD PepGNP-Dengue group presented a significant increase in non-specific IgG level (anti-TT), which could explain the seroconversion observed (Supplementary Figure S3b and d).

#### Cell-mediated responses

Out of the 26 participants, 25 volunteers overall (20 of whom received PepGNP-Dengue) had at least one HLA match (i.e., were positive for HLA-A∗02, A∗03, A∗11, A∗24, A∗31, A∗32, B∗07, B∗08, B∗15, B∗27, B∗35 or B∗51, the restriction imposed by the panel of peptides in the vaccine), with the most frequent number of matches across HLA-A and HLA-B alleles being two (Supplementary Figure S4).

**Fig. 4:**
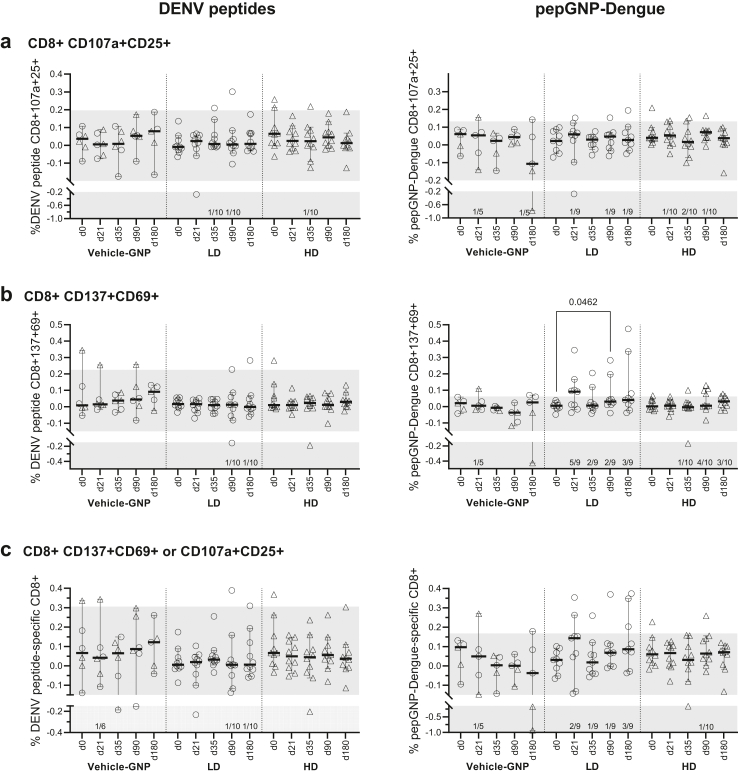
**Dengue-specific CD8+ responses by AIM.** Kinetics of CD8+ responses were evaluated by AIM assay in groups vehicle-GNP (n = 6), LD PepGNP-Dengue (n = 10), and HD PepGNP-Dengue (n = 10). Percentage of antigen-specific CD8+ over total CD8+, stimulated minus unstimulated specific CD8+, defined as expressing activation co-markers CD107a+CD25+ (a), CD137+CD69+ (b) or at least one co-marker (c) upon stimulation with DENV peptides (left panels) or PepGNP-Dengue (right panels). Intra-group comparison with day 0 used Friedman tests, and inter-group comparison used Kruskal–Wallis tests. *p* values <0.05 are indicated. Bars indicate medians and 95% confidence intervals. Number of responders indicated above the x-axis were defined as a volunteer with a positive delta (post- minus pre-vaccination) response and frequency of dengue-specific CD8+ T cell above the mean +2 SD of the AIM+CD8+ T cell response in all volunteers at day 0 (grey zone). LD = low dose (○); HD = high dose (▵); GNP = gold nanoparticles; AIM = activation-induced markers; SD = standard deviation.

AIM was performed on PBMC samples from all participants at all timepoints. Most CD8+ responses tended to increase at day 21 in the LD PepGNP-Dengue group and decline at day 35 in the LD and HD PepGNP-Dengue groups. PepGNP-Dengue induced a statistically significant rise in PepGNP-Dengue-activated CD137+CD69+CD8+ at day 90 compared to baseline in the LD group (+0.0318%, 95% CI: 0.0088–0.1723, Friedman test *p* = 0.046, Fig. 4b). In terms of responders to the vaccine, LD PepGNP-Dengue induced a higher number of responders than HD PepGNP-Dengue at day 21 for CD137+CD69+CD8+ [5/10 (50%) versus 0/10 (0%), Fisher’s test *p* = 0.033, Table 3]. Overall, 60% and 10% of participants in the LD and HD groups, respectively, presented at least one positive CD8+ T-cell co-marker (CD107a+CD25+ or CD137+CD69+) at any timepoint. PP analysis of AIM CD8+ responses did not differ from the ITT analysis described above, except for responder numbers (higher in the LD compared with the HD PepGNP-Dengue group: 6/9 [67%] and 1/10 [10%], respectively; Fisher’s test *p* = 0.020, Supplementary Table S3).

**Table 3:** Dengue-specific responders^c^ in AIM, intention-to-treat analysis.

	Vehicle-GNP (n = 6)					LD PepGNP-Dengue (n = 10)					HD PepGNP-Dengue (n = 10)				
Day	21	35	90	180	Any^a^	21	35	90	180	Any^a^	21	35	90	180	Any^a^
CD8+CD107a+CD25+	1 (17%)	0 (0%)	0 (0%)	1 (17%)	2 (33%)	1 (10%)	1 (10%)	2 (20%)	1 (10%)	3 (30%)	1 (10%)	2 (20%)	1 (10%)	0 (0%)	4 (40%)
CD8+CD137+CD69+	1 (17%)	0 (0%)	0 (0%)	1 (17%)	2 (33%)	5^b^(50%)	2 (20%)	3 (30%)	3 (30%)	6 (60%)	0 (0%)	1 (10%)	4 (40%)	3 (30%)	4 (40%)
At least one co-marker	1 (17%)	0 (0%)	0 (0%)	1 (17%)	2 (33%)	2 (20%)	1 (10%)	2 (20%)	3 (30%)	6 (60%)	0 (0%)	0 (0%)	1 (10%)	0 (0%)	1 (10%)
Any marker+	1 (17%)	0 (0%)	0 (0%)	1 (17%)	2 (33%)	6 (60%)	3 (30%)	3 (30%)	4 (40%)	7 (70%)	1 (10%)	3 (30%)	4 (40%)	3 (30%)	6 (60%)

Number of responders per group is indicated for each dengue-specific CD8+ T cell parameter measured by AIM assay upon stimulation with dengue peptides or PepGNP-Dengue.

LD = low dose; HD = high dose; GNP = gold nanoparticles; AIM = activation-induced markers.

a At any time post-vaccination. Comparison between groups was performed using Fisher’s tests.

b *p* = 0.033, comparison between LD and HD.

c Volunteer with a positive delta (post- minus pre-vaccination) response and a CD8+ T cell response above the mean + 2 SD of the dengue-specific CD8+ T cell response in all volunteers at day 0.

**Fig. 5:**
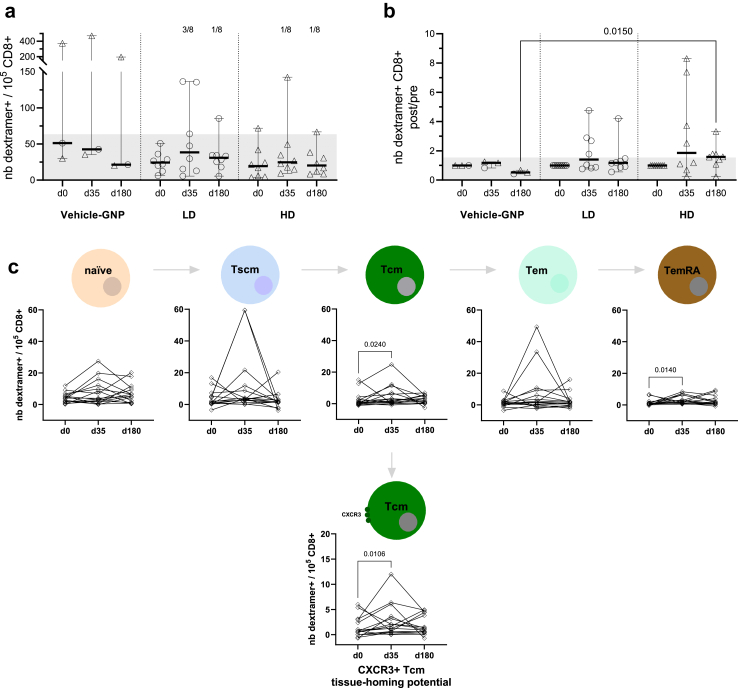
**Dengue dextramer+ CD8+ T cells.** CD8+ responses were evaluated using eight dengue dextramers HLA-A∗02, B∗07, and A∗03 in groups vehicle-GNP (n = 3), LD PepGNP-Dengue (n = 8), and HD PepGNP-Dengue (n = 8) at day 0, 35 and 180. (a) Number of dengue dextramer+ CD8+ T cells over 10^5^ total CD8+ T cells, as the sum of dengue dextramer responses for each participant. Responses to one to seven dengue dextramers per participant were assessed according to their HLA expression and summed. Number of responders are indicated at the top of the panel. (b) Change from baseline as the ratio of post-/pre-vaccination response. (c) Memory phenotype of D-dextramer+CD8+ in vaccinees (LD and HD, n = 16, ◊). Subsets defined as naïve (CD45RA+CCR7+CD95−), Tscm (CD45RA+CCR7+CD95+), Tcm (CD45RA−CCR7+), Tem (CD45RA−CCR7−) and TemRA (CD45RA+CCR7−). Additionally, the functionality of dextramer+ CD8+ T cells was assessed by the expression of CXCR3. Intra-group comparisons with day 0 using Friedman tests and inter-group comparisons used Kruskal–Wallis tests followed with Dunn’s post tests. *p* values <0.05 are indicated. Bars indicate medians and 95% confidence intervals. Grey zones indicate cut-off responses (63.4 dextramer+CD8+/10^5^ CD8+ T cells and a ratio of 1.54). LD = low dose (○); HD = high dose (▵); GNP = gold nanoparticles.

Dengue-specific CD8+ T cells, D-dext+CD8+, were detected using four HLA-A∗0201, one HLA-A∗0301, and three HLA-B∗0702 dengue dextramers in 19 participants carrying the corresponding HLA alleles (eight LD, eight HD, and three vehicle-GNP), at days 0, 35 and 180. PepGNP-Dengue induced a rise in D-dext+CD8+ specific for at least one DENV peptide in 11/16 (69%) participants, 5/8 (63%) in the LD group and 6/8 (75%) in the HD group (Fig. 5b). A significantly greater fold change was found at day 180 in subjects that received HD PepGNP-Dengue compared to those who received vehicle (1.58 (0.24–3.32) versus 0.52 (0.42–0.67), respectively, Kruskal–Wallis *p* = 0.01), although this may have been due to a decrease in response in the latter. Cumulative frequencies of D-dext+CD8+ showed four (25%) responders at day 35, three (38%), and one (13%) in the LD PepGNP-Dengue and HD PepGNP-Dengue groups, respectively, all being negative at day 180 (Fig. 5a). Two late responders at day 180, one in each PepGNP-Dengue group, had the particularity to be HLA-B∗07. The detailed responses per peptide or participant showed the diversity of the level of the responses and the contribution of all peptides to the total response but in different proportions (Supplementary Figure S5). Indeed, considering the HLA-A∗02 restricted D-dextr+CD8+ in 16 participants, peptides LLCVPNIMI and LLGQGPMKLV were dominant, contributing to 70% of the response (Supplementary Figure S6).

As part of an exploratory analysis, vaccine-induced D-dext+CD8+ T cells were further characterized by examining naïve (CD45RA+CCR7+CD95−), stem cell (Tscm, CD45RA+CCR7+CD95+), central (Tcm, CD45RA−CCR7+), effector (Tem, CD45RA−CCR7−), and terminally differentiated (TemRA, CD45RA+CCR7−) memory subsets. All subsets were represented. Participants vaccinated with PepGNP-Dengue (LD and HD) presented an increase of total D-dext+CD8+ memory T cells at day 35 (n = 16, +4.64/10^5^ CD8+, 95% CI: 0.09–35.12, Friedman test *p* = 0.024), specifically Tcm and TemRA subsets (+1.34/10^5^ CD8+, 95% CI: 0.1–7.34, *p* = 0.024 and +0.8/10^5^ CD8+, 95% CI: 0.19–5.13, *p* = 0.014, respectively) (Fig. 5c). The functionality of D-dextr+ CD8+ T cells was assessed by the expression of CXCR3, with a significant increase in CXCR3+ dengue-specific CD8+ Tcm for the PepGNP-Dengue group at day 35 (+1.335/10^5^ CD8+ (0.1–7.34), Friedman test *p* = 0.0106, Fig. 5c). Observed increases in D-dext+CD8+ total, Tcm, TemRA, and CXCR3+ Tcm memory cells in the PepGNP-Dengue group were apparent for the LD but not the HD group.

## Discussion

The distinctive feature of PepGNP-Dengue lies in its design–a synthetic peptide nanoparticle-based vaccine–which aims to specifically elicit, or boost, a CD8+ T cell response, in order to provide protection without the need of vaccine-induced antibodies.

In general, the two formulations (LD and HD) of PepGNP-Dengue appeared to be safe and well tolerated. No vaccine-related SAEs were reported, and the majority of AEs were mild. Interestingly, AEs were more frequently observed after the first injection than after the second. Frequent local reactogenicity (erythema and/or swelling) was noted, in line with previous reports of intradermal dengue vaccine administration.^27^^,^^28^ Participants in the vehicle-GNP groups also reported local AEs and the only severe local reaction occurred in an HD vehicle-GNP participant, indicating that GNPs contributed to local reactogenicity. 11 (42%) participants (both in vaccine and comparator groups) experienced a delayed local erythema and/or swelling, asymptomatic in all but one participant who reported transient grade 1 local tenderness and pruritus. Both reactions faded over the course of the trial (although they were transiently enhanced in two participants, after vaccination against influenza and COVID-19, respectively) and were still visible at the end of the six-month follow-up period for all but one participant. Similar delayed reactions (appearing within a few days or weeks post-injection) were reported in a study using GNPs conjugated with proinsulin peptides administered intradermally,^19^ although, in our study, reactions could be delayed for several weeks even after the second vaccination. The incidence of systemic adverse events (notably headache, fatigue, myalgia, and fever) is comparable to that reported in Phase 1 trials of the two licensed vaccines.^27^^,^^29^ Systemic reactogenicity may be dose-dependent, since mild and moderate reactions were more frequently observed in the HD groups (data not shown). No clinically relevant changes were observed in laboratory measurements, except a unique event of elevated liver enzymes that spontaneously resolved, which could be considered as an event deserving attention in future trials to assess if this was an outlying event.

As expected, PepGNP-Dengue did not induce significant humoral responses against the four DENV serotypes, a result that supports the further development of the present platform for vaccines to avoid/prevent antibody-dependent disease enhancement. Nevertheless, PepGNP-Dengue induced modulation of IgG levels with a vaccine dose-dependent profile. Two types of humoral response were observed: a polyclonal non-specific (bystander) antibody response, particularly in the LD group with an increase in anti-TT IgG, and an antibody response which may result from previous flavivirus exposure or TBE vaccination (and therefore potentially cross-reactive in the anti-dengue IgG ELISA). Indeed, the three participants positive at baseline for DENV IgG by ELISA (all in the vehicle-GNP group) received TBE vaccination one or two years prior. In the absence of previous TBE vaccination, one LD PepGNP-Dengue participant developed anti-DENV IgG, but the baseline level was in the upper level of the normal range, which may suggest prior exposure to flavivirus antigens. The observed seroconversion at day 21 may also be explained by a general increase in IgG (of anti-DENV 1-4, NS1 1-4, TBE, and to a lesser extent of TT IgG), but we cannot exclude a positive humoral response to PepGNP-Dengue as a booster.

As expected, the vaccine candidate elicited dengue-specific cellular immunity, with vaccine peptide-specific CD8+ T cell responses detected in AIM and dextramer analyses. Overall, 67% and 10% of participants in the LD and HD groups respectively, presented positive AIM+CD8+ T-cell at any timepoint. This indicates that the vaccine is able to induce CD8+ T cells with cytolytic activity. Altogether, 38% of participants in the LD group and 13% in the HD group showed at least one positive dextramer response at any timepoint. An absence of statistically significant intra- and inter-group differences in AIM and dextramer analyses except for CD137+CD69+CD8+ at day 90 in the LD group indicates that PepGNP-Dengue induced low-intensity CD8+ T cell responses or involved low percentages of responders at each timepoint. Responder analyses indicated a different time course for markers of activation as compared to peak CD8+ T cell dextramer recognition, and for peak response in the LD and HD PepGNP-Dengue groups, although participant numbers involved were small.

Analysis of D-dext+CD8+ memory T cell subsets showed a significant vaccine-induced increase in the total memory population measurable in peripheral blood, specifically Tcm and TemRA subsets, at day 35. Demonstrating the presence of vaccine-specific memory T cells in trial participants is important, for this is crucial for protection against full-blown disease following (re)encounter with DENV. The specific memory cell subsets induced are of relevance, for Tcm cells can provide long-term, self-renewing, immunological memory, and virus-specific CD8+ TemRA cells were associated with protective immunity against viral challenge in the context of vaccination with a live attenuated tetravalent DENV vaccine.^30^ A significant increase in CXCR3+ dengue-specific CD8+ Tcm at day 35 in the PepGNP-Dengue group indicates that cells with tissue-homing potential were generated. CXCR3 is a CC-chemokine receptor that plays an important role in T cell trafficking to peripheral sites of inflammation and, in the context of a viral infection, expression on CD8+ T cells has been found to enhance their ability to locate infected cells in the skin, thus maximizing cell killing and pathogen clearance.^31^ Taken together, results indicate that PepGNP-Dengue vaccination generates memory T cells in peripheral blood that have the capability to migrate to the skin and potentially form a first line of defence of skin-resident memory T cells (Trm). Whilst Trm cells cannot be measured, a reduction in dengue-specific CD8+ T cells circulation following the second dose could be a sign of their recruitment to the skin. Interestingly, the analysis of the CD8+ responses favours the low dose of PepGNP-Dengue, since both AIM and dextramers results showed more responders in the LD versus HD group. A preference for LD is also supported by the reactogenicity analysis (fewer systemic AEs in the LD group). An even lower dose could be tested in future clinical trials. A comparatively weaker response in the HD group might be explained by increased duration of vaccine retention at the intradermal injection site with a higher dose, with persistent antigen at the vaccination site previously found to induce sequestration of specific CD8+ T cells.^32^

### Caveat and limitations

With regard to limitations of the study, the small sample size precludes any statistical analysis of the safety data and may have limited the statistical significance of any changes in immunogenicity measures. Regarding immunogenicity analysis, one limitation is the use of RDT to exclude prior DENV infection at screening, since its sensitivity for past infections in non-endemic settings has been shown to be low.^33^ Still, ELISA for DENV 1-4 IgG and NS1 were performed at day 0 and were negative in all PepGNP-Dengue recipients. Besides, we cannot formally exclude a possible booster effect due to prior dengue (with waning immunity) or other flaviviruses infection. Indeed, two PepGNP-Dengue recipients, even if negative for DENV 1-4 IgG at baseline, were in the upper level of the normal range, and one of them seroconverted after vaccination. Although cross-reactivity with TBE (endemic in Switzerland) was assessed, this was not the case for the other flaviviruses, for which prior infection cannot therefore be formally ruled out. Nevertheless, given the exclusion criteria for participants (including previous residence for more than 12 months in regions endemic for other flaviviruses), we consider this risk to be very low. Another drawback of the analyses performed is that a potential fraction of the memory CD8+ T cell pool cannot be captured by sampling peripheral blood^34^; this may have contributed to apparently low magnitude CD8+ T cell responses. Another limitation is that the panel of dextramers used could only detect a fraction of vaccine-specific CD8+ populations. Finally, extrapolation of immunogenicity (as indicated by the intensity of vaccine-induced CD8+ T cell responses) to protective efficacy is not possible at this stage in the absence of correlates of immunoprotection.^35^^,^^36^ This could be available in the next phases of clinical development.

Overall, results of this clinical trial of a synthetic nanoparticle-based, T cell priming peptide vaccine against a viral pathogen were positive. Although local mild reactions were frequent, the vaccine appeared to be safe with no vaccine-related SAEs reported. Furthermore, results indicate proof of concept that specific peptides attached to a nanoparticle carrier as a vaccine can successfully induce virus-specific effector and memory CD8+ T cell responses. These findings are encouraging for further clinical trials, involving larger numbers of subjects, to investigate this innovative dengue vaccine candidate.

## Contributors

AM, RA, HMEH, LWD, MF, MAH, FS, and BG contributed to the conception and design of the study. AM, RA, JB, MK, LS, SS, and VF were involved in data collection. Qvigilance, the pharmacovigilance CRO, was responsible for maintaining the safety database and partly carried out the safety analysis. AM, MF and BG analysed and interpreted safety data. RA and FS analysed and interpreted immunogenicity data. AM, RA, JB, MF, and VF accessed and verified the underlying data. AM and RA wrote the manuscript, and all authors contributed to its revision. All authors read and approved the final version of the manuscript.

## Data sharing statement

The clinical study protocol is provided online. According to the research protocol, the participant’s consent, and the Swiss data protection law, the coded data can be shared with the editor and the peer reviewers during the peer-reviewing process to replicate the results of this paper only. However, data cannot be made available for the readers or for other research teams due to restrictions in the previously cited documents. Data dictionary and metadata related to the datasets are available for the readers through the Unisanté data repository, at https://doi.org/10.16909/DATASET/43.

## Declaration of interests

All authors and the PV CRO Qvigilance declare no competing interests.

## Appendix A Supplementary data

Supplementary Material

naNO-DENGUE_ClinicalTrialProtocol_V5.0
