# Supplementary material for: Safety and immunogenicity of a synthetic nanoparticle-based, T cell priming peptide vaccine against dengue in healthy adults in Switzerland: a double-blind, randomized, vehicle-controlled, phase 1 study
Source: eBioMedicine. 2023 Dec 20;99:104922. doi: 10.1016/j.ebiom.2023.104922 (PMC10776924; doi:10.1016/j.ebiom.2023.104922)
Supplement: Supplementary Material [file mmc1.docx]

**Supplementary material**

[Appendix A : Inclusion and exclusion criteria 2](#_Toc141104820)

[Appendix B : Safety holding rules 4](#_Toc141104822)

[Appendix C: Definition of adverse events of special interest 5](#_Toc141104821)

[Appendix D: Methods and results of immunogenicity analysis 6](#_Toc141104823)

[References 15](#_Toc141104824)

Appendix A: Inclusion and exclusion criteria

This study includes healthy men and healthy, non-pregnant, non-breastfeeding women between the ages of 18 and 45 years old who are residing in Switzerland.

| **Inclusion criteria:** | An individual must fulfil all of the following criteria in order to be eligible for trial enrolment:   1. Aged 18 to 45 years on the day of inclusion 2. Participant signed informed consent 3. Residing in Switzerland |
| --- | --- |
| **Exclusion Criteria** | **An individual fulfilling any of the following criteria is to be excluded from enrolment:**   1. Participant is pregnant, lactating, or of childbearing potential^[[1]](#footnote-2)^ 2. Participation in the four weeks preceding the first trial vaccination or planned participation during the present trial period in another clinical trial investigating a vaccine, drug, medical device, or medical procedure 3. Receipt of any vaccine in the four weeks preceding the first trial vaccination (excepting influenza vaccination, which may be received up to two weeks before first study vaccine) or planned receipt of any vaccine in the four weeks following each trial vaccination. 4. Previous vaccination against Japanese encephalitis (JE), Yellow Fever (YF), or any dengue virus vaccine (monovalent or tetravalent) at any time in the past with either a trial vaccine or another vaccine (commercial or investigational) based on medical history 5. Self-reported or documented history of flavivirus (FV) infection (e.g. DENV, YF, WNV, JE, TBE), confirmed either clinically or serologically 6. Receipt of immunoglobulins, blood or blood-derived products in the past 3 months 7. Known or suspected congenital or acquired immunodeficiency; or receipt of immunosuppressive therapy^[[2]](#footnote-3)^ 8. Self-reported or documented seropositivity for human immunodeficiency virus (HIV), hepatitis B natural infection (HBcAb positive serology), or hepatitis C 9. Previous residence for more than 12 months in, or travel in the last 30 days to FV- endemic regions (excluding TBE and WNV) 10. At high risk for dengue infection during the trial^[[3]](#footnote-4)^ 11. Known systemic hypersensitivity to any of the vaccine components (e.g. gold), or history of a life-threatening reaction to vaccines, or to a vaccine containing any of the same substances 12. Current alcohol abuse or drug addiction (reported or suspected) 13. Chronic illness that, in the opinion of the investigator, is at a stage where it might interfere with trial conduct or completion 14. Thrombocytopenia or any coagulation disorder 15. Identified as an Investigator or employee of the Investigator or study centre with direct involvement in the proposed study, or identified as an immediate family member (i.e. parent, spouse, natural or adopted child) of the Investigator or employee with direct involvement in the proposed study (i.e. in the employment of the Tropivac clinic or DFRI unit at Unisanté). 16. Refusal to be informed in the event that relevant results concerning the participant's health are revealed |

| **Exclusion Criteria at the Time of Vaccination**  *(where delayed administration is possible)* | The following events constitute contraindications to the administration of the investigational product on the day of planned vaccination.  The participant must be followed until resolution of the event as with any medical event and may be considered for vaccination at a later date (maximum 14 days later) or withdrawn at the discretion of the Investigator. Delays due to these events do not constitute a protocol deviation.   - Temperature of >37·5°C at the time of vaccination - Acute disease^[[4]](#footnote-5)^ at the time of vaccination - If there is a clinical/epidemiological suspicion of COVID-19 (according to the clinician’s judgment), the participant will be asked to first take a PCR/rapid test for SARS-CoV-2, and the vaccination will be delayed until the result comes back negative and the symptoms have resolved. |
| --- | --- |

Appendix B: Safety holding rules

The safety holding rules that would have resulted in an immediate suspension of the trial (where restart was subject to data safety monitoring committee review and ethics committee approval) are listed below:

| Solicited local or systemic adverse events of grade 3 | > 34% of volunteers^1^ within any group or subgroup develop a Grade 3 solicited local or systemic adverse event beginning within 2 days after vaccination (day of vaccination and one subsequent day) and persisting at Grade 3 for >48 hrs. |
| --- | --- |
| Unsolicited adverse events | > 34% of volunteers^1^ within any group or subgroup develop a Grade 3 unsolicited adverse event (including a laboratory adverse event) that is considered possibly, probably or definitely related to either vaccination and persists at Grade 3  for > 48hrs. |
| Serious adverse event (SAE) | > 34% of volunteers^1^ within any group or subgroup develop an SAE. |
| Suspected Unexpected Serious Adverse Reaction (SUSAR) | A SUSAR related to the investigational peptide vaccination occurs. |
| Death | Death related to the investigational peptide vaccination occurs. |

^1^More than 34% of the 3 members of the pioneer subgroups is ≥ 2/3. More than 34% of the 13 members of the entire dosage group is ≥ 4/13.

Appendix C: Definition of adverse events of special interest

The list of AEs considered of special interest is adapted from the *D2.3 Priority List of Adverse Events of Special Interest: COVID-19* document, published by the SPEAC (Safety Platform for Emergency vACcines), version 2.0, issued on May 25, 2020.

AESI include (exhaustive list):

- Generalized convulsion
- Guillain-Barré Syndrome (GBS)
- Acute disseminated encephalomyelitis (ADEM)
- Thrombocytopenia
- Anaphylaxis
- Vasculitides
- AE grade 3

Appendix D: Methods and results of immunogenicity analysis

1. Methods
2. Vaccine-specific antibody response using ELISA

The humoral immune response induced by the candidate vaccine PepGNP-Dengue was measured at IAL-CHUV by ELISA against denv1-4 and NS1 at days 0, 21, and 35 (visits 2, 6, and 9) in sera from all volunteers.

Two commercial ELISA kits and provider’s protocol were used for the detection of IgG responses to dengue antigens of the 4 types, virus particles, and env in one and NS1 in the other (anti-dengue virus types 1-4 IgG, # EI 266a-9601-1 G; anti-dengue virus NS1 type 1-4 IgG, # EI 266a-9601-2 G; Euro Immun, Lübeck, Germany). Results were expressed as RE/mL of anti-dengue IgG. Limits of negativity and positivity were defined as 16 and 22 RE/mL, respectively. Between these values, the sample was determined as borderline.

The tick-borne encephalitis (TBE) vaccination status of the volunteers was also evaluated in order to assess the possible interference of the existing anti-TBE IgG with the anti-dengue IgG ELISA due to their cross-reactivity. For the detection of IgG responses to TBE antigens, we also used a commercial ELISA kit (FSME/TBE ELISA IgG/IgM, # EC117.00, Virotech Diagnostics, Rüsselheim, Germany). Results were expressed as Virotech units (VE) of anti-TBE IgG. Limits of negativity and positivity were defined as 9 and 11 VE, respectively. Between these values, the sample was determined as borderline.

1. Non-vaccine specific antibody response using ELISA

The analysis of IgG responses to non-vaccine specific antigens was performed using a custom ELISA for tetanus toxoid (TT).

Briefly, microtiter plates (NuncTM Maxisorp, Denmark) were coated at a concentration of 4 µg/mL TT (TT, Pasteur n°2) in PBS or PBS alone, overnight at 4℃ then blocked with PBS-1% BSA-0·05% Tween for 2 hours. Samples and controls were diluted 1:100 then by 2 in series in PBS-0.5% BSA-0.05% Tween. Samples were added at 50 µL per well in quadruplicate, 2 wells on TT-coated and 2 wells on non-coated plates, and incubated for 2 hours at room temperature (RT). After washing, anti-human IgG secondary antibody conjugated to horse radish peroxidase (Agilent, #P0214, RRID:AB_2893418 ), diluted 1:10000 in PBS-0·5% BSA-0·05% Tween, was added to each well and incubated for 1 hour at RT. Bound antibodies were revealed using TMB (3,3′,5,5′-tetramethylbenzidine) substrate (BD TMB Substrate Reagent Set, Fischer Scientific, UK), incubated in the dark for 5 minutes and blocked with 1M H_2_SO_4_. The optical density (OD) of each well was measured at 450 nm with 630nm as reference, using the Infinite M200 PRO plate reader (Tecan Trading AG). Individual OD values from non-coated wells were subtracted. OD results were converted to mIU/mL anti-TT IgG using a standard curve made with a titration from 50 to 0.1mIU/mL of human anti-TT IgG (Tetagam® P, 250 IU/mL, CSL Behring AG, CH) present on all plates. The limit of positivity is defined as 100 mIU/ml of anti-TT IgG.

1. Peptides

Eleven peptides, from dengue or zika, were synthesized by JPT Peptide Technologies, D, and provided by Immudex, DK. (Table S1). Nine of them are included in PepGNP-Dengue vaccine. The two additional peptides are AMLCIPNAII (AMLc) and APTRVVAAEM (APT_10_). AMLc is the natural sequence of denv2 NS2A 1278. Although in AMLs from PepGNP-Dengue, the cysteine in position four was replaced with a serine. APT_10_ is a truncated sequence of the peptide APT_14_ and has a good binding score to HLA-B*07 and therefore can be used in the analysis of dengue-specific CD8+ T cell response using dextramer technology. These peptides have been described as binding to HLA alleles including HLA-A*02, -A*03, -A*11, -A*24, -A*31, -A*32, -B*07, -B*08, -B*15, -B*27, -B*35, or -B*51. Peptides of the right length, therefore, have the possibility of being combined with 1 to 3 different HLAs and thus form 16 possible dextramer-peptide combinations.

**Table S1. Peptides**

| **Peptide** | | **Origin** | | | **HLA type** |
| --- | --- | --- | --- | --- | --- |
| **ID** | **Sequence** | **Virus (strain)** | **Protein** | **Position^$^** |  |
| LLG | LLGQGPMKLV | denv 1 (SV385) | Capsid | 37/38 | A*02/03/24 |
| SPA | SPARLASAI | denv 4 | NS1 | 811/813 | B*07/08/35/51 |
| AMLs | AMLSIPNAII | denv 2 (DKD811) mut. | NS2A | 1278 | A*02/24/B*51 |
| AMLc^§^ | AMLCIPNAII | denv 2 (DKD811) | NS2A | 1278 | A*02/24 |
| LLC | LLCVPNIMI | denv 2 (DS09) | NS2A | 1279 | A*02/24/32/B*51 |
| APT10^§^ | APTRVVAAEM | denv 1*,2,3,4 | NS3 | 1696-98 | B*07/35/51 |
| APT14 | APTRVVAAEMEEAL | denv 1*,2,3,4 | NS3 | 1696-1702 | A*02/03/11/B*07/08 |
| LMR | LMRNKGIGK | zika, denv 2* | NS4A | 2181, 2161-65 | A*03/A11/A31/B*27 |
| KLA | KLAEAIFKL | denv 2 | NS5 | 3049-54 | A*02/03/24/31/32/ B*27 |
| TIT | TITEEIAVQ | denv 2 (thai) | NS5 | 3127-28, 3133(z) | A*02 |
| LVM | LVMKDGRKL | denv 3 (US1999) | NS5 | 3206-08 | A*02/03/24/32/ B*07/15/27 |

^§^Peptides not present in PepGNP-Dengue: AMLc is the original viral sequence / AMLs present in PepGNP-Dengue. APT_10_ had the proper length to be combined with a dextramer HLA-B*07. * with sequence modified for at least one amino acid. **^$^** Positions based on the 3391 amino acids core sequence of DENV.

1. MHC class I dengue dextramers

Eight dengue dextramers (four HLA-A*0201, three B*0702, and one A*0301) plus 17 control dextramers (MHC Dextramer®, Immudex, DK) were used for this series of analyses (Table S2).

**Table S2. Dextramers**

| **Specificity** | | **Fluorochrome** | | |
| --- | --- | --- | --- | --- |
| **HLA** | **Target** | **FITC** | **PE** | **APC** |
| Vaccine-specific | | | | |
| A*0201 | dengue | KLA | AML | LLC |
| A*0201 | dengue |  |  | LLG |
| B*0702 | dengue | LVM | SPA | APT |
| A*0301 | dengue | LMR |  |  |
| Controls | | | | |
| all | (-) | neg | neg | neg |
| A*0201 | CMV, pp65 | NLVPMVATV | NLVPMVATV | NLVPMVATV |
| B*0702 | CMV, pp65 | TPRVTGGGAM | TPRVTGGGAM | TPRVTGGGAM |
| A*0301 | CMV, IE-1 | KLGGALQAK | KLGGALQAK | KLGGALQAK |
| A*0201 | EBV, BRLF1 | GLCTLVAML | GLCTLVAML | GLCTLVAML |
| B*0702 | EBV, EBNA 3A | RPPIFIRRL |  |  |
| A*0301 | EBV, EMNA 3A | RLRAEAQVK |  |  |

1. HLA-typing

Class I HLA typing was performed on whole blood by RT**-**PCR, using the kit LinkSeq HLA ABCDRDQDP SABR (Linkage Bioscience)**.**

**Table S3. List of markers for flow cytometry**

| Marker | Clone | Fluorophore | Supplier | cat # | RRID | Dilution |
| --- | --- | --- | --- | --- | --- | --- |
| CD3 | UCHT1 | A700 | BD | 557943 | [AB_396952](http://antibodyregistry.org/AB_396952) | 20 |
| CD4 | RPA-T4 | PB | BD | 558116 | [AB_397037](http://antibodyregistry.org/AB_397037) | 50 |
| CD8 | SK1 | BV605 | BD | 564116 | [AB_2869551](http://antibodyregistry.org/AB_2869551) | 100 |
| CD45RA | HI100 | BV711 | BD | 563733 | [AB_2738392](http://antibodyregistry.org/AB_2738392) | 100 |
| LiveDead |  | vivid aqua | Thermo Fisher Scientific | L34957 |  | 800 |
| AIM staining |  |  |  |  |  |  |
| CD137 | 4B4-1 | APC | biolegend | 309810 | [AB_830672](http://antibodyregistry.org/AB_830672) | 100 |
| CD69 | L78 | PerCP | BD | 340548 | [AB_400054](http://antibodyregistry.org/AB_400054) | 20 |
| CCR7 | 150503 | FITC | R&D | FAB197F | [AB_2259847](http://antibodyregistry.org/AB_2259847) | 32 |
| CD25 | M-A251 | PE-CY7 | BD | 557741 | [AB_396847](http://antibodyregistry.org/AB_396847) | 20 |
| CD107a | H4A3 | PE | eBioscience | 12-179-42 | AB_10853326 | 100 |
| Dextramer staining | |  |  |  |  |  |
| Dextramer |  | FITC | Immudex |  |  |  |
| Dextramer |  | PE | Immudex |  |  |  |
| Dextramer |  | APC | Immudex |  |  |  |
| CCR7 | 3D12 | BV786 | BD | 563710 | [AB_2738384](http://antibodyregistry.org/AB_2738384) | 100 |
| CD95 | DX2 | PE-cy7 | BD | 561633 | [AB_10894384](http://antibodyregistry.org/AB_10894384) | 50 |
| CXCR3 | g025h7 | PE/Dazzle 594 | biolegend | 353736 | [AB_2564288](http://antibodyregistry.org/AB_2564288) | 50 |

**Figure S1. Gating strategy, AIM analysis.**

PBMC stimulated with SEB.

**Figure S2. Gating strategy, dextramer analysis.**

PBMC stained with positive control class-I dextramers labelled with FITC, PE and APC.

1. Results

**Figure S3. Non-vaccine related IgG response to PepGNP-Dengue vaccination**.

Anti-tick-borne encephalitis (TBE, panels a and c) and anti-tetanus toxoid (TT, panels b and d) IgG levels are shown in groups Vehicle-GNP (n=6), LD PepGNP-Dengue (n=10) and HD PepGNP-Dengue (n=10) at day 0 to day 35. Panels a and b present antibody titres (Virotech units of anti-TBE, VE, or mIU/mL of anti-TT). Responses above the grey zone are positive. Panels c and d present fold change from baseline. Bars indicate medians and 95% CI. Intra-group comparisons using Wilcoxon tests; inter-group comparisons using Kruskal-Wallis tests at each time-point; *p* values <0·05 are indicated. Yellow symbols: volunteer D-001.

**Figure S4 HLA-A and HLA-B allele results**

**Panel a.** HLA-A and HLA-B allele matches. HLA class I typing was performed by RT-PCR on blood samples drawn on Day 0. For each subject in the PepGNP-Dengue (n=10 [LD+HD]) or in Vehicle-GNP (n=6), the number of HLA-A and HLA-B alleles with known/potential binding to vaccine peptides was determined, and the number of subjects with zero to four HLA matches plotted as a distribution chart. **Panel b.** Number of dengue dextramers HLA-A*02 (n=4), A*03 (n=1), or B*07 (n=3) evaluated per volunteer (n=19) according to their HLA typing. One to seven dengue dextramers were evaluated per volunteer.

HLA, human leukocyte antigen; RT-PCR=reverse transcription polymerase chain reaction; LD=low-dose; HD=high-dose; GNP=gold nanoparticles.

**Table S4. Dengue-specific responders** ^ⴕ^ **in AIM, per protocol analysis**

| Group | Vehicle-GNP  (n=5 from day 35) | | | | | LD PepGNP-Dengue  (n=9 from day 35) | | | | | HD PepGNP-Dengue | | | | |
| --- | --- | --- | --- | --- | --- | --- | --- | --- | --- | --- | --- | --- | --- | --- | --- |
| Day | 21 | 35 | 90 | 180 | any^§^ | 21 | 35 | 90 | 180 | any^§^ | 21 | 35 | 90 | 180 | any^§^ |
| CD8+CD107a+CD25+ | 1 (17%) | 0 (0%) | 0 (0%) | 1 (20%) | 2 (40%) | 1 (10%) | 1 (11%) | 2 (22%) | 1 (11%) | 3 (33%) | 1 (10%) | 2 (20%) | 1 (10%) | 0 (0%) | 4 (40%) |
| CD8+CD137+CD69+ | 1 (17%) | 0 (0%) | 0 (0%) | 1 (20%) | 2 (40%) | 5*(50%) | 2 (22%) | 3 (33%) | 3 (33%) | 6 (67%) | 0 (0%) | 1 (10%) | 4 (40%) | 3 (30%) | 4 (40%) |
| At least one co-marker | 1 (17%) | 0 (0%) | 0 (0%) | 1 (20%) | 2 (40%) | 2 (20%) | 1 (11%) | 2 (22%) | 3 (33%) | 6^#^ (67%) | 0 (0%) | 0 (0%) | 1 (10%) | 0 (0%) | 1 (10%) |
| Any marker+ | 1 (17%) | 0 (0%) | 0 (0%) | 1 (20%) | 2 (40%) | 6 (60%) | 3 (33%) | 3 (33%) | 3 (33%) | 6 (67%) | 1 (10%) | 3 (30%) | 4 (40%) | 3 (30%) | 6 (60%) |

Number of responders per group is indicated for each dengue-specific CD8+ T-cell parameter measured by AIM upon stimulation with dengue peptides or PepGNP-Dengue. ^§^ at any time post-vaccination. Comparison between groups was performed using Fisher’s tests. *, *p* = 0·033; ^#^, *p* = 0·020, comparison between LD and HD. ^ⴕ^, Defined as volunteer with a positive delta (post- minus pre-vaccination) response and a CD8+ T cell response above the mean + 2 SD of the dengue-specific CD8+ T cell response in all volunteers at day 0.
LD = low-dose; HD = high-dose; GNP = gold nanoparticles; AIM = activation-induced markers.

**Figure S5 Individual dengue-specific CD8+ responses**.

Individual kinetics of peptide-specific responses were assessed in volunteers from group Vehicle-GNP (n=3), LD PepGNP-Dengue (n=8), and HD PepGNP-Dengue (n=8), using 8 dengue class I dextramers, 4 HLA-A*02 (black and blue lines), 3 HLA-B*07 (red lines) and 1 HLA-A*03 (green lines). Results are expressed as number of dextramer+ CD8+ T cells over 10^5^ total CD8+ T cells. Comparison with day 0 used Friedman tests. Post-test *p* values <0·05 are indicated.

**Figure S6 Repartition of HLA-A*02 dengue-specific CD8+ responses**.

CD8+ responses at day 35 were assessed using HLA-A*0201 class I combined to dengue peptides KLA, AML, LLC, or LLG, in 16 volunteers, eight from HD PepGNP-Dengue, seven from LD PepGNP-Dengue and one from Vehicle-GNP groups. Panel a, individual results are expressed as number of dextramer+ CD8+ T cells over 10^5^ total CD8+ T cells. Panel b, individual proportions of responses to peptides. Bars indicate median and 95% CI. Comparison between peptides used Friedman tests; post-test *p* values <0·05 are indicated.

References

1. Grifoni A, Weiskopf D, Ramirez SI, et al. Targets of T Cell Responses to SARS-CoV-2 Coronavirus in Humans with COVID-19 Disease and Unexposed Individuals. *Cell* 2020; **181**(7): 1489-501.e15.

2. Bowyer G, Rampling T, Powlson J, et al. Activation-induced Markers Detect Vaccine-Specific CD4⁺ T Cell Responses Not Measured by Assays Conventionally Used in Clinical Trials. *Vaccines* 2018; **6**(3).

1. An individual who does not have childbearing potential is defined as a female who is:

   Pre-menarche or post-menopausal for at least 1 year

   Surgically sterile

   Using an effective method of contraception from at least 4 weeks prior to the first vaccination until at least 10 weeks after the last vaccination (up to day 90). Effective contraception methods are described in the appropriate section of the protocol. [↑](#footnote-ref-2)
2. Such as anti-cancer chemotherapy or radiation therapy, within the preceding 6 months; or long-term systemic corticosteroid therapy (prednisone or equivalent for more than 2 consecutive weeks within the past 3 months) [↑](#footnote-ref-3)
3. Participants travelling to countries/regions with high dengue endemic or epidemic activity [↑](#footnote-ref-4)
4. “Acute disease” is defined as the presence of a moderate or severe illness with or without fever according to investigator judgment. All vaccines can be administered to persons with a minor illness such as diarrhoea, mild upper respiratory infection with or without low-grade febrile illness, i.e. axillary temperature of ≤37.5°C. [↑](#footnote-ref-5)
