# Supplementary material for: Safety and immunogenicity of a synthetic nanoparticle-based, T cell priming peptide vaccine against dengue in healthy adults in Switzerland: a double-blind, randomized, vehicle-controlled, phase 1 study
Source: eBioMedicine. 2023 Dec 20;99:104922. doi: 10.1016/j.ebiom.2023.104922 (PMC10776924; doi:10.1016/j.ebiom.2023.104922)
Supplement: naNO-DENGUE_ClinicalTrialProtocol_V5.0 [file mmc2.pdf]

CLINICAL TRIAL PROTOCOL

naNO-DENGUE

A Phase I, double-blind, randomized, vehicle-controlled, dose-finding, safety study of a synthetic nanoparticle-based, T cell priming peptide vaccine against Dengue virus in healthy adults in Switzerland.

naNO-DENGUE: A Phase-I study of a nanoparticle-based peptide vaccine against Dengue virus

---

NOTE: This is stage 1 of a 2-stage adaptive trial investigating the safety of 2 vaccines from a single nanoparticle vaccine platform for emerging diseases

- **Stage 1:** naNO-DENGUE
  A Phase-I study of a nanoparticle-based peptide vaccine against Dengue (**Master** protocol)
- **Stage 2:** naNO-COVID
  A Phase-I study of a nanoparticle-based peptide vaccine against SARS-CoV2 (**Sub**-protocol implemented after interim safety analysis and approval by the DSMC, the CEC and Swissmedic)

Study Type:	Clinical trial with Investigational Medicinal Product (IMP)<br>Master protocol of a 2-stage adaptive trial (Master protocol type)
Study Categorisation:	First in human (ClinO - Category C)
Study Registration:	Clinicaltrials.gov<br>Swiss National Clinical trial Portal (SNCTP via BASEC)
Study Identifier:	naNO-DENGUE
Investigational Product:	**PepGNP-Dengue:** MHC-class-I binding synthetic peptides derived from the dengue virus bound to gold nanoparticles
Protocol Version and Date:	V5.0_04Feb2022

CONFIDENTIAL.

The information contained in this document is confidential and the property of the Sponsor. The information may not - in full or in part - be transmitted, reproduced, published, or disclosed to others than the applicable Competent Ethics Committee(s) and Regulatory Authority(ies) without prior written authorisation from the Sponsor except to the extent necessary to obtain informed consent from those who will participate in the study.

Sponsor:	**Emergex Vaccines Holding Limited**<br>**Dr Athanasios Papadopoulos**<br>4 & 5 Dunmore Court Wootton Road, Abingdon, Oxfordshire, England, OX13 6BH<br>Email: ap@emergexvaccines.com
Sponsor's representative in Switzerland:	**Prof Blaise Genton**<br>**Unisanté:** Department of training, research and innovation & Policlinique de médecine tropicale, voyages et vaccinations<br>Rue du Bugnon 44, 1011 Lausanne, Suisse<br>Email: blaise.genton@unisante.ch<br>T : +41 21 314 49 32 secr. M :+41 79 556 58 68

Version history

naNO-DENGUE_ClinicalTrialProtocol_V2.0_English_13Oct2020	• Accepted by CER-VD<br>• Submitted to Swissmedic
naNO-DENGUE_ClinicalTrialProtocol_V3.0_English_18Dec2020	• Corrected discrepancy in contraception duration withindocument (Now: until day 90, 10 weeks post 2nd vaccination throughout text)<br>• Removed specific laboratory follow-up for thrombocytopenia to allow clinical judgement<br>• Corrected ambiguity in exclusion criteria (Previous residence in FV-endemic zones does not include West Nileor tick borne encephalitis)<br>• The procedure for photographing the injection site is betterdetailed<br>• Unblinding procedure summarised in a figure<br>• Reporting delays between PI and Sponsor/PVCRO defined<br>• Clarifications that the development of DENV antibodies during the study is not considered an adverse event anddoes not affect the study schedule.<br>• Updated study start date<br>• Updated guidelines on the receipt of the COVID-19vaccination
naNO-DENGUE_ClinicalTrialProtocol_V4.0_English_08Apr2021	• Modifications of the markers used to characterize thememory CD8 T cell response<br>• The assessment of T and B cell responses are conductedas essential part of the analysis<br>• Corrected discrepancy in consent reflexion time (72h)<br>• Corrected discrepancy in physical examinations
naNO-DENGUE_ClinicalTrialProtocol_V5.0_English_04Feb2022	• Blood draw for HLA typing at visit 2 instead of visit 1 ; addition of an EDTA tube (2.7 mL) at visits 11 and 12 for Dengue rapid tests and readjustment of the blood volumes collected<br>• Precision of the follow up of non-serious AEs related with the IMP<br>• Photos of the participants stored on the NAS-CHUV

Signature page

Study Title: naNO-DENGUE : A Phase I, double-blind, randomized, vehicle-controlled, dose-finding, safety study of a synthetic nanoparticle-based, T cell priming peptide vaccine against Dengue virus in healthy adults in Switzerland.

The Sponsor, Sponsor's representative in Switzerland and trial statistician have approved protocol V5.0 dated 04 February 2022 and confirm hereby to conduct the study according to the protocol, current version of theWorld Medical Association Declaration of Helsinki, ICH-GCP guidelines and the local legally applicable requirements.

Sponsor: Emergex Vaccines Holding Limited (representative: Dr Athanasios Papadopoulos)

Place, Date | Signature

Sponsor's representative in Switzerland: Prof Blaise Genton

Lausanne, 17.2.2022

Place, Date | Signature

Trial Statistician: Mohamed Faouzi

Place, Date | Signature

Local principal investigator at study site:
I have read and understood this trial protocol and agree to conduct the trial as set out in this study protocol, the current version of the World Association Declaration of Helsinki, ICH-GCP and the local legally applicable requirement.

Site: Unisanté, Lausanne

Principal Investigator: Prof Blaise Genton

Lausanne, 17.2.2022

Place, Date | Signature

Signature page

Study Title: naNO-DENGUE : A Phase I, double-blind, randomized, vehicle-controlled, dose-finding, safety study of a synthetic nanoparticle-based, T cell priming peptide vaccine against Dengue virus in healthy adults in Switzerland.

The Sponsor, Sponsor's representative in Switzerland and trial statistician have approved protocol V5.0 dated 04 February 2022 and confirm hereby to conduct the study according to the protocol, current version of theWorld Medical Association Declaration of Helsinki, ICH-GCP guidelines and the local legally applicable requirements.

Sponsor: Emergex Vaccines Holding Limited (representative: Dr Athanasios Papadopoulos)

Feb 17, 2022	Athanasios Papadopoulos<br>Athanasios Papadopoulos (Feb 17, 2022 11:09 GMT)
Place, Date	Signature

Sponsor's representative in Switzerland: Prof Blaise Genton

Place, Date	Signature

Trial Statistician: Mohamed Faouzi

Place, Date	Signature

Local principal investigator at study site:
I have read and understood this trial protocol and agree to conduct the trial as set out in this study protocol, the current version of the World Association Declaration of Helsinki, ICH-GCP and the local legally applicable requirement.

Site: Unisanté, Lausanne

Principal Investigator: Prof Blaise Genton

Place, Date	Signature

Signature page

Study Title: naNO-DENGUE : A Phase I, double-blind, randomized, vehicle-controlled, dose-finding, safety study of a synthetic nanoparticle-based, T cell priming peptide vaccine against Dengue virus in healthy adults in Switzerland.

The Sponsor, Sponsor's representative in Switzerland and trial statistician have approved protocol V5.0 dated 04 February 2022 and confirm hereby to conduct the study according to the protocol, current version of theWorld Medical Association Declaration of Helsinki, ICH-GCP guidelines and the local legally applicable requirements.

Sponsor: Emergex Vaccines Holding Limited (representative: Dr Athanasios Papadopoulos)

_________________________ _________________________
Place, Date Signature

Sponsor's representative in Switzerland: Prof Blaise Genton

_________________________ _________________________
Place, Date Signature

Trial Statistician: Mohamed Faouzi

17.02.2022 _________________________
Place, Date Signature

Local principal investigator at study site:
I have read and understood this trial protocol and agree to conduct the trial as set out in this study protocol, the current version of the World Association Declaration of Helsinki, ICH-GCP and the local legally applicable requirement.

Site: Unisanté, Lausanne

Principal Investigator: Prof Blaise Genton

_________________________ _________________________
Place, Date Signature

Table of Contents

ABBREVIATIONS **8**
STUDY SYNOPSIS **11**
1. STUDY ADMINISTRATIVE STRUCTURE **24**
1.1 Sponsor 24
1.1.1 Sponsor’s main contact person 24
1.1.2 Sponsor’s alternative contact person 24
1.1.3 Sponsor’s Representative in Switzerland 24
1.2 Investigators 24
1.2.1 Principal investigator 24
1.2.2 Co-Investigator 24
1.2.3 Project coordinator 25
1.3 Statistician 25
1.4 Laboratory 25
1.5 Monitoring institution 26
1.6 Data Safety Monitoring Committee (DSMC) 26
1.7 Any other relevant Committee, Person, Organisation, Institution 26
1.7.1 Pharmacovigilance 26
1.7.2 Local Pharmacy 26
1.7.3 Regulatory Affairs and Project Management Support 27
2. ETHICAL AND REGULATORY ASPECTS **28**
2.1 Study Registration 28
2.2 Categorisation of study 28
2.3 Competent Ethics Committee (CEC) 28
2.4 Competent Authorities (CA) 28
2.5 Ethical Conduct of the Study 28
2.6 Declaration of interest 28
2.7 Patient Information and Informed Consent 29
2.8 Participant privacy and confidentiality 29
2.9 Early termination of the study 30
2.10 Protocol amendments 30
2.10.1 Unforeseen amendments and deviations 30
2.10.2 Prospective amendments 30
3. BACKGROUND AND RATIONALE **31**
3.1 Background 31
3.1.1 The need for a rapidly scalable, highly targeted vaccine platform 31
3.1.2 The need for a Dengue vaccine 31
3.1.3 The need for a targeted CD8+ T cell immunity in Dengue vaccines 31
3.2 Previous human and animal experience 32
3.2.1 Existing Dengue Vaccines 32
3.2.2 Existing T Cell-Specific Vaccines 33
3.2.3 Existing nanoparticle-based vaccines 33
3.3 Investigational product (Dengue Vaccine Candidate) and indication 33
3.3.1 The vaccine candidate: PepGNP-Dengue 33
3.3.2 Peptide Selection 34

3.3.3 Gold nanoparticle delivery system 34
3.3.4 Administration 35
3.4 Experimental experience of vaccine candidate 35
3.5 Dose Rationale 35
3.6 Explanation for choice of comparator 36
3.7 Risk-Benefit Analysis 36
3.7.1 Potential risks and mitigation strategies 36
3.7.2 Potential benefits 37
3.8 Justification of choice of study population 37
4. STUDY OBJECTIVES 39
4.1 Overall objective 39
4.2 Primary objective 39
4.3 Secondary objectives 39
4.4 Exploratory objectives 39
4.5 Safety objectives 39
5. STUDY OUTCOMES 40
5.1 Primary outcome measures 40
5.2 Secondary outcome measures 40
5.2.1 Assess cellular immunogenicity of the candidate vaccine (PepGNP-Dengue) 40
5.2.2 Assess humoral immunogenicity of the candidate vaccine (PepGNP-Dengue) 40
5.3 Other outcomes of interest 41
5.3.1 Exploratory outcome measures 41
6. STUDY DESIGN 42
6.1 General study design and justification of design 42
6.1.1 Intervention allocation 42
6.1.2 Setting 42
6.1.3 Dosages 42
6.1.4 Adaptive design 43
6.1.5 Timing 43
6.2 Methods of minimizing bias 43
6.2.1 Randomisation 43
6.2.2 Blinding procedures 43
6.2.3 Other methods of minimizing bias 43
6.3 Unblinding Procedures (Code break) 44
6.3.1 Unblinding during the trial 44
6.3.2 Unblinding at the end of the trial 44
6.3.3 Unblinding on the DSMC request 44
7. STUDY POPULATION 45
7.1 Eligibility criteria 45
7.1.1 Effective Contraception for Female Volunteers 46
7.1.2 Influenza and COVID vaccinations 46
7.2 Recruitment and Screening 46
7.2.1 Recruitment 46
7.2.2 Screening (Day -60 to -3) 47
7.3 Assignment to study groups 48

7.3.1 Randomisation 48
7.4 Criteria for withdrawal / discontinuation of participants 49
7.4.1 Replacement of withdrawn participants 49
8. STUDY INTERVENTION 50
8.1 Identity of Investigational Medicinal Products 50
8.1.1 Experimental intervention (PepGNP-Dengue) 50
8.1.2 Control intervention (Base particle Comparator, bpC) 50
8.1.3 Packaging, Labelling and Supply 50
8.1.4 Storage conditions 50
8.2 Administration of interventions 50
8.2.1 Experimental intervention (PepGNP-Dengue) 51
8.2.2 Control intervention (bpC) 51
8.3 Dose modifications 51
8.4 Compliance with study intervention 51
8.5 Data Collection and Follow-up for withdrawn participants 51
8.6 Trial specific preventive measures 51
8.6.1 Medications 51
8.6.2 Contraception 51
8.6.3 Vaccines 52
8.7 Concomitant Interventions (treatments) 52
8.8 Vaccine Accountability 52
8.9 Return or Destruction of Study Drug 52
9. STUDY ASSESSMENTS 53
9.1 Table of study procedures and assessments 53
9.2 Assessments of outcomes 53
9.3 Procedures at each visit 56
9.3.1 Intervention (Day 0 and 21) 56
9.3.2 Follow-up (Day 0 to 180) 56
9.3.3 Analyses (during and after the end of recruitment) 56
10. SAFETY 58
10.1 Definition and assessment of adverse and other safety related events 58
10.1.1 Definition of Adverse events (AEs) 58
10.1.2 Assessment of AE 60
10.1.3 Follow up and actions taken in response to AEs 61
10.2 Reporting of serious adverse events (SAE) and other safety related events 61
10.2.1 Reporting of SAEs 61
10.2.2 Reporting of SUSARs 62
10.2.3 Verification and Reporting of holding rule AEs and AESIs 62
10.2.4 Reporting of safety signals 63
10.2.5 Reporting and Handling of Pregnancies 63
10.2.6 Assessment, notification and reporting on the use of radiation sources 63
10.3 Safety reviews 65
10.3.1 Data and Safety Monitoring Committee (DSMC) reviews 65
10.3.2 PV CRO Safety Reviews 65

11. STATISTICAL METHODS 66
11.1 Determination of Sample Size 66
11.2 Safety analysis 66
11.3 Statistical Methods for Primary Endpoints 66
11.4 Immunogenicity analysis 67
12. QUALITY ASSURANCE AND CONTROL 68
12.1 Data handling and record keeping / archiving 68
12.1.1 Case Report Forms 68
12.1.2 Specification of source documents 68
12.1.3 Record keeping / archiving 68
12.2 Data management 68
12.2.1 Data Management System 69
12.3 External monitoring 69
12.4 Audits and inspections 69
12.5 Confidentiality, Data Protection 69
12.6 Storage of biological material and related health data 69
13. PUBLICATION AND DISSEMINATION POLICY 70
14. FUNDING AND SUPPORT 70
15. INSURANCE 70
16. REFERENCES 71
17. APPENDICES 74
17.1 APPENDIX A: Solicited local and systemic AE 74
17.1.1 Solicited Local AE 74
17.1.2 Solicited Systemic AE 74
17.2 APPENDIX B: Severity grading for abnormal laboratory measures 75

ABBREVIATIONS

ADE	Antibody Dependent Enhancement
AIM	Activation-induced marker
AE	Adverse Event
AESI	Adverse event of special interest
ALT	Alanine aminotransferase
APC	Antigen presenting cells
AST	Aspartate aminotransferase
BASEC	Business Administration System for Ethical Committees, (https://submissions.swissethics.ch/en/)
**bpC**	Base particle Comparator (gold nanoparticles (**GNP**) without peptides)
CA	Competent Authority (e.g. Swissmedic)
CEC	Competent Ethics Committee
CH	Switzerland
CHUV	Centre Hospitalier Universitaire Vaudois
CI	Confidence interval
ClinO	Ordinance on Clinical Trials in Human Research *(in German: KlinV, in French: OClin, in Italian: OSRUm)*
CRF	Case Report Form
CTCAE	Common terminology criteria for adverse events
CTU	Clinical Trial Unit
CYD-TDV	Dengvaxia (live-attenuated chimeric Dengue vaccine by Sanofi Pasteur)
**PepGNP-Dengue**	Investigational medicinal product of this study: an MHC class I-binding synthetic peptides derived from the dengue virus bound to gold nanoparticles<br>NOTE: in the IB, the vaccine is referred to interchangeably as<br>• Generically: RNA peptide T-cell vaccine product generically and GNP-P<br>• Specifically: Dengue T-vaccine and PepGNP-Dengue
**DENV**	**Dengue virus (DENV1-4: serotypes)**
DSMC	Data Safety Monitoring Committee
eCRF	Electronic Case Report Form
ELISA	Enzyme-linked immunosorbent assay
ELISPOT	Enzyme-linked immunospot
FBC	Full Blood Count
FV	Flavivirus (Dengue, Zika, Yellow Fever, West Nile, Tick-Borne Encephalitis)
g/G	Grams/Giga
GCP	Good clinical practice
GLP	Good laboratory practice
GMP	Good manufacturing practice
GNP	Gold nanoparticle
H0/H1	Null/Alternative hypothesis

HBV/HCV	Hepatitis B virus / Hepatitis C virus
hCG	Human chorionic gonadotropin (pregnancy hormone)
**HD**	**High dose group**
HIV	Human immunodeficiency virus
HLA	Human leucocyte antigen
HRA	Federal Act on Research involving Human Beings *(in German: HFG, in French:LRH, in Italian: LRUm)*
Ht	Haematocrit
IB	Investigator brochure
ICH	International conference on harmonization
ICS	Intracellular cytokine staining
IgG	Immunoglobulin G
IgM	Immunoglobulin M
IFN-γ	Interferon gamma
IMP	Investigational Medicinal Product
IPC	Infection Prevention control
IRT	Interactive Response Technology
ISO	International Organisation for Standardisation
ISR	Internal safety review
ITT	Intention to treat
IU	International Units
IUD/S	Intrauterine Device/System
JE	Japanese encephalitis
l/L	Litre
**LD**	**Low dose group**
MCV	Mean Corpuscular Volume
mg	Milligram
MHC	Major Histocompatibility Complex
ml/ML	Millilitre
mmol	Millimole
NAS	Network Attached Storage
nmol	Nanomole
PBMC	Peripheral blood mononuclear cell
PI	Principal investigator
PIS	Participant information sheet
POCT	Point-of-care test
PT	Prothrombin time
RBC	Red Blood Cell
SAE	Serious adverse event
SOP	Standard Operating Procedure
SUSAR	Suspected unexpected serious adverse reaction

TBE	Tick-Borne Encephalitis
TMF	Trial Master File
ULN	Upper limit of normal
µmol/µl	Micromole/microliter
vehicle-GNP	Base-Particle Comparator (comprised of a gold nanoparticle)
WFI	Water for injection
WHO	World Health Organization
WNV	West Nile Virus
YFV	Yellow Fever Virus
ZIKV	Zika Virus

STUDY SYNOPSIS

**Sponsor**	Emergex Vaccines Limited
**Study Title**	**naNO-DENGUE:** A Phase I, double-blind, randomized, vehicle-controlled, dose-finding, safety study of a synthetic nanoparticle-based, T cell priming peptide vaccine against Dengue virus in healthy adults in Switzerland
**Short Title / Study ID**	**naNO-DENGUE:** A Phase-I study of a nanoparticle-based peptide vaccine against Dengue virus
**Protocol Version**	V5.0, 04 February 2022
**Trial registration**	• ClinicalTrials.gov registry<br>• Swiss National Clinical trial Portal (SNCTP via BASEC)
**Study category and Rationale**	Category C as per ClinO<br>First in humans Phase I vaccine
**Clinical Phase**	Phase I
**Submission type**	**Adaptive trial**<br>This is the master protocol for a 2-stage study investigating the safety of 2 vaccines from a nanoparticle vaccine platform for emerging diseases:<br>• **Stage 1:** naNO-DENGUE<br>A Phase-I study of a nanoparticle-based peptide vaccine against Dengue virus (**Master** protocol)<br>• **Stage 2:** naNO-COVID<br>A Phase-I study of a nanoparticle-based peptide vaccine against SARS-CoV2 (**Sub**-protocol, implemented after interim safety analyses and approval by DSMC, the CEC and Swissmedic)<br>**The subprotocol for naNO-COVID has been submitted and approved as a prospective amendment.**<br>See **Figure 2** for an overview
**Hypotheses**	1. During the COVID-19 pandemic, Dengue virus disease is likely to cause significant diagnostic confusion and it risks further progression due to disrupted control measures.<br>2. As the SARS-CoV-2 pandemic devastates the world, the need for highly efficient and scalable vaccines is desperately required.<br>3. Peptide vaccines have high potential as a rapidly scalable modular platform for emerging diseases requiring targeted immunological responses.<br>4. Dengue and SARS-CoV-2 (COVID-19) are particularly well suited to this approach.<br><br>This trial aims to test peptide T cell inducing vaccines against these two diseases in a 2-stage adaptive study design.<br>The trial starts with Dengue (for which the construct has currently completed manufacturing) and will continue by adding the SARS-CoV-2 vaccine as soon as the *in vivo* and stability testing for the vaccine construct has been successfully completed according to GLP.

**Background and Rationale**	**The need for a rapidly scalable, highly targeted vaccine platform**<br>The race to develop a novel vaccine for COVID-19 has revealed the need for rapidly scalable vaccines that have reliable immunological targets. Currently, hundreds of vaccines against SARS-CoV2 are in development and generally consist of a cocktail of antigens or live-attenuated pathogens which are injected into humans in the hope that they target the *"right cells in the right way"*. This untargeted approach requires large volumes of peptides to be produced per recipient which negatively impacts scalability.<br>Nanoparticle antigen delivery systems have been developed to enrich specific targeting of immune receptors. These carrier systems are designed to facilitate antigen uptake and processing by antigen presenting cells (APCs), as well as to control antigen release and protect them from premature proteolytic degradation. This more targeted response also allows us to reduce the effective antigen dose (to nanomoles) and mimic a replicating infection with zero risk of developing the infectious disease.<br>A disease for which targeting the *"right cell in the right way"* is particularly important is Dengue viral disease. Dengue is well known for antibody-dependent enhancement, where poorly formed immune responses predispose the individual to severe disease during a second infection.
	**The need for a Dengue vaccine**<br>Dengue is the most frequent and most rapidly expanding arbovirus in the world with an estimated 3.9 billion people at risk and approximately half a billion infections per year. Its reach is expected to expand further in the wake of global warming. This mosquito borne virus typically manifests as non-specific febrile disease with a rash and flu-like symptoms. Around 2% of patients develop severe haemorrhagic complications (severe dengue), which may progress to death in up to 20% of cases if not provided with prompt supportive care.<br>With no specific treatment or vaccine, management of Dengue relies on the Sisyphean task of vector control, and the global failure of this resource-draining approach is reflected in the 400% increase in dengue incidence over the past 15 years despite exponential increase in funding.<br>The WHO lists the need for a safe and efficient Dengue vaccine as an essential pillar of its Global strategy for Dengue Prevention and Control and specify that the ideal vaccine candidate to undertake this task should protect against all four major dengue serotypes, be given as a single dose, have long-term immunity and have no serious adverse effects. Currently there are no vaccine candidates that fulfil these fundamental criteria.
	**The need for a targeted immunity in Dengue vaccines**<br>Natural infection with dengue generally leads to transient homotypic immunity specific to one of the four major disease-causing viral serotypes (DENV 1-4). Contradictory to their intended protective purpose, these homotypic antibodies have been shown to predispose severe outcomes in subsequent heterotypic infections, known as Antibody Dependent Enhancement (ADE). The potentially harmful role of the antibody memory immune response in the dengue disease process poses a major problem to traditional antibody-mediated (MHC class II-specific) vaccine approaches, and there is thus a need for vaccines which harness selective T cell priming. Currently, there is no available vaccine which is universally safe or effective.
	**The urgent need for a Dengue vaccine during the COVID-19 pandemic**<br>The WHO has warned that COVID-19 has disrupted containment efforts of its mosquito vector and further outbreaks are expected as the pandemic flares across the subtropics. Its overlapping symptoms of non-specific febrile disease is likely to cause diagnostic confusion and misallocation of limited COVID-19 resources.

	**The need for a COVID-19 vaccine**<br>Despite drastic quarantine measures, SARS-CoV-2 and its associated disease called COVID-19 continues to propagate and threatens global healthcare systems by saturating their capacity with its high transmissibility and the particularly protracted length of stay needed by those requiring intensive care. Under this strain, medical complications and death rates are expected to rise dramatically without early preventative pharmaceutical intervention. Indeed, once patients advance to ICU, prognosis is poor. There is thus universal consensus on the need for a globally effectivevaccine to prevent transmission, protect against complications and reduce viral shedding.
	**The need for a targeted vaccine for COVID-19**<br>As the immune response to SARS-CoV2 is being elucidated, it is becoming clearer that developing targeted cell-mediated immunological memory may be the difference between severe life-threatening infection and passing self-resolving disease.<br><br>*Further updated information will be given in the subprotocol dedicated to the COVID-19 vaccine (naNO-COVID).*
**Study design**	Double-blind, vehicle-controlled, randomised trial<br><br>This is stage 1 of a 2-stage study investigating the safety of a peptide T cell inducing vaccine platform for emerging diseases<br>• **Stage 1: naNO-DENGUE:** A Phase-I study of a nanoparticle-based peptide vaccine against Dengue<br>• **Stage 2: naNO-COVID:** A Phase-I study of a nanoparticle-based peptide vaccine against SARS-CoV2<br><br>This protocol serves as the master protocol for stage 2 which has been submitted for review to the relevant Ethics Committee and Swissmedic and commenced conditionally to an interim safety analysis of stage 1 and a review of the subprotocol by the CEC, Swissmedic and the DSMC.<br>i.e. a prospective major amendment in the form of a subprotocol for naNO-COVID has been submitted along with preliminary safety data from stage 1.
**Objective(s)**	**Primary:** To evaluate the safety and reactogenicity of two intradermal injections of two different doses of the investigational Dengue peptide T cell inducing vaccine (**PepGNP-Dengue**) administered to healthy volunteers as a<br>1) candidate vaccine for the prevention of Dengue<br>2) proof-of-concept for a rapidly scalable modular peptide vaccine platform, which will be followed by a COVID-19 construct after interim analyses.<br><br>**Secondary:**<br>1) To assess the evidence of a T-cell mediated immune response as a surrogate of protection against severe dengue disease using a novel peptide set point vaccine in healthy adults.<br>2) To check the absence of an antibody mediated response

<table>
<tr>
<td>Outcome(s)

See Table 1</td>
<td>Primary: Assess the safety, tolerability and reactogenicity of the candidate vaccine (PepGNP-Dengue)

Occurrence of <u>solicited local reactogenicity</u> signs and symptoms
[Time Frame: 7 days following each vaccination]
Occurrence of <u>solicited systemic reactogenicity</u> signs and symptoms
[Time Frame: 14 days following each vaccination]
Occurrence of <u>unsolicited adverse events</u>
[Time Frame: 6 months following enrolment. i.e. entire trial period]
Occurrence of <u>serious adverse events</u> (SAEs)
[Time Frame: 6 months following enrolment. i.e. entire trial period]
Occurrence of <u>adverse events of special interest</u> (Section 10.1.1.3)
[Time Frame: 6 months following enrolment i.e. entire trial period]
Change from baseline for <u>safety laboratory measures</u> (haematology and biochemistry blood results)
[Time Frame: 6 months following enrolment i.e. entire trial period]

Note: all AEs will be recorded throughout the study according to Swiss ClinO ordinance. The timelines above refer only to outcome measures.</td>
</tr>
<tr>
<td></td>
<td>Secondary:

Assess cellular immunogenicity of the candidate vaccine (PepGNP-Dengue):
Proportion of participants with CD8-T cell specific to PepGNP-Dengue
[Time Frame: 6 months following enrolment i.e. entire trial period]
Assess the humoral immunogenicity of the candidate vaccine (PepGNP-Dengue):
Proportion of participants becoming seropositive (antibodies against Dengue virus) i.e. at potential risk for antibody-mediated disease enhancement
[Time Frame: 6 months following enrolment i.e. entire trial period]
</td>
</tr>
</table>

Table 1. *Monitoring periods for safety outcomes*

Day of study	0	7	14	21	28	35	180
	1st injection			*2nd injection*			
**Solicited local reactogenicity**	7 days			7 days			
**Solicited systemic reactogenicity**	14 days			14 days			
**Unsolicited adverse events**	Duration of study						
**SAE**	Duration of study						
**Safety laboratory measures**	Duration of study						
**Cellular immunogenicity**	Duration of study						
**Humoral immunogenicity**	Duration of study						
**All adverse events recorded**	Duration of study						

**Inclusion criteria**	An individual must fulfil all of the following criteria in order to be eligible for trial enrolment:<br>1. Aged 18 to 45 years on the day of inclusion<br>2. Participant signed informed consent<br>3. Residing in Switzerland
**Exclusion Criteria**	**An individual fulfilling any of the following criteria is to be excluded from enrolment:**<br>1. Participant is pregnant, lactating, or of childbearing potential[1]<br>2. Participation in the 4 weeks preceding the first trial vaccination or planned participation during the present trial period in another clinical trial investigating a vaccine, drug, medical device, or medical procedure<br>3. Receipt of any vaccine in the 4 weeks preceding the first trial vaccination (excepting influenza vaccination, which may be received up to 2 weeks before first study vaccine)or planned receipt of any vaccine in the 4 weeks following each trial vaccination.<br>4. Previous vaccination against Japanese encephalitis (JE), Yellow Fever (YF), or any dengue virus vaccine (monovalent or tetravalent) at any time in the past with either a trial vaccine or another vaccine (commercial or investigational) based on medical history<br>5. Self-reported or documented history of flavivirus (FV) infection (e.g. DENV, YF, WNV, JE, TBE), confirmed either clinically or serologically<br>6. Receipt of immunoglobulins, blood or blood-derived products in the past 3 months<br>7. Known or suspected congenital or acquired immunodeficiency; or receipt of immunosuppressive therapy[2]<br>8. Self-reported or documented seropositivity for human immunodeficiency virus (HIV), hepatitis B natural infection (HBcAb positive serology), or hepatitis C<br>9. Previous residence for more than 12 months in, or travel in the last 30 days to FV-endemic regions (excluding TBE and WNV)<br>10. At high risk for dengue infection during the trial[3]<br>11. Known systemic hypersensitivity to any of the vaccine components (e.g. gold), or history of a life-threatening reaction to vaccines, or to a vaccine containing any of the same substances<br>12. Current alcohol abuse or drug addiction (reported or suspected)<br>13. Chronic illness that, in the opinion of the investigator, is at a stage where it might interfere with trial conduct or completion<br>14. Thrombocytopenia or any coagulation disorder<br>15. Identified as an Investigator or employee of the Investigator or study centre with direct involvement in the proposed study, or identified as an immediate family member (i.e., parent, spouse, natural or adopted child) of the Investigator or employee with direct involvement in the proposed study (i.e. in the employment of the Tropivac clinic or DFRI unit at Unisanté).<br>16. Refusal to be informed in the event that relevant results concerning the participant's health are revealed

[1] An individual who does **not** have childbearing potential is defined as a female who is:

- Pre-menarche or post-menopausal for at least 1 year
- Surgically sterile
- Using an effective method of contraception from at least 4 weeks prior to the first vaccination until at least 10 weeks after the last vaccination (up to day 90). Effective contraception methods are described in the appropriate section.

[2] Such as anti-cancer chemotherapy or radiation therapy, within the preceding 6 months; or long-term systemic corticosteroid therapy (prednisone or equivalent for more than 2 consecutive weeks within the past 3 months)

[3] Participants travelling to countries/regions with high dengue endemic or epidemic activity

**Exclusion Criteria at the Time of Vaccination**<br>*(where delayed administration is possible)*	The following events constitute contraindications to the administration of the investigational product on the day of planned vaccination.<br>The participant must be followed until resolution of the event as with any medical event and may be considered for vaccination at a later date (maximum 14 days later) or withdrawn at the discretion of the Investigator. Delays due to these events do notconstitute a protocol deviation.<br>• Temperature of >37.5°C at the time of vaccination<br>• Acute disease[4] at the time of vaccination<br>• If there is a clinical/epidemiological suspicion of COVID-19 (according to the clinician's judgement), the participant will be asked to first take a PCR/rapid test for SARS-CoV2, and the vaccination will be delayed until the result comes back negative and the symptoms have resolved.
**Measurements and procedures**<br>*See **Figure 2** for full overview*	**Recruitment:** This study includes healthy adults aged 18-45 years residing in Switzerland.<br>**Information and consent:** All individuals showing interest in participating in the study will be provided with an information sheet and given a minimum of 72 hours to decide whether they would like further information from the study investigator and/or sign the consent if willing.<br>**Screening:** Consenting participants will then schedule a screening appointment where they undergo eligibility testing comprising a structured interview on their medical history as well as a targeted physical exam. Blood and urine samples will also be collected for clinical laboratory tests which include general assessments of organ function[5] as well as screening for a panel of infectious diseases [6]. Specifically, all eligible participants will be screened for Dengue exposure[7].<br>All eligible female participants will undergo a human chorionic gonadotropin β-subunit (βhCG) urine pregnancy test before receiving any vaccination (performed again on the day of proposed vaccinations to ensure unchanged pregnancy status).<br>**Randomisation:** 26 eligible participants will be randomized in the following groups:<br>• **Group 1** (n=13): 10 Low Dose (LD) PepGNP-Dengue (2.5 nmol peptide + 14.8ug GNP) + 3 LD Base Particle Comparator (14.8ug GNP)<br>• **Group 2** (n=13): 10 High Dose (HD) PepGNP-Dengue (7.5 nmol + 44.5ug GNP) + 3 HD Base Particle Comparator (44.5ug GNP)<br>Thus, 20/26 vaccine vera and 6/26 Base Particle Comparator (bpC). Allocations of vaccine vera vs bpC for each group are double-blinded.<br>Enrolment will follow a dose escalation strategy (LD--> HD) conditional to a Go/No Go by DSMC review.<br>**Intervention:** Two intradermal injections of the IMP or control will be administered in the upper arm of each participant. The injections will take place on d0 and d21 using the Nanopass MicronJet600 (https://www.nanopass.com/product/) microneedle. The participant will be monitored for 60min after each vaccination for immediate adverse reactions.<br>Before any vaccination, a baseline blood sample and medical history, will be taken.<br>**Follow-up:** In total the participant will have 12 contacts with study staff during the trial.<br>• 1 screening visit with blood draw (any time during a 60day period before enrolment). Note that there must be 2 contacts during the screening visit to allow a minimum of 72 hours between receiving the oral + written information and

[4] "Acute disease" is defined as the presence of a moderate or severe illness with or without fever according to investigator judgment. All vaccines can be administered to persons with a minor illness such as diarrhoea, mild upper respiratory infection with or without low-grade febrile illness, i.e. axillary temperature of ≤37.5°C.

[5] Such as full blood count [FBC], alanine aminotransferase [ALT], aspartate aminotransferase [AST], total bilirubin, serum creatinine Urine samples will be tested for the presence of protein, blood, and glucose

[6] Such as human immunodeficiency virus (HIV), hepatitis B virus (HBV), hepatitis C virus (HCV)

[7] Specifically, serologies will be performed Dengue and an oral history will be taken for Zika virus (ZIKV), Japanese encephalitis (JE) and yellow fever (YF) with cross checking in vaccine cards and travel history.

A dengue rapid diagnostic test (combo test, IgG/IgM+NS1) will be performed before and after immunization to assess the usefulness of this exam for evaluating antibody generation in the context of future dengue vaccine trials.

	signing the consent form. The first of these contacts may be telephonic in which case the information sheet is emailed to the interested party.<br>• 2 vaccinations with pre-vaccination blood draws (day 0 and 21)<br>• 3 telephone calls (2 taking place 24 hours after each vaccination: day 1 and 22 and one on day 60). These calls may be transformed into a physical consultation if deemed medically necessary.<br>• 6 physical consultations with blood draws (day 7, 14, 28, 35, 90 and 180)<br>**See Figure 1** for summary schedule, and **See Table 2** for full schedule<br>Blood samples will undergo various safety and immunological testing.<br>All fevers or flu like illnesses will be assessed for COVID-19. SARS-CoV-2 antibody titres will also be taken at several points in the study. Specifically, all fevers ≧38°C, or clinical syndromes meeting local testing criteria in the Canton of Vaud will be investigated for COVID with PCR or rapid test.
**Study Product / Intervention**	**Active substance**<br>• PepGNP-Dengue: A synthetic T cell priming setpoint modifying Dengue virus vaccine composed of ultrasmall carbohydrate-passivated gold nanoparticles carrying covalently bound MHC class I-binding peptides derived from the Dengue virus ligandome.<br>• *Please note that the COVID-19 construct will be presented in a later sub-protocol for review by the DSMC, the CEC and Swissmedic.*<br>**Diluent**<br>• Water for injection (WFI, 50 µl)
	Two doses will be tested sequentially in a risk minimising, dose-escalation strategy:<br>• **Low Dose (LD):** 2.5 nmol total peptide/dose with 14.8ug gold base particle in 50 µl WFI<br>• **High Dose (HD):** 7.5 nmol total peptide/dose with 44.5ug gold base particle in 50 µl WFI
**Comparator Intervention**	**Substance**<br>• Base Particle Comparator (bpC) comprised of a gold particle<br>**Diluent**<br>• Water for injection (WFI, 50 µl)
	Two dosages will be used to match the GNP dose in LD and HD vaccine vera:<br>• **Low Dose (LD) vehicle-GNP:** 14.8 ug in 50 µl WFI<br>• **High Dose (HD) vehicle-GNP:** 44.5 ug in 50 µl WFI
**Administration**	Two intradermal injections of the IMP or vehicle-GNP will be administered in the upper arm of each consenting participant. The injections will take place on day 0 (first injection) and day 21 (second/booster injection) using the Nanopass MicronJet600 (https://www.nanopass.com/product/) microneedle.

Number of Participants with Rationale

This study will enrol 26 participants (20 vaccine vera and 6 Base-Particle Comparator [**vehicle-GNP**] split 50:50 between dosage groups as depicted below.

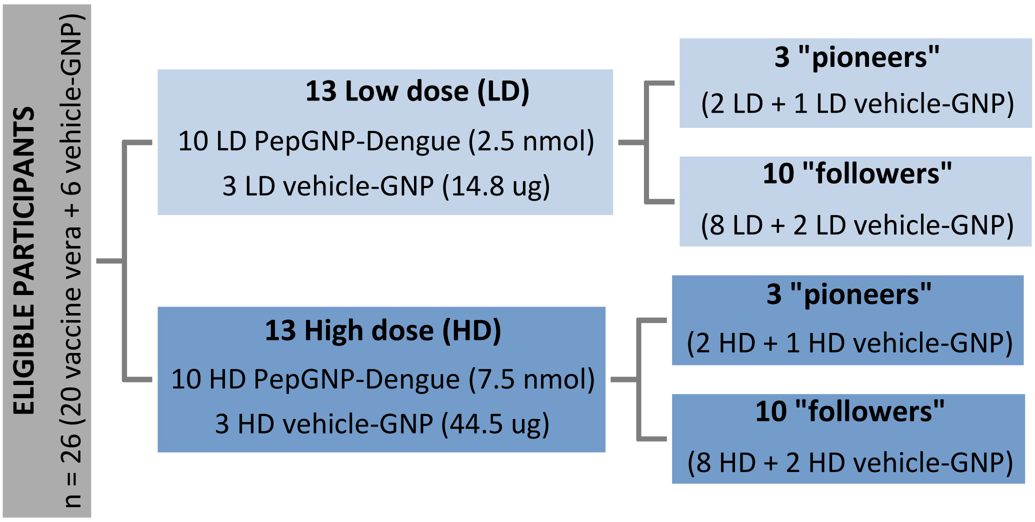

Since this is a first-in-human study with a focus on safety, participant numbers are limited to power the detection of adverse events with high incidence rates. See statistical considerations below.

Statistical Considerations

Having thirteen participants per group (with in total 20 exposed to the investigational product at either dose), would allow 80% power of detecting an AE with a true incidence of:

- 5% across all exposed participants (LD and HD combined) or
- 20% within a single dose group (LD or HD)

Achievable statistical power (1-β) to observe at least 1 AE at various incidences (λ) within the investigational sample size

True incidence of the AE (λ)	Sample size required to detect a single AE at the statistical power (1-β) listed below		
	**50%**	**80%**	**95%**
**2.5%**	28	64.4	120
**5%**	14	32.2	60
**10%**	7	16.1	30
**20%**	3.5	8.05	15
**30%**	1.75	4.03	7.5

KEY

At least a single ***dose-specific*** AE would be detectable at the given probability within a single dose group of 10 participants (either LD or HD)
At least a single ***exposure-dependent*** (any dose) AE would be detectable at the given probability across all dose groups of 20 participants
AE not detectable at this probability within the investigational sample size

	An **interim analysis** by the DSMC will be undertaken with at least 7 days of follow-up information on at least 10/13 of the participants in the LD group, according to **Figure 2**.<br>If no stopping rules are activated **(Table 4)** the DMSC will review the merits for<br>1) Continuing with a second vaccination within the dosage group<br>2) Escalating the dose of naNO-DENGUE from LD→ HD (i.e. enrolling 3 pioneer HD participants)<br>3) Starting enrolling patients in the naNO-COVID sub-study as soon as the subprotocol is approved by EC and Swissmedic.
**Study Duration:**	6 months of enrolment for each participant (180 days)
**Study Schedule:**	• **July 2021:** first participant in for naNO-DENGUE<br>• **Nov 2021:** submission of subprotocol for naNO-COVID and interim analysis of low dose naNO-DENGUE with adaptation to enrol participants for low dose naNO-COVID<br>• **March 2022:** last participant out for naNO-DENGUE<br>• **September 2022:** last participant out for naNO-COVID
**Investigator(s):**	**Overall Coordination**<br>- **Principal investigator:** Prof. Blaise Genton, MD PhD<br>- **Co-investigator:** Dr Mary-Anne Hartley, MD PhD MPH<br>- **Immunological evaluator:** Prof. François Spertini, MD<br>- **Project coordinator:** Dr Alix Miauton, medical physician
**Study Centre(s):**	This is a clinical trial conducted at a single site in Switzerland<br>- Center for primary care and public health, Unisanté & Clinical Trial Unit, Centre Hospitalier Universitaire Vaudois (CHUV), Lausanne, Switzerland
**Compensation:**	Financial compensation will be provided for participation (800CHF/participant) and travel costs will also be covered from their place of domicile.<br>See **Table 2** for payment schedule
**GCP Statement:**	This study will be conducted in compliance with the protocol, the current version of the Declaration of Helsinki, the ICH-GCP (as far as applicable) as well as all national legal and regulatory requirements.

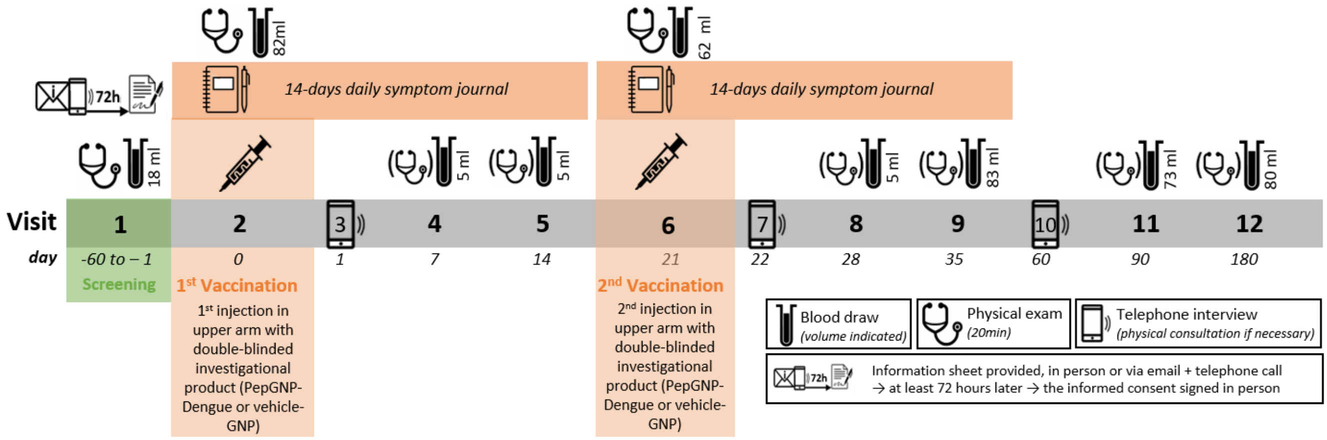

Figure 1: Summarised 180-day schedule of study procedures for a single participant

Table 2: Schedule of study procedures

Visits	1 Screening	2 Vaccination 1	3	4	5	6 Vaccination 2	7	8	9	10	11	12
Timeline (days)	Any time from -60 to -1	0	1	7	14	21	22	28	35	60	90	180
Tolerance (days)		0	±1	±1	±2	±2	±1	±1	±2	±7	±14	±14
**Summary of interventions**												
**Screening and trial procedures**												
**1. Inclusion/Exclusion criteria**	✓	✓ *(confirmation)*				✓						
**2. Informed consent**	✓	✓ *(confirmation)*										
**3. Medical history**	✓	✓ *(confirmation)*				✓				✓	✓	✓
**4. Concomitant medication**	✓	✓ *(confirmation)*	✓	✓	✓	✓	✓	✓	✓	✓	✓	✓
**5. Physical exam**	✓	✓ *(confirmation)*	(✓)	(✓)	(✓)	✓	(✓)	(✓)	(✓)	(✓)	(✓)	(✓)
**6. Compensation (CHF)**	0	100	0	100	100	100	0	100	100	0	100	100
**Intervention**												
**7. Study Vaccination**		✓				✓						
**Safety monitoring** *(Safety monitoring is continuous. Opt-in reporting is active on all days throughout study)*												
**8. Provide diary card**		✓ (1st)				✓ (2nd)						
**9. Collect diary card + photo (if any)**					✓ (1st)				✓ (2nd)			
**10. Solicited local reactogenicity**		✓	✓	✓		✓	✓	✓				
**11. Solicited systemic reactogenicity**		✓	✓	✓	✓	✓	✓	✓	✓			
**12. Review AE/SAE/AESI**		✓	✓	✓	✓	✓	✓	✓	✓	✓	✓	✓
**Samples taken for blood safety tests and serology**												
**13. Complete blood count**	2.7 ml			2.7 ml	2.7ml			2.7ml	2.7ml			
**14. Biochemistry**	2.6 ml			2.6ml	2.6ml			2.6ml	2.6ml			
**15. HIV, HBV, HCV**	4.9 ml											
**16. Dengue rapid tests** [a]	✓ POCT				✓				✓		2.7 ml	2.7 ml
**17. SARS-CoV2 test (PCR/rapid test)**		✓				✓						
**18. SARS-CoV2 serology rapid test**	✓ (POCT)				✓				✓		✓	✓
**19. βHCG, urine test**	✓ (urine)	✓ *(pre-vaccine)*				✓ *(pre-vaccine)*						
**20. Contraception**	✓	✓	✓	✓	✓	✓	✓	✓	✓	✓	✓	
**21. Urinary protein, blood, glucose**	✓ (urine)											
**22. Anti-nuclear antibodies** [b]	7.5 ml										7.5ml	

**Samples taken for secondary immunogenicity studies**												
**23. EDTA- K blood (HLA typing)**		4.9ml										
**24. PBMC & plasma**		67.5ml *(pre-vaccine)*				52.5ml *(pre-vaccine)*			67.5ml		52.5 ml	67.5 ml
**25. Serum (Antibody tests)**		9.8 ml *(pre-vaccine)*				9.8 ml *(pre-vaccine)*			9.8ml		9.8ml	9.8 ml
**Secondary Immunogenicity Outcomes**												
**26. Anti-denv2 IgG response**		✓				✓			✓		✓	✓
**27. CD8 cell response** [c, d]		✓				✓			✓		✓	✓
**Exploratory Immunogenicity Outcomes**												
**28. Ig functional assay** [e]		(✓)				(✓)			(✓)		(✓)	(✓)
**29. T and B cell responses assessment** [f]		(✓)				(✓)			(✓)		(✓)	(✓)
**TOTALS**												
**Summary of interventions**												
**Daily vol (ml)**	17.7	82.2	0	5.3	5.3	62.3	0	5.3	82.6	0	72.5	80
**Max cumulative vol (ml)**	17.7	99.9	99.9	105.2	110.5	172.8	172.8	178.1	260.7	260.7	333.2	413.2
**Compensation cumulative (CHF)**	0	100	100	200	300	400	400	500	600	600	700	800

Legend for Table 2

[a] Detection of IgG and IgM against the 4 DENV serotypes at D0 as an exclusion criterion. Those performed on D35, D90 and D180 are for immune response assessment. The development of DENV antibodies during the study period or in response to the study vaccination is not considered an adverse event and does not affect the participant's study schedule.

[b] As a surrogate for autoimmune reactions. A 4 fold increase from baseline along with a positive history of clinical signs/symptoms in line with autoimmunity, will be investigated as an adverse event.

[c] Frequency of peptide-specific CD8+ T cells on PBMCs by cytometry *ex vivo,* using staining with dengue specific dextramers, CD3, CD4, CD8 and memory markers (CD45RA, CCR7) at D0, D35 and D180;

[d] Frequency of peptide-specific CD8+ T cells by cytometry using activation-induced markers (AIM) CD69, CD137 and CD107a upon stimulation with dengue peptides, at D0, D21, D35, D90 and D180.

[e] Serum Ig functional assay, neutralization and enhancement, performed if positive DENV rapid test and/or anti-denv2 ELISA.

[f] Additional exploratory analyses of dengue-specific cellular responses at various time-points: Additional characterization of the vaccine induced response, cytokine secretion, dengue-specific cell functional assays.

	Physical consultation at study site
	Blood draw
	Vaccination
	Telephonic consultation

✓	Performed
(✓)	Performed and adjusted according to the results obtained in 26 and 27.
+**photo if any**	In the first 7 days post-vaccination participant will be asked to take a photo of the injection site in the event of signs/symptoms of local reactogenicity

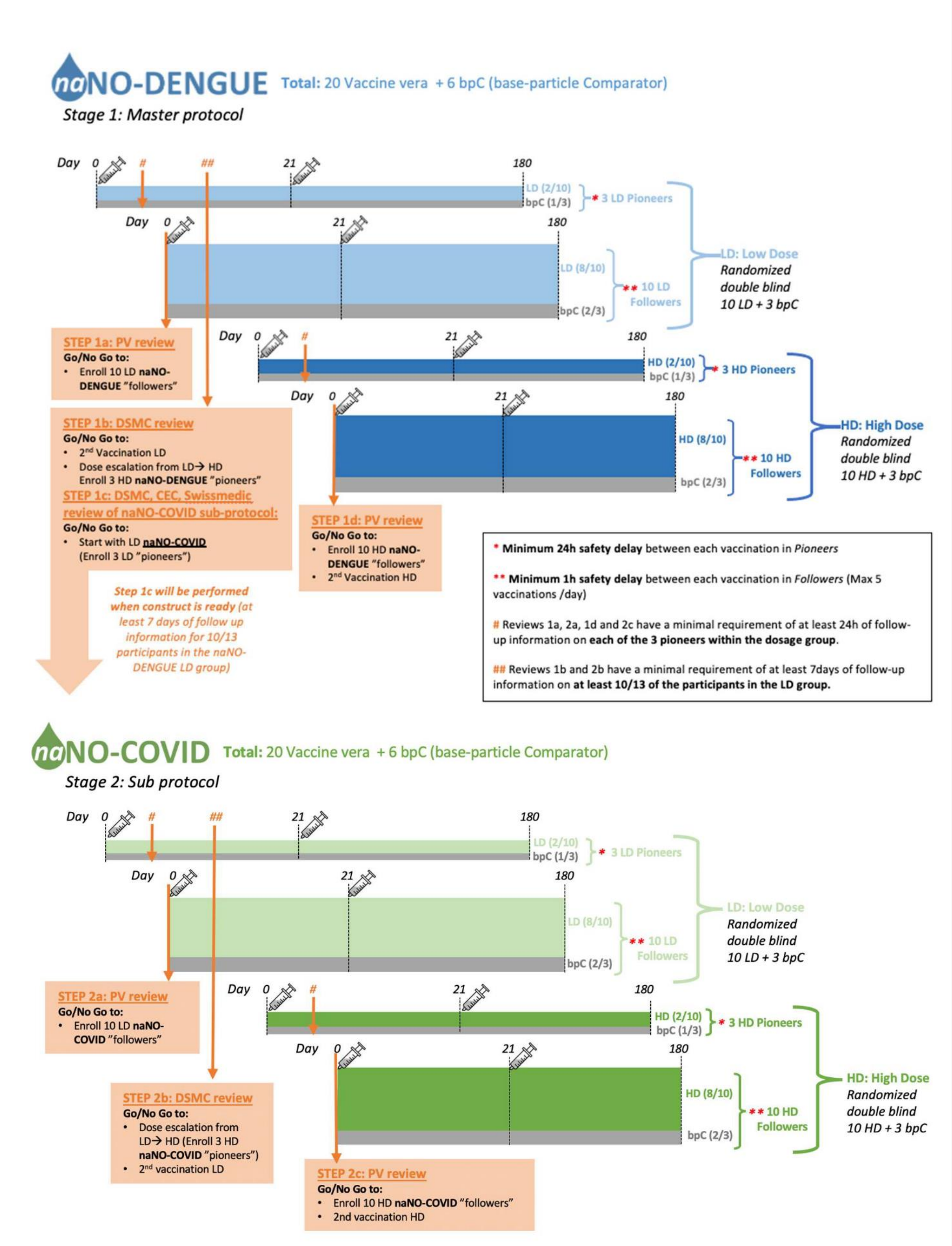

Figure 2. Overview of adaptive trial naNO-DENGUE (master protocol) and naNO-COVID (subprotocol submitted after interim safety analyses)

1. STUDY ADMINISTRATIVE STRUCTURE

1.1 Sponsor

1.1.1 Sponsor's main contact person

Name Emergex Vaccines Holding Limited

Address Contact person: Dr Athanasios Papadopoulos
4 & 5 Dunmore Court Wootton Road, Abingdon,
Oxfordshire, England, OX13 6BH

Telephone +44 1235 527 589 M: +44 7444 857 354

Email ap@emergexvaccines.com

1.1.2 Sponsor's clinical research associate

Name Lorraine Mumtaz

Address 4 & 5 Dunmore Court Wootton Road, Abingdon,
Oxfordshire, England, OX13 6BH

Telephone M: +44 (0)7490 874619

Email lm@emergexvaccines.com

1.1.3 Sponsor's Representative in Switzerland

Name Prof Blaise Genton

Address **Unisanté**, Département Formation, recherche et innovation
Policlinique de médecine tropicale, voyages et vaccinations
Rue du Bugnon 44, 1011 Lausanne, Suisse

Telephone T: +41 21 314 49 32 secr. M:+41 79 556 58 68

Email blaise.genton@unisante.ch

1.2 Investigators

1.2.1 Principal investigator

Name Prof Blaise Genton

Address **Unisanté**, Département Formation, recherche et innovation
Policlinique de médecine tropicale, voyages et vaccinations
Rue du Bugnon 44, 1011 Lausanne, Suisse

Telephone T: +41 21 314 49 32 secr. M:+41 79 556 58 68

Email blaise.genton@unisante.ch

Role Principal investigator

1.2.2 Co-Investigator

Name Dr Mary-Anne Hartley

Address **Unisanté**, Digital Global Health

	**Unisanté**, Département Formation, recherche et innovation<br>Policlinique de médecine tropicale, voyages et vaccinations<br>Rue du Bugnon 44, 1011 Lausanne, Suisse
Telephone	+41 78 866 71 60
Email	Mary-anne.hartley@unisante.ch
Role	Co-lead,<br>Assist the PI in all study tasks

1.2.3 Project coordinator

**Name**	Dr Alix Miauton
Address	**Unisanté** Policlinique de médecine tropicale, voyages et vaccinations<br>Rue du Bugnon 44, 1011 Lausanne, Suisse
Telephone	+41 79 556 87 95
Email	Alix.miauton@unisante.ch
Role	Project coordinator and research physician, Assist the PI in all study tasks

1.3 Statistician

**Name**	Dr Mohamed Faouzi
Address	Biopole 2<br>Route Corniche 10, 1010 Lausanne, Suisse
Telephone	+41 21 314 72 87
Email	Mohamed.Faouzi@unisante.ch
Role	Analysis, Interpretation of data, Participation in the clinical study report writing

1.4 Laboratory

**Name**	Prof François Spertini
Address	Immunology and Allergy CHUV<br>Rue du Bugnon 46, 1011 Lausanne, Suisse
Telephone	+41 21 314 07 90
Email	Francois.spertini@chuv.ch
Role	Designs and perform immunological assays
**Name**	Dr Régine Audran
Address	Immunology and Allergy CHUV<br>CLE D2, Chemin des Boveresses 155, 1066 Epalinges Lausanne
Telephone	+41 21 314 08 57
Email	Regine.audran@chuv.ch
Role	Designs and perform immunological assays<br>Analysis and interpretation of data<br>Writing of immunological study report

1.5 Monitoring institution

**Name**	Elisabeth Reus
Address	Medical Department
	Swiss Tropical and Public Health Institute, Socinstrasse 57, 4002 Basel
Telephone	+41 61 284 89 66
Email	Elisabeth.reus@swisstph.ch
Role	Study monitor

1.6 Data Safety Monitoring Committee (DSMC)

Chairperson

Name: Dr Pierre Landry
Affiliation: Cabinet de Médecine générale, Neuchâtel
Competence: Clinician
Phone: +41 32 724 55 33
Email: Pierre.landry@bluewin.ch

DSMC members (clinicians)

Name: Dr Laura Rothuizen
Affiliation: Direction pharmacologie clinique
Competence: Clinician
Phone: +41 21 314 42 57, +41 79 556 75 24
Email: laura.rothuizen@chuv.ch

Name: Prof. Thierry Buclin
Affiliation: Direction pharmacologie clinique
Competence: Clinician
Phone: +41 21 314 42 61, +41 79 556 68 58
Email: thierry.buclin@chuv.ch

DSMC member (statistician)

Name: Prof. Paul Milligan
Affiliation: London School of Tropical and Hygiene
Competence: Epidemiologist
Phone: +44 78 855 551 83
Email: Paul.Milligan@lshtm.ac.uk

1.7 Any other relevant Committee, Person, Organisation, Institution

1.7.1 Pharmacovigilance

Name: Qvigilance Clinical Research Organization
Contact person: Jane Austin
Address: Bevan House, 9-11 Bancroft Court, Hitchin, Hertfordshire, SG5 1LH
Phone: +44 (0) 1462 477955
Email: jane.austin@qvigilance.com

1.7.2 Local Pharmacy

Name: Service de pharmacie, CHUV
Address: Rue du Bugnon 46, 1011 Lausanne
Phone: +41 21 314 01 38
Email: pha.etudes@chuv.ch

1.7.3 Regulatory Affairs and Project Management Support

Name: Clinical Trial Unit (CTU*)* CHUV & University of Lausanne (UNIL)
Contact person: Prof. Dr Marc Froissart
Address: Mont Paisible 14, 1011 Lausanne
Phone: +41 21 314 61 84
Email: marc.froissart@chuv.ch

In addition, all the physical visits will take place (under the responsibility of the principal investigator) in the clinical investigation unit of the CTU CHUV-UNIL (Bugnon 19, 1011 Lausanne).

2. ETHICAL AND REGULATORY ASPECTS

The decision of the competent ethics committee and Swissmedic concerning the conduct of the study will be made in writing to the Principal investigator/Sponsor's legal representative in Switzerland before commencement of this study. The clinical study can only begin once approval from all required authorities has been received. Any additional requirements imposed by the authorities shall be implemented.

2.1 Study Registration

This study will be registered in the Clinicaltrials.gov before the study starts.

In addition, the study is registered in the Swiss National Clinical trial Portal (SNCTP via BASEC) in French.

2.2 Categorisation of study

This is a first-in-human phase I safety trial of a new nano-particle vaccine against a virus in a population in which the disease is not endemic. This trial is thus considered of category C according to ClinO.

2.3 Competent Ethics Committee (CEC)

The principal investigator ensures that approval from an appropriately constituted CEC (i.e. Commission cantonale d'éthique de la recherche sur l'être humain du canton de Vaud, CER-VD) is sought for the clinical study.

Any changes in the research activity will be reported by the principal investigator to the lead CEC as per ClinO Art 29. Notifications of immediate safety and protective measures, serious adverse events and annual safety reports are described under **Section 10**.

Premature study end or interruption of the study will be reported within 15 days by the principal investigator to the CEC. The regular end of the study will be reported to the CEC within 90 days and the final study report will be submitted within one year after the end of the study.

2.4 Competent Authorities (CA)

The principal investigator (also acting as Sponsor legal representative in Switzerland) will obtain approval from the competent authority (i.e., Swissmedic) before the start of the clinical trial.

Any changes in the research activity will be reported by the principal investigator as per ClinO Art 34. Notifications of immediate safety and protective measures, serious adverse events and annual safety reports are described in **Section 10.**

Premature study end or interruption of the study will be reported within 15 days to Swissmedic. The regular end of the study will be reported within 90 days and the final study report will be submitted within one year after the end of the study by the principal investigator.

2.5 Ethical Conduct of the Study

The study will be carried out in accordance with the protocol and with principles enunciated in the current version of the Declaration of Helsinki, the guidelines of Good Clinical Practice (GCP) issued by ICH, the Swiss Law and Swiss regulatory authority's requirements. The CEC and regulatory authorities will receive annual safety and interim reports and be informed about study stop/end in agreement with local requirements.

2.6 Declaration of interest

The investigators in this trial have no conflict of interest to declare.

2.7 Patient Information and Informed Consent

Screening (any day from -60 to -3)

The formal consent of a participant, using the CEC-approved study-specific consent form, must be obtained before the participant is submitted to any study procedure.

The CEC-approved study-specific participant information sheets (PIS) will be made available to the volunteer (either by direct contact or via email with a telephone call for oral explanations) at least 72 hours prior to their screening visit. During this physical or telephonic contact, the volunteer will be fully informed orally of all aspects of the trial, the potential risks and their obligations. The following general principles will be emphasized:

- Participation in the study is entirely voluntary
- Refusal to participate involves no penalty or consequence.
- The volunteer may withdraw from the study at any time
- The volunteer is free to ask questions at any time to allow him or her to understand the purpose of the study and the procedures involved
- The study involves research of an investigational vaccine
- There is no direct benefit from participating
- The volunteer's general practitioner may be contacted to corroborate their medical history or seek additional information
- The volunteer's blood samples taken as part of the study will be stored in liquid nitrogen and samples may be sent outside of Switzerland to collaborating laboratories. These samples will be identified only by code numbers.
- The aims of the study and tests to be carried out will be explained.
- Financial compensation will be provided for participation (800CHF/participant) disbursed in 8 instalments of 100CHF at each physical visit. Additionally, travel costs will be covered from their place of domicile.
- All relevant clinical and laboratory results (serology, lab tests and interpretations of physical exams) will be shared directly with the concerned participants.

The volunteer will be given minimum 72h to make an informed decision about his/her participation in the study. The volunteer should read and consider the statement before signing and dating the informed consent form. The consent form must be signed and dated by the investigator (or their designee) at the same time as the volunteer signs. A copy of the fully signed form is given to the volunteer and the original will be retained as part of the study records.

Enrolment (Day 0)

- Before receiving the vaccine, participants will be reminded of the nature of the study, its purpose, the procedures involved, the expected duration, the potential risks and benefits and any discomfort it may entail.
- Participants will be reminded that the study is entirely voluntary and that they may withdraw from the study at any time.

2.8 Participant privacy and confidentiality

The investigator affirms and upholds the principle of the participant's right to privacy and that they shall comply with applicable privacy laws. Especially, anonymity of the participants shall be guaranteed when presenting the data at scientific meetings or publishing them in scientific journals.

Individual participant medical information obtained as a result of this study is considered confidential and non-anonymised disclosure to third parties is prohibited. Participant confidentiality will be further ensured by utilising participant identification code numbers to correspond to treatment data in the computer files.

For data verification purposes, authorised representatives of the Principal investigator, a competent authority (e.g. Swissmedic), or an ethics committee may require direct access to parts of the medical records relevant to the study, including participants' medical history.

2.9 Early termination of the study

The Principal investigator may terminate the study prematurely according to certain circumstances, for example:

- ethical concerns,
- insufficient participant recruitment,
- when the safety of the participants is doubtful or at risk,
- alterations in accepted clinical practice that make the continuation of a clinical trial unwise,
- early evidence of harm of the experimental intervention

2.10 Protocol amendments

2.10.1 Unforeseen amendments and deviations

2.10.1.1 Substantial and non-substantial amendments

Only implemented after approval of the CEC and Swissmedic respectively.

All non-substantial amendments are communicated to Swissmedic as soon as possible if applicable and to the CEC within the Annual Safety Report (ASR).

2.10.1.2 Protocol deviations

Under emergency circumstances, deviations from the protocol to protect the rights, safety and well-being of human participants may proceed without prior approval of the Sponsor and the CEC/Swissmedic. Such deviations shall be documented and reported to the Sponsor.

2.10.1.3 Safety and protection measures

According to ClinO art. 37, safety and protection measures which must be taken immediately will be notified to CEC and Swissmedic within 7 days.

2.10.2 Prospective amendments

This is an adaptive trial, which involves the later submission of a sub-protocol for a COVID-19 construct (which is prepared on the template of this master protocol, but with details specific for the COVID-19 construct). Given the global health urgency of COVID-19 vaccines, this subprotocol will be submitted to the CER-VD and Swissmedic for review as soon as stability testing and GLP procedures allow.

3. BACKGROUND AND RATIONALE

3.1 Background

**Primary research question.** What is the safety and reactogenicity profile of 2 doses of PepGNP-Dengue nanoparticle vaccine in a small cohort of healthy human volunteers?
**Secondary research questions.** What are the immunogenicity and ex vivo efficacy profiles of 2 doses of PepGNP-Dengue nanoparticle vaccine in a small cohort of healthy human volunteers?

3.1.1 The need for a rapidly scalable, highly targeted vaccine platform

The race to develop a novel vaccine for COVID-19 has revealed the need for rapidly scalable vaccines that have reliable immunological targets (1). Currently, hundreds of vaccines against SARS-CoV2 are in development and generally consist of a cocktail of antigens or live-attenuated pathogens which are injected into humans in the hope that they target the *right cells in the right way.* This untargeted approach requires large volumes of peptides to be produced per recipient which negatively impacts scalability.

Nanoparticle antigen delivery systems have been developed to enrich specific targeting of immune receptors. These carrier systems are designed to facilitate antigen uptake and processing by antigen presenting cells (APCs), as well as to control antigen release and protect them from premature proteolytic degradation. This more targeted response also allows us to reduce the effective antigen dose (to nanomoles) and mimic a replicating infection with zero risk of developing the infectious disease.

A disease for which targeting the *right cell in the right way* is particularly important is Dengue viral disease. Dengue is well known for antibody-dependent enhancement, where poorly formed immune responses predispose the individual to severe disease during a second infection.

3.1.2 The need for a Dengue vaccine

Despite being the most frequent and most rapidly expanding arbovirus in the world with an estimated 3.9 billion people at risk, dengue is still listed as a 'neglected tropical disease' (2). Propagating in urban cycles and increasingly frequent and explosive human epidemics, its incidence has increased 8-fold over the past twenty years amassing a global burden currently estimated at 390 million infections per year (3); and further spread is forecast in the wake of global warming (4).

Dengue typically manifests as a febrile disease characterised by non-specific systemic symptoms such as fever, rash, vomiting, and pain. Approximately 2% of patients may develop severe haemorrhagic complications (severe dengue), which may progress to death in up to 20% of cases if not provided with prompt supportive care (5). The scale of dengue incidence places a significant resource burden on health services in endemic settings (6). In highly endemic areas such as Southeast Asia, dengue accounts for approximately 10% of all febrile episodes (7) and it has overtaken malaria as the leading cause of fever in returning travellers from these regions (8-10). With no specific treatment or vaccine, management of Dengue relies on the Sisyphean task of vector control, and the global failure of this resource-draining approach is reflected in the 400% increase in dengue incidence over the past 15 years despite exponential increase in funding (5).

The WHO lists the need for a safe and efficient Dengue vaccine as an essential pillar of its *Global strategy for Dengue Prevention and Control*, and specify that the ideal vaccine candidate to undertake this task should protect against all four major DENV serotypes, be given as a single dose, have long-term immunity and have no serious adverse effects (2). Currently there are no vaccine candidates that fulfil these fundamental criteria.

(11)

The urgent need for a Dengue vaccine during the COVID-19 pandemic. The WHO has warned that COVID-19 has disrupted containment efforts of its mosquito vector and further outbreaks are expected as the pandemic flares across the subtropics. Further, its overlapping symptoms of non-specific febrile disease is likely to cause diagnostic confusion and misallocation of limited COVID-19 resources (12-14). Several endemic countries have already reported 10-year record highs of dengue infection which shows how COVID magnifies the need for Dengue research.

3.1.3 The need for a targeted CD8+ T cell immunity in Dengue vaccines

Infection generally leads to transient homotypic immunity specific to one of the four major disease-causing viral serotypes (DENV 1-4). Contradictory to their intended protective purpose, these homotypic antibodies have been shown to predispose severe outcomes in subsequent heterotypic infections, known as Antibody Dependent Enhancement (ADE) (5). The potentially harmful role of the antibody memory immune response in

the dengue disease process poses a major problem to traditional antibody-mediated (MHC class II-specific) vaccine approaches, and there is thus a need for vaccines which harness selective T cell priming. Currently, there is no available vaccine which is universally safe or effective (15, 16).

In dengue, various murine models have not only indicated the lasting memory potential of MHC class I immunization, but also that the CD8+ T cells generated are able to prevent ADE (17). Indeed, DENV-specific CD8+ T cells in this model are even able to provide protection against heterotypic DENV infection despite the presence of sub-neutralizing titres of cross-reactive antibodies, which would normally predispose ADE (18). Currently, *Dengvaxia* has been found not to induce any CD8+ T cell responses (19). The heterotypic protection of T cell mediated immunity is becoming increasingly important in light of in vitro evidence suggesting that anti-DENV immunity might promote ADE in other flaviviruses (FV) such as Zika (ZIKV) (and vice versa) (20, 21). This hypothesis of ZIKV/DENV cross-reactivity has since been replicated in a nonhuman primate model but there is still little robust evidence in humans, mostly due to the limitations of the large epidemiological retrospective study design which is typically used to investigate the mechanism (22). While the protective role of the MHC class I response in Dengue is generally considered indisputable, a lack of human/human-like models means that the exact protective correlates remain poorly defined. Indeed, pre-clinical studies for Dengue vaccines are particularly limited due to the lack of immunocompetent animal models able toadequately mimic the human response (5). Thus, there is a need to perform longitudinal studies which analysethe antiviral immune response during acute viral infection in humans (23). To address this deficiency, human DENV-challenge models are being adopted to expedite new vaccine trials in small numbers of volunteers in whom critical immune mechanistic research can also be undertaken (22, 24). However, the vaccines being tested in this pipeline still do not specifically target a protective T cell response.

The present trial assesses the safety of the first vaccine candidate designed specifically to elicit a T cell immune response for prevention of an acute and severe manifestation of a viral infection by Dengue and other flaviviruses. It thus has the potential not only as a multitarget vaccine but also as a model in which to study further protective immune responses in order to refine and expand the efficacy of subsequent vaccine generations. Of specific interest is the potential of this vaccine platform for SARS-CoV2. A COVID-19 candidate is currently being advanced through rigorous pre-clinical testing and will be available for a phase I trial in early 2021.

**NOTE:** This is the master protocol of a prospective 2-stage adaptive trial, which aims to add a SARS-CoV2 candidate in an identical trial design after interim analyses and review of a sub-protocol including updated literature and completed pre-clinical testing

The need for a COVID-19 vaccine: Despite drastic quarantine measures, SARS-CoV-2 and its associated disease called COVID-19 continues to propagate and threatens global healthcare systems by saturating their capacity with its high transmissibility and the particularly protracted length of stay needed by those requiring intensive care. Under this strain, medical complications and death rates are expected to rise dramatically without early preventative pharmaceutical intervention. Indeed, once patients advance to ICU, prognosis is poor. There is thus universal consensus on the need for a globally effective vaccine to prevent transmission, complications and reduce viral shedding.

CD8+ T cell immunity in COVID-19 vaccines: Emerging evidence suggests that there may be an analogous ADE response in COVID-19. Indeed, several groups have found higher titres of anti-nucleocapsid and anti-spike IgM and IgG to be associated with a worse disease outcome (25, 26). Reviewing of the literature to date, researchers at Yale concluded that “ADE should be given full consideration in the safety evaluation of emerging candidate vaccines for SARS-CoV2”. Additionally, Th1 cell responses are seen as particularly important in mounting an effective immune response against SARS-CoV2 COVID-19 (27, 28).

Updated and more detailed information will be provided in the sub-protocol dedicated to the COVID-19 candidate vaccine.

3.2 Previous human and animal experience

3.2.1 Existing Dengue Vaccines

After years of trial and error, a tetravalent, recombinant, chimeric live dengue vaccine based on the yellow fever 17D backbone has been marketed under the name *Dengvaxia*. The vaccine was released for commercialization in 2015 and is currently registered in 20 countries. However, its use has been subsequently restricted by the WHO due to significant safety and efficacy concerns (29), which fulfilled the anticipated fears of baseline serostatus-dependent efficacy and ADE (30). Specifically, the phase III analyses revealed excess risk of severe dengue in baseline seronegative (naïve to Dengue infection) vaccine recipients, compared with baseline seronegative non-vaccinated controls. Further, vaccination in baseline seronegative individuals of all

ages only conferred modest protection from mild to moderate disease despite developing neutralizing antibodies at high rates (31). As long-term protection only was shown to be present in baseline seropositive (non-naïve to Dengue infection) individuals, the WHO recommended restricting vaccinations to individuals found seropositive by pre-vaccination screening (29). However, there is currently no method capable of reliably screening for past dengue exposure (32). Further, even the protection found in this select group is incomplete, with a notable lack of efficacy against DENV2 (33), and thus still no dengue vaccine exists that is able to protect against all serotypes. Two additional chimeric live-attenuated dengue vaccines have since been adopted in phase III trials: (TV003/TV005; NCT02406729, ClinicalTrials.gov), and (NCT02747927, ClinicalTrials.gov) (5). However, as both are antibody-based approaches, there are still considerable safety concerns. Indeed, as such traditional antibody-mediated vaccine approaches risk inducing severe dengue through ADE, research has focused on identifying a cocktail of Dengue epitopes, which would confer an exclusively protective innate immune response.

3.2.2 Existing T Cell-Specific Vaccines

Two of the most effective immunizations known to medicine, smallpox vaccine (*Dryvax*) and yellow fever virus (YFV)-17D vaccine, are based on an MHC class I-specific mechanism. Smallpox is an exemplar for how effective vaccination can result in disease elimination. In the United States, the last reported case occurred in 1978 and (with the exception of exposed laboratory workers and military personnel), smallpox vaccination was discontinued in 1972. While the yellow fever vaccine is similarly effective at preventing infection, reservoirs of sylvatic transmission and insect vectors have thwarted elimination efforts. The vaccine is currently limited to inhabitants and visitors of high-risk areas. Both *Dryvax* and YFV-17D are based on live virus formulations that result in acute infections with viral replication and a subsequent long-term protective immunity based on an MHC class I immune response (34-37). This immunological memory is established by a pool of memory $CD8^+$ T cells (35) that express the IL-7 receptor (CD127) (38) and that persists for several decades (39, 40). These antiviral $CD8^+$ T cell responses were shown to peak around two weeks post immunization with around 12.5% and 40% of peripheral $CD8^+$ T cells displaying an activated phenotype ($CD38^+$, HLA-DR $^+$, Ki-67 $^+$, Bcl-2^{low}) in YFV-17D and *Dryvax*, respectively, with no detectable evidence of bystander activation (41). Phenotypic analysis of vaccinia virus-specific $CD8^+$ T cells revealed that all effector T cells expressed perforin and granzyme B at the peak of the response. The contraction and memory phase of the response was associated with further differentiation of vaccinia virus-specific T cells. Together, the data support a model of human $CD8^+$ T cell differentiation in which naive $CD8^+$ T cells undergo massive expansion in response to antigen andpass through an effector phase prior to gradually differentiating into long-lived memory cells.

3.2.3 Existing nanoparticle-based vaccines

Nanocarriers of various compositions (lipids, proteins, metals or polymers) have achieved some aspects of these goals. The major advantages of inorganic NPs (such as metals) include low production cost, reproducibility and favourable safety profiles (42). So far, inorganic gold nanoparticles (GNP) have shown a particularly promising immunological and risk profile in several experimental viral vaccines tested in a murine model (e.g. influenza (43), HIV (44) and foot and mouth disease (45)). So far, no safety issues have been reported with gold nanoparticles produced by the Midatech technology after oral or intradermal administration in humans or in any animal model. The possible induction of gold allergy after intradermal injection in some T1 diabetes patients wasn't considered prohibitive to continue dosing in those patients by the regulatory authorities in the UK. NP have also already been used in various human vaccine preparations requiring a cytotoxic CD8 T-cell response. In particular, an NP-based melanoma vaccine containing a CpG adjuvant was tested in a phase I/II study hosted at the same site in Lausanne as this proposed trial. The trial showed that the vaccine was well tolerated, and a majority of patients generated appropriate *ex vivo* T-cell responses (46).

3.3 Investigational product (Dengue Vaccine Candidate) and indication

3.3.1 The vaccine candidate: PepGNP-Dengue

In light of the risks posed by the development of disease-enhancing antibodies and live, genetically modified Dengue compositions, Emergex has developed a potentially antibody-independent vaccine candidate. PepGNP-Dengue is composed of synthetic T cell-selective multivalent Dengue (DENV1-4) peptides carried on carbohydrate-passivated gold nanoparticles (GNP). The Dengue peptides were selected from an

expression library of experimentally determined HLA supertype[8] epitopes[9] that have specificity to MHC class I[10] receptors on T cells. This aims to eliminate the risk of infection from vaccine products and minimise the risk of vaccine-induced ADE.

Below we describe these two components 1) the peptides and their selection and 2) the gold nanoparticle delivery system

**NOTE**: The COVID-19 candidate vaccine (PepGNP-COVID-19) currently undergoing pre-clinical studies in preparation for the naNO-COVID subprotocol has been constructed identically.

3.3.2 Peptide Selection

The peptide selection has been manufactured to GMP quality standards and validated in both pre-clinical and GLP toxicology models; and is thus in line with Phase I development standards.

The "ligandome": To produce a cell-mediated Dengue vaccine, it is necessary to first identify the MHC-I binding peptides (ligands) that are expressed within conserved regions of the Dengue viral family. These peptides were identified using an immunoproteomics approach that has already been validated for a range of viral indications including Dengue, Influenza and Hepatitis B (47-52). Briefly, an HLA-typed human cell line is infected *in vitro* with the relevant virus, after which the peptides expressed on the surface of the infected cells are extracted and identified as the MHC-I "ligandome." The resulting library of peptides represents the repertoire of viral ligands that the immune system selects for the generation of an MHC-I mediated response against the virus. The library was then investigated for eligible vaccine candidate epitopes which would be able to prime a cross-reactive immune response against Dengue infection.

Peptide selection strategy: The nine peptides selected from the Dengue ligandome, are prepared as an equimolar mix (i.e. 1:1:1:1:1:1:1:1:1). The concentration indicated for each dose group refers to the total concentration of all 9 peptides taken together with their AuNP carrier molecules.

The peptides are selected according to the following rationale and criteria:

a) Cross-reactivity between strains based on sequence similarity: Peptides from internal proteins were favoured, as they are generally better conserved within the viral family.
b) Cover particular HLA supertypes: a greater selection of HLA types confers greater population coverage. For this vaccine, HLA-A2/A24/A3 and B7 are included which is predicted to provide >95% coverage.
c) Multiple protein/peptide coverage: An optimal T-cell vaccine would promote multi-target pathogen recognition (with epitopes from various protein structures on the virion). Therefore, multiple peptides for each HLA type were selected.
d) Ease of manufacture: In general, the more hydrophobic the peptide, the more complex the synthesis and conjugation with the nanoparticle carrier system. Therefore, more hydrophilic peptides were given preference when possible.
e) Physio-chemical properties: from an efficacy, cost utility and quality control perspective, it is important that the peptides have the following properties: when bound to the carrier-system, they should not aggregate with themselves (i.e. size <6nm) or other peptides in the vaccine, they should also maintain good solubility and have an acceptable peptide: GNP ratio.

3.3.3 Gold nanoparticle delivery system:

Alone, the above-mentioned peptide epitopes would be immunologically weak when administered *in vivo* and would not produce a T-cell response sufficient for an effective clinical vaccine. Free peptides within the body are subjected to proteolytic degradation and are not efficiently delivered to antigen presenting cells (APCs). GNP technology can overcome these issues. By attaching the viral peptides along with specific carbohydrates to a GNP core (circa 1.7 nm), a vaccine construct can be produced that is immunogenic, protective against proteolytic degradation, and able to efficiently deliver the viral peptides to APCs. This will produce a vaccine that, when administered directly to the dermis via an appropriate device, is capable of delivering the immunologically relevant peptides to the specifically targeted location (*i.e.* dermal APCs), in turn inducing a strong T-cell response. Proof of concept utilizing this system has been demonstrated for pathogens with existing ligandome information (please refer to the relevant section in the Investigators Brochure), thereby

[8] **HLA supertype:** HLA groups with largely overlapping peptide binding specificities
[9] **HLA/MHC epitope:** The part of the peptide molecule to which the HLA molecule binds
[10] **MHC class I:** molecules with the function of presenting peptides to cytotoxic T cells

providing confidence for its application to Dengue vaccine production. This is the first time that this platform is being applied for use in Dengue.

3.3.4 Administration

Due to the low dosage required, the vaccine candidates are suited to be delivered by intradermal delivery devices. The use of a microneedle is indicated to reduce the incidence of trauma-associated adverse local events and discomfort to the volunteer as well as to best mimic the immune cell recruitment during a natural infection by a mosquito (53). In the future this could take the form of novel microneedle skin patch technologies, reducing the training requirements of medical professionals and perhaps allowing vaccinations to take place outside central health clinics.

The development of a dry-patch formulation is intended in the next phase of development.

Vaccinations will be administered to eligible volunteers by microneedles intradermally in the deltoid area of the upper arm. The microneedle used is described in **Figure 3** below adapted from Qiu et al (54).

The needle used in this trial is the Nanopass MicronJet600 (https://www.nanopass.com/product/) which has already been used in a Phase 1 trial for a similar GNP-peptide product for diabetes (https://clinicaltrials.gov/ct2/show/NCT02837094?term=02837094&rank=1).

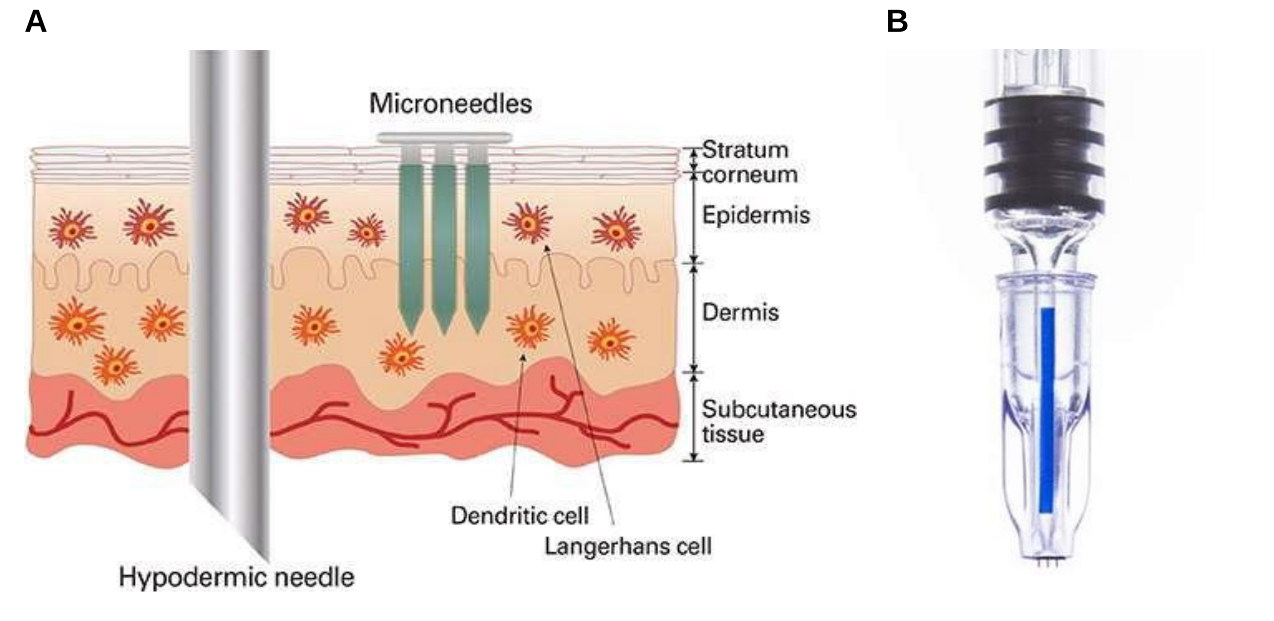

Figure 3. *Microneedle dermal penetrance in comparison to a hypodermic needle (A) and the MicronJet600 needle by Nanopass used in this trial (B)*

3.4 Experimental experience of vaccine candidate

Safety and immunogenicity in vitro and in vivo

The base carrier has been shown to be safe at the proposed clinical doses in repeat dose toxicology studies following subcutaneous delivery in rats (Study 886.341.5682 reference attached to IB) and rabbits (Study 886.342.5683 reference attached to IB).

The candidate vaccine has shown to be safe at the proposed clinical doses and using the same regimen for administration (2 doses using the Nanopass microneedle i.d. delivery system) in GLP toxicology studies in a rabbit model (Study 886.342.5768 reference attached to the Investigator's Brochure). Preclinical efficacy studies include in vitro (utilising human PBMCs) and in vivo (mouse) studies. Finally, high doses of the Dengue vaccine to be used in this trial were tested in a minipig model (a notoriously sensitive animal model in which to study allergic and pseudo-allergic reactions). No evidence of local irritability or hypersensitivity reactions were reported. Further details of these studies are reported in the IB.

3.5 Dose Rationale

This trial evaluates 2 dosages low and high (LD and HD respectively) implemented in a dose escalation

strategy after interim safety analyses

These doses were selected based on meticulous preclinical testing of the vaccine constructs. Research has also shown that peptide vaccine trials' doses so far with 0.1 mg, 0.5 mg, up to 3 mg per peptide are all well-tolerated.

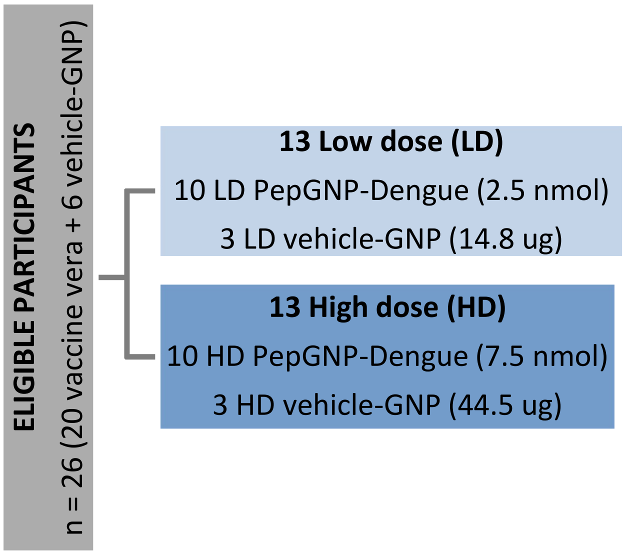

This trial will host a LD of 2.5nmol of PepGNP-Dengue conjugated to 14.8ug of GNP and a HD of 7.5nmol of PepGNP-Dengue conjugated to 44.5ug of GNP.

Overall, it appears that peptide loaded DCs prime the full spectrum of antigen-specific T cells, while selection of low and high avidity responses is a property of non-professional antigen presenting cells or recall (boost) responses. (55)

Therefore, two doses of the vaccine will be given (1+1) in this trial.

3.6 Explanation for choice of comparator

This trial uses a base particle comparator (bpC) arm, as a "vehicle control" with the nano-shell (gold particles without peptide), randomised 10 IMP:3 bpC. A vehicle control was selected in order to allow a better assessment of the attributes of the base particle itself, and better assessment of the specificity of the peptides when added to the base particle.

The bpC will be provided in 2 concentrations that match the GNP dose in the IMP. The LD bpC will be 14.8ug of GNP and the HD will be 44.5ug of GNP.

3.7 Risk-Benefit Analysis

3.7.1 Potential risks and mitigation strategies

a) Antibody-dependent enhancement

The risks of any immune response to a natural dengue infection (or its antigens in a vaccine preparation) is the development of antibody-dependent enhancement (ADE) in subsequent flavivirus infections. However, unlike other failed dengue vaccines, PepGNP-Dengue is specifically designed not to elicit this potentially dangerous antibody response. Rather, the cell-mediated response against dengue anticipated in this trial has been shown to be beneficial to naïve recipients (the targeted population of this trial).

Nevertheless, while the risk of ADE has been observed in the current Dengue vaccine *Dengvaxia*, the WHO still recommends its use, albeit restricted to previously exposed individuals. Indeed, it is important to recognise that the absolute risk of a person developing severe dengue is exceedingly unlikely (particularly in the targeted population of this trial consisting of Dengue-naïve Swiss residents). For instance, a recent systematic review by Halstead et al observes that "the absolute risk of severe dengue in highly endemic areas in children is about 0.4% for secondary infections" and that "about 2-4% of secondary infections lead to severe dengue" (10). Switzerland is not endemic to dengue and it has been observed in the travel clinic which will be running this trial that less than 5% of fevers in travellers returning to Switzerland could be attributed to Dengue.

We have thus aimed to mitigate this risk by using a vaccine candidate that is specifically designed to *not* solicit an antibody immune response. We have also selected a population at especially low risk of secondary Dengue infection.

Finally, this trial will specifically investigate whether participants have developed unexpected anti-DENV antibodies at several time points in the follow-up period. Any antibodies found will be typed and investigated for their neutralising capacity. Participants who develop antibody responses (and are thus seropositive), will qualify for WHO-recommended Dengvaxia vaccination (if the participant plans to reside in an endemic area in the future). This recommendation will be communicated to the participant.

b) Vaccine-induced dengue

This is a synthetic peptide and has no potential to reactivate into a disease-causing pathogen

c) Allergic reactions

Allergic reactions are possible with exposure to any antigen. This includes the antigens of Dengue used in the vaccine preparation as well as to the heavy metal (gold) nanoparticles. Given practical and theoretical evidence, this risk is not estimated to exceed that of currently used vaccines. Indeed, as the vaccine is engineered to present antigen to MHC class I receptors and to *not* produce antibodies, it is theoretically less likely that they will act as a hapten, produce allergy-mediating IgE or engage directly with mast cells. There are also no adjuvants or excipients, which are often the source of vaccine allergies.

As the severity of many allergies is dose-dependent, this trial uses a risk-adverse dose-escalation strategy to minimise the risk of severe allergy and to identify allergic potential. The risk is further mitigated by the SOP of the trial unit, of providing anaphylaxis-specific continuous monitoring for 60-minutes after each vaccine administration.

d) Local adverse events

Most local adverse events of vaccines (at the vaccination site) are caused by traumatic intramuscular needle injury. This trial makes use of microneedles which limit trauma to the dermis and distribute vaccine delivery over a wider area to minimize tissue damage.

e) Interaction with SARS-CoV2

By requiring physical visits in a health facility, the patient may be exposed to SARS-CoV-2 infection. However, the participants will attend the CTU in a location that is not part of the out- or in-patients building, which means that the risk of exposure to SARS-CoV-2 will be almost the same as that in normal life. Also running a trial during the COVID-19 pandemic may influence the results by introducing heterogeneity and febrile disease.

Currently, in Switzerland, there is low transmission of COVID-19. Nevertheless, it must be accepted that COVID-19 is circulating and all fevers and suspected symptoms will be tested for SARS-CoV2.

Additionally, antibodies against SARS-CoV2 will be screened before the vaccination as well as at several points afterwards.

Strict IPC (infection prevention control) measures will be put in place at all meetings as per hospital instructions.

As many as possible interactions will be made telephonic (for instance the first of the 2 screening visits may be replaced by emailing the PIS and discussing queries over the telephone).

3.7.2 Potential benefits

The informed consent will clearly state that there are no immunization benefits anticipated on an individual level. Rather, the benefits are anticipated on a population level, for the advancement of a potential dengue vaccine and novel sustainable vaccine development platform. The participants will provide critical information on the performance and safety of the vaccine, which could not only lead the way to advance the trial to its next phase, but also to evaluate the safety and immunogenicity of a vaccine made on a development platform which could be expanded to include other emerging diseases and cancers. Specifically, we seek to advance to a 2nd stage of this adaptive trial with a COVID-19 vaccine candidate designed on this platform.

The participant will also receive relevant results of their laboratory tests and may find benefit from this information.

3.8 Justification of choice of study population

This study includes healthy men and healthy, non-pregnant, non-breastfeeding women between the ages of 18 and 45 years old who are residing in Switzerland.

Rationale. As a phase 1 trial, healthy participants are essential to study adverse events. As the effect of the vaccine is unknown on the unborn foetus, we exclude women of childbearing potential[11] by the precautionary principle.

We seek to minimize the risk of contracting Dengue, by conducting the trial in a non-endemic region during a

---

[11] An individual who does **not** have childbearing potential is defined as a female who is:

- Pre-menarche or post-menopausal for at least 1 year
- Surgically sterile
- Using an effective method of contraception from at least 4 weeks prior to the first vaccination until at least 10 weeks after the last vaccination (up to day 90). Effective contraception methods are described in the appropriate section.

time when international travel is restricted due to the COVID-19 pandemic.

4. STUDY OBJECTIVES

4.1 Overall objective

The overall objective is to evaluate the safety, tolerability and reactogenicity of two different doses of the investigational Dengue nanovaccine (PepGNP-Dengue) administered to healthy volunteers.

4.2 Primary objective

To evaluate the safety and reactogenicity of two intradermal injections of two different doses of the investigational Dengue peptide T cell inducing vaccine (**PepGNP-Dengue**) administered to healthy volunteers as a

1) candidate vaccine for the prevention of Dengue

2) proof-of-concept for a rapidly scalable modular peptide vaccine platform, which will be followed by a COVID-19 construct after interim analyses.

4.3 Secondary objectives

1) To assess the evidence of a CD8 T-cell mediated immune response as a surrogate of protection against severe dengue disease using a novel peptide set point vaccine in healthy adults.

2) To check the absence of an antibody mediated response

4.4 Exploratory objectives

- To describe any antibody response against the 4 dengue serotypes
- To assess eventual neutralizing or enhancing antibody response if positive for the tests mentioned above.
- To characterize the dengue-specific T (including memory) and B cell responses

4.5 Safety objectives

- Safety is the primary objective of this trial.

5. STUDY OUTCOMES

5.1 Primary outcome measures

The occurrence, nature, time of onset, duration, intensity, seriousness and action taken for any adverse event (AE) reported at any point in the trial (whether it be solicited or unsolicited).

Additionally, the causal relationship to vaccination will also be investigated for any unsolicited event.

Specifically, the:

- Occurrence of <u>solicited local reactogenicity</u> signs and symptoms
  [Time Frame: 7 days following each vaccination]
- Occurrence of <u>solicited systemic reactogenicity</u> signs and symptoms
  [Time Frame: 14 days following each vaccination]
- Occurrence of <u>unsolicited adverse events</u>
  [Time Frame: 6 months following enrolment *i.e. entire trial period*]
- Occurrence of <u>serious adverse events</u> (SAEs)
  [Time Frame: 6 months following enrolment. *i.e. entire trial period*]
- Occurrence of <u>adverse events of special interest</u> (**Section 10.1.1.3**)
  [Time Frame: 6 months following enrolment *i.e. entire trial period*]
- Change from baseline for <u>safety laboratory measures</u> (haematology and biochemistry blood results)
  [Time Frame: 6 months following enrolment *i.e. entire trial period*]

Note: all AEs will be recorded throughout the study according to Swiss ClinO ordinance. The timelines above refer only to outcome measures.

5.2 Secondary outcome measures

Periodic blood sampling will be undertaken according to the visit schedule in **Figure 1** for the following immunogenicity assessments:

5.2.1 Assess cellular immunogenicity of the candidate vaccine (PepGNP-Dengue):

- Proportion of participants with CD8-T cell specific to PepGNP-Dengue
  [Time Frame: 6 months following enrolment]

5.2.1.1 <u>Specific measures to assess evidence of CD8 T cell response:</u>

- Frequency of peptide-specific CD8+ T cells on PBMCs by cytometry *ex vivo,* using staining with dengue specific dextramers, CD3, CD4, CD8 and memory markers (CD45RA, CCR7) at D0, D35 and D180;
- Frequency of peptide-specific CD8+ T cells by cytometry using activation-induced markers (AIM) CD69, CD137 and CD107a upon stimulation with dengue peptides, at D0, D21, D35, D90 and D180.

5.2.2 Assess humoral immunogenicity of the candidate vaccine (PepGNP-Dengue):

- Proportion of participants becoming seropositive (antibodies against Dengue virus) i.e. at potential risk for antibody-mediated disease enhancement
  [Time Frame: 6 months following enrolment]

5.2.2.1 <u>Specific measures to assess vaccine humoral immunogenicity</u>

- **Dengue serology rapid test:** Serology to detect antibodies against the four dengue serotypes by rapid test*;*
- **Anti-DENV2 Ig:** Serology to detect antibodies against natural DENV2 (lysate or inactivated viral particles), by ELISA*;*

5.3 Other outcomes of interest

5.3.1 Exploratory outcome measures

- HLA typing (D0)
  [Time Frame: once]
- Neutralizing or enhancing antibody response (PRNT or cytometry) for any timepoint at which an antibody response is found
  [Time Frame: 6 months following enrolment]
- Cytokine secretion by CD8 in response to vaccine peptides, ICS for IFNγ, TNFα, IL-2, granzyme B.
  [Time Frame: 6 months following enrolment]
- Dengue specific functional assays.
  [Time Frame: 6 months following enrolment]
- In-depth characterisation of the dengue-specific CD8 T cell response.
  [Time Frame: 6 months following enrolment]

6. STUDY DESIGN

6.1 General study design and justification of design

After enrolment at the end of a screening visit, participants will be required to attend a total of eleven further follow-up visits/calls over the 180-day follow-up period as detailed in **Table 2** and summarised in **Figure 1**.

The study visits and procedures will be undertaken by one of the clinical trial team members delegated by the principal investigator. All the physical visits will take place at the CTU CHUV-UNIL, Bugnon 19 in Lausanne. The procedures to be included in each visit are documented in the schedule of attendances. Each visit is assigned a time-point and a window period within which the visit will be conducted. Due to reflexion time, the screening visit will take place over several days. Deviations from the visit windows in completing study visits are discouraged but are permitted at the discretion of the investigator (or designee) in the interest of completingthe study schedule and obtaining participant safety and immunogenicity evaluations. The tolerance limits to these deviations are listed in **Table 2**.

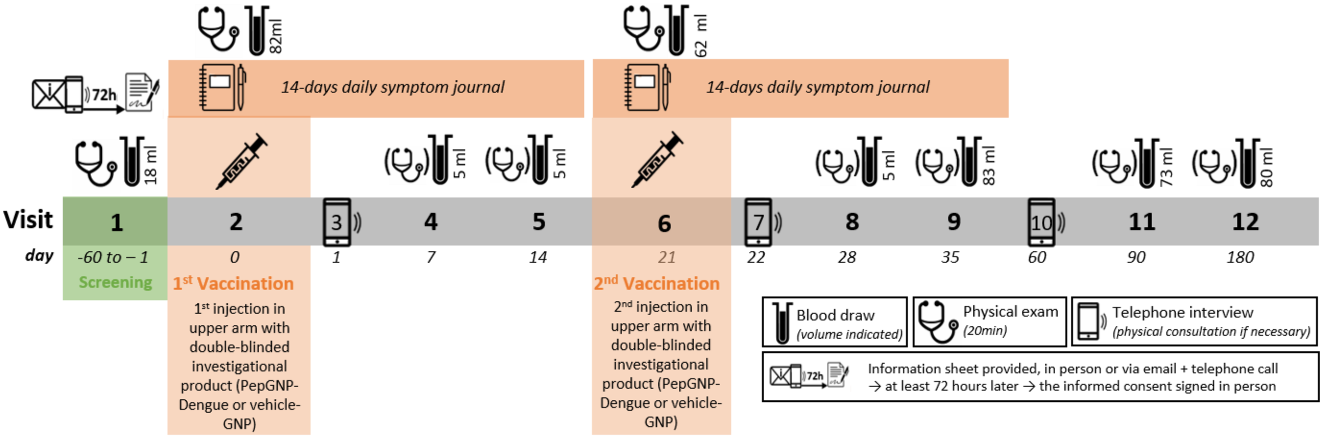

Figure 1: Summarised 180-day schedule of study procedures for a single participant

(copied here for clarity)

6.1.1 Intervention allocation

- **Double-blinded.** Blinded to participants and investigators. Blinding to participants is necessary to ensure that the behaviour (health-seeking, symptom reporting) is not influenced in either arm.
- **Base Particle-controlled.** This trial uses a comparator (Base particle) arm, randomised 3:10. Due to the evolving COVID pandemic, each arm has a temporally matched comparator arm. The naNO-COVID vaccine trial will also have its own comparator arm for this epidemiological reason.
- **Safety analysis.** This trial is powered only to detect major adverse events with a prevalence of over 10%
- **Randomised 3:10 (comparator: intervention).** This is a phase I trial, where exposure must be minimised, and this represents the minimum participant level. The nano-shell vehicle is used for comparator to further minimize risk.

6.1.2 Setting

- **Switzerland.** By running this trial in a region not-endemic to Dengue (Switzerland) during a period of international travel restrictions, we seek to minimize the risk of the participant being exposed to Dengue infection during the trial.
- **Timing concerns for COVID-19.** Regular SARS-CoV-2 serology will be performed throughout the trial. Currently, vaccinations for COVID-19 are expected to be rolled out in early 2021. The exclusion criteria clearly outline the acceptable delays for other vaccinations (4 weeks before the first dose of IMP and 4 weeks after the last dose of IMP). Like in most of the clinical trials (and in particular even for approved and licenced first generation vaccines against SARS-COV-2) during the SARS-COV-2 pandemic, Phase 1 excludes participants with likely current COVID-19. In Phase 2/3, temporary delay criteria defer vaccination of participants with symptoms of potential COVID-19.

6.1.3 Dosages

- **2 dosages with dose-escalation strategy.** To best assess safety, tolerance and (secondarily) efficacy, a

dose-dependency is critical to establish possible causality. If there are adverse events, they are expected to be exacerbated at higher doses, thus by first performing an interim safety analysis on the lower dose, we compartmentalise risk and proceed in an evidence-based, externally validated manner.

- **2 vaccinations per participant.** Booster vaccinations are given at day 21 to reduce the risk of drawing false conclusions of safety or poor-immunogenicity which could occur if the vaccine is poorly administered by chance.

6.1.4 Adaptive design

- This is the master protocol for a 2-stage study investigating the safety of 2 vaccines from a peptide-based T cell inducing vaccine platform for emerging diseases
  - **Stage 1:** naNO-DENGUE
    A Phase-I study of a nanoparticle-based peptide vaccine against Dengue virus (Master protocol)
  - **Stage 2:** naNO-COVID
    A Phase-I study of a nanoparticle-based peptide vaccine against SARS-CoV2 (Sub-protocol, implemented after interim safety analyses)

6.1.5 Timing

- **6 months follow-up.** The duration of the study for each participant is 6 months. This is an adequate follow-up period for many phase I vaccine trials.

6.2 Methods of minimizing bias

6.2.1 Randomisation

A total of 26 eligible participants will be randomized into the following groups:

- Group 1 (n=13): 10 Low Dose (LD) PepGNP-Dengue (2.5 nmol) + 3 Comparator
- Group 2 (n=13): 10 High Dose (HD) PepGNP-Dengue (7.5 nmol) + 3 Comparator

Thus, 20/26 vaccine vera and 6/26 Comparator controls. Allocations of vaccine vera vs Comparator for each group are double-blinded.

The Pioneer group will be randomised individually, while the 10 participants in the follower group will be randomised by block of 5.

Randomization will be performed by the pharmacist.

6.2.2 Blinding procedures

- This trial is double-blinded (blinded to investigators and participants)
- Blinding will be maintained for the duration of the study.
- Bulk concentration of PepGNP-Dengue and Comparator will be supplied to the CHUV by Emergex, where the vaccines are formulated and vialed. Reconstitution and dose dilution will be performed by a pharmacist at the CHUV and transferred to ready-for-use blinded vaccine syringes. Syringes will be labelled with the coded participant ID and certified for release by the pharmacist.
- All allocations will remain coded to all volunteers and investigators. An independent pharmacy team at the CHUV will label the vaccine and Comparator doses with coded participant numbers but will not have access to the identifier list linking the code to the participant identity. All vaccine and Comparator doses will be prepared and labelled away from investigators and stored in identical conditions.
- The appearance of the comparators and doses will be identical. The solutions of both are indistinguishable within the dosage group and thus no shielding of the solution colour is needed.

6.2.3 Other methods of minimizing bias

While the allocation of vaccine vera vs comparator will be double blinded and randomised, the allocation between LD or HD groups and the allocation to pioneer or follower groups will not be double blinded as the schedule reveals the allocation (i.e. the first vaccinated group = LD pioneer). However, the volunteer's position in the schedule will not be shared with the volunteer and they will thus be single blinded to the allocation of dosage (HD or LD) and the allocation to the pioneer or follower group is single blinded to the volunteer.

6.3 Unblinding Procedures (Code break)

- This trial is double-blinded.
- Blinding will be maintained for the duration of the study.
-

6.3.1 Unblinding during the trial

- The PI or Delegate will unblind any participant if deemed medically necessary for clinical management
- In case of a SUSAR, the PI or Delegate must unblind the concerned participant.
- At the request of the DSMC (on a case-by-case basis for SAE and when a holding rule is activated), the DSMC statistician or Delegate will unblind the participant(s) concerned. The decision has to be transcribed within the committee meeting minutes.

6.3.2 Unblinding at the end of the trial

- Allocations will be revealed once the trial has been completed and the database locked.
- Unblinding will also be performed in case of premature termination of the trial.

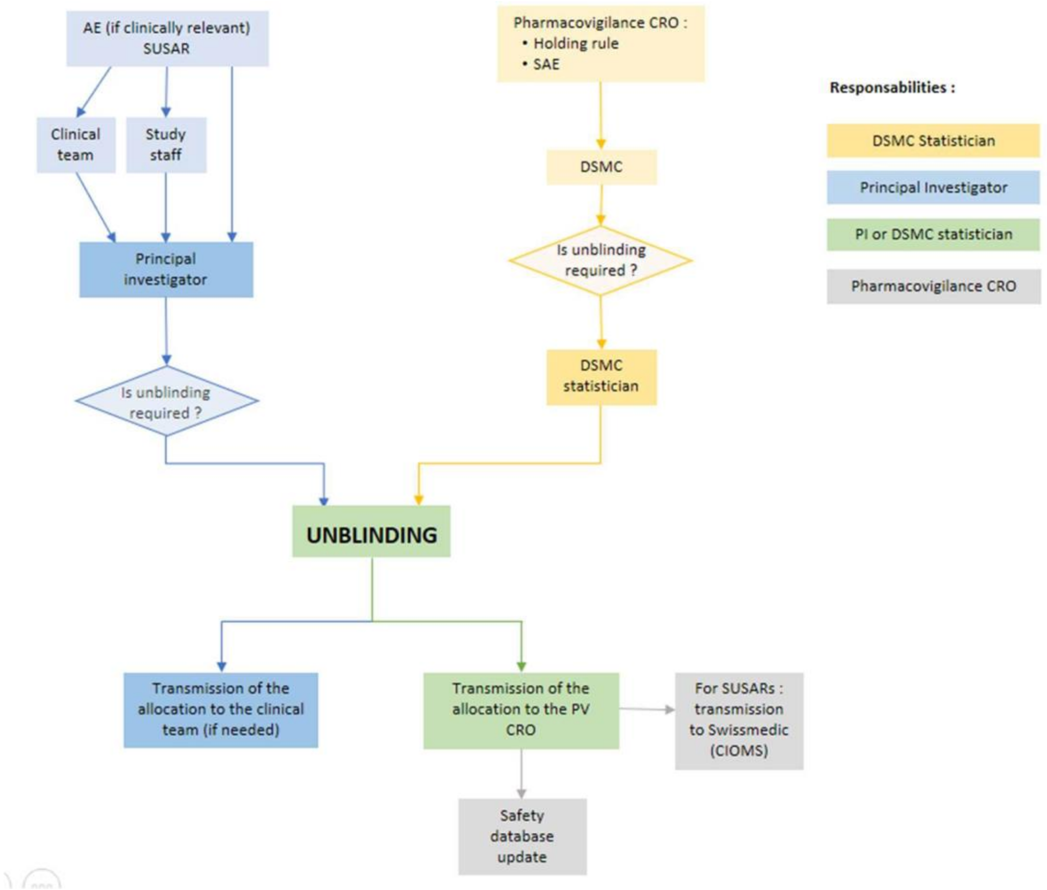

Figure 4: Unblinding reporting

7. STUDY POPULATION

7.1 Eligibility criteria

This study includes healthy men and healthy, non-pregnant, non-breastfeeding women between the ages of 18 and 45 years old who are residing in Switzerland.

**Inclusion criteria:**	An individual must fulfil all of the following criteria in order to be eligible for trial enrolment:<br>1. Aged 18 to 45 years on the day of inclusion<br>2. Participant signed informed consent<br>3. Residing in Switzerland
**Exclusion Criteria**	**An individual fulfilling any of the following criteria is to be excluded from enrolment:**<br>1. Participant is pregnant, lactating, or of childbearing potential[12]<br>2. Participation in the 4 weeks preceding the first trial vaccination or planned participation during the present trial period in another clinical trial investigating a vaccine, drug, medical device, or medical procedure<br>3. Receipt of any vaccine in the 4 weeks preceding the first trial vaccination (excepting influenza vaccination, which may be received up to 2 weeks before first study vaccine) or planned receipt of any vaccine in the 4 weeks following each trial vaccination.<br>4. Previous vaccination against Japanese encephalitis (JE), Yellow Fever (YF), or any dengue virus vaccine (monovalent or tetravalent) at any time in the past with either a trial vaccine or another vaccine (commercial or investigational) based on medical history<br>5. Self-reported or documented history of flavivirus (FV) infection (e.g. DENV, YF, WNV, JE, TBE), confirmed either clinically or serologically<br>6. Receipt of immunoglobulins, blood or blood-derived products in the past 3 months<br>7. Known or suspected congenital or acquired immunodeficiency; or receipt of immunosuppressive therapy[13]<br>8. Self-reported or documented seropositivity for human immunodeficiency virus (HIV), hepatitis B natural infection (HBcAb positive serology), or hepatitis C<br>9. Previous residence for more than 12 months in, or travel in the last 30 days to FV-endemic regions (excluding TBE and WNV)<br>10. At high risk for dengue infection during the trial[14]<br>11. Known systemic hypersensitivity to any of the vaccine components (e.g. gold), or history of a life-threatening reaction to vaccines, or to a vaccine containing any of the same substances<br>12. Current alcohol abuse or drug addiction (reported or suspected)<br>13. Chronic illness that, in the opinion of the investigator, is at a stage where it might interfere with trial conduct or completion<br>14. Thrombocytopenia or any coagulation disorder<br>15. Identified as an Investigator or employee of the Investigator or study centre with direct involvement in the proposed study, or identified as an immediate family member (i.e. parent, spouse, natural or adopted child) of the Investigator or employee with direct involvement in the proposed study (i.e. in the employment of the Tropivac clinic or DFRI unit at Unisanté).<br>16. Refusal to be informed in the event that relevant results concerning the participant's health are revealed

[12] An individual who does **not** have childbearing potential is defined as a female who is:

- Pre-menarche or post-menopausal for at least 1 year
- Surgically sterile
- Using an effective method of contraception from at least 4 weeks prior to the first vaccination until at least 10 weeks after the last vaccination (up to day 90). Effective contraception methods are described in the appropriate section.

[13] Such as anti-cancer chemotherapy or radiation therapy, within the preceding 6 months; or long-term systemic corticosteroid therapy (prednisone or equivalent for more than 2 consecutive weeks within the past 3 months)

[14] Participants travelling to countries/regions with high dengue endemic or epidemic activity

**Exclusion Criteria at the Time of Vaccination**<br>*(where delayed administration is possible)*	The following events constitute contraindications to the administration of the investigational product on the day of planned vaccination.<br>The participant must be followed until resolution of the event as with any medical event and may be considered for vaccination at a later date (maximum 14 days later) or withdrawn at the discretion of the Investigator. Delays due to these events do notconstitute a protocol deviation.<br>• Temperature of >37.5°C at the time of vaccination<br>• Acute disease[15] at the time of vaccination<br>• If there is a clinical/epidemiological suspicion of COVID-19 (according to the clinician's judgement), the participant will be asked to first take a PCR/rapid test for SARS-CoV2, and the vaccination will be delayed until the result comes back negative and the symptoms have resolved.

7.1.1 Effective Contraception for Female Volunteers

Volunteers or their partners of reproductive capacity [16] are required to use an effective form of contraception during the course of the study. As this is a Phase I study, there is no information on the potential teratogenicity of the product. As there are no live viral components used in the composition or manufacture of the vaccine and thus no risk of sexual transmission of replicating viral products (as described for other flaviviruses such as ZIKV (56)) males will not be asked to practice continuous contraception.

Acceptable forms of contraception include:

- Established use of oral, injected or implanted hormonal methods of contraception (for more than 1 month)
- Placement of an intrauterine device (IUD) or intrauterine system (IUS) (for more than 1 month)
- Barrier methods of contraception (condom or occlusive cap with spermicide)
- Male (partner) or female (self) sterilization.
- Abstinence

7.1.2 Influenza and SARS-COV-2 vaccinations

Volunteers are required not to receive any vaccination during the 28-day period preceding or following either PepGNP-Dengue injection (while for influenza, this period is reduced to 14 days preceding the IMP and 28 days afterward). Both SARS-COV-2 and influenza vaccination is encouraged prior to the study (i.e.

- >14-days before enrolment for Influenza and
- >28 days before enrolment for COVID-19, if the participant is eligible for the vaccine according to local recommendations.

Since the study may take place during the influenza and SARS-COV-2 vaccination period, an influenza vaccination will be proposed to the medical and paramedical staff during the 2-month pre-vaccinationscreening period (at least 14 days prior to experimental vaccination) or >28 days after second injection. Any such vaccination will be recorded in the participant's study record.

7.2 Recruitment and Screening

7.2.1 Recruitment

This study includes healthy adults aged 18-45 years residing in Switzerland.

Volunteers will be recruited from the local population around the study site in Switzerland, such as staff from the CHUV, Unisanté (who do not work in the department of the principal investigator), students, or any other interested individuals reached via public advertisements or information sessions. Such information sessions

[15] "Acute disease" is defined as the presence of a moderate or severe illness with or without fever according to investigator judgment. All vaccines can be administered to persons with a minor illness such as diarrhoea, mild upper respiratory infection with or without low-grade febrile illness, i.e. axillary temperature of ≤37.5°C.

[16] An individual who does **not** have childbearing potential is defined as a female who is:

- Pre-menarche or post-menopausal for at least 1 year
- Surgically sterile
- Using an effective method of contraception from at least 4 weeks prior to the first vaccination until at least 10 weeks after the last vaccination (until day 90). Effective contraception methods are described in the appropriate section.

will be conducted via visio-conferences and/or physically at CHUV, Unisanté, UNIL, other health professionals, schools and directed at all staff and students in the context of general information about Dengue. A powerpoint presentation containing exclusively wordings taken from the CEC approved information sheet & informed consent document will be used as support during these information sessions.

A dedicated webpage will be created on the internet website of Unisanté, presenting exclusively the information detailed in the CEC approved information sheet & informed consent document and advertisement document (same wording).

Advertisements - formally approved by the competent ethics committee will be disseminated via academic and hospital contacts as well as being posted in the following places:

- Intranet and social media platforms of the hospital and surrounding academic institutions (CHUV, Unisanté, UNIL, EPFL, nursing schools, Swiss TPH etc.)
- Intranet at the CHUV, Unisanté, HUG and Swiss TPH
- On stalls or stands at exhibitions or fairs in the surrounding hospitals (CHUV, Unisanté) and academic institutions (UNIL, EPFL, nursing schools, Swiss TPH)
- Via presentations (e.g. presentations at lectures or invited seminars)
- By email distribution to lists including staff, students and individuals who have already expressed an interest in taking part in any clinical trial at the CHUV or HUG

If the above do not provide sufficient participants, advertisements will be extended to

- Newspapers or other literature for circulation via press release
- Specific internet networks or agencies such as TrialReach

7.2.2 Screening (Day -60 to -1)

- **Informed consent.** All individuals showing interest in participating in the study will be provided with an information sheet (either by direct contact or electronically + phone call) and be given 72 hours to decide whether they would like further information from study investigator and/or sign the consent if willing.
- Informed consent will be taken before any screening procedure, as described previously in section 2.7. If consent is obtained, the screening procedures indicated in the schedule of attendances will be undertaken (**Table 2**).
- **Screening.** Consenting participants will undergo eligibility testing comprising of a structured interview on their medical history as well as a targeted physical exam. Blood and urine samples will also be collected for clinical laboratory tests which include general assessments of organ function[17] as well as screening for a panel of infectious diseases [18]. Specifically, all eligible participants will be screened for Dengue exposure [19].
    - **Physical exam.** Pulse, blood pressure and temperature will be checked along with a general physical exam of the skin and targeted cardio-respiratory exam (auscultation).
    - **Reacting to abnormal clinical or laboratory findings**. From the medical history, physical examination, urinalysis or blood tests at screening will be assessed as detailed in **Appendix B**. If an abnormal test result is deemed clinically significant it may be repeated to ensure it is not a single occurrence. If an abnormal finding is deemed to be clinically significant, the volunteer will be informed and appropriate medical care arranged with the permission of the volunteer. Decisions to exclude the volunteer from enrolling in the trial or to withdraw a volunteer from the trial will be at the discretion of the investigator and within the inclusion and exclusion criteria.
    - **Pregnancy screening.** All eligible female participants will undergo a human chorionic gonadotropin β-subunit (βhCG) urine pregnancy test before receiving any vaccination (preformed again on the days of both proposed vaccinations to ensure the pregnancy status has not changed in the interim).

[17] Such as full blood count [FBC], alanine aminotransferase [ALT], aspartate aminotransferase [AST], total bilirubin, serum creatinine Urine samples will be tested for the presence of protein, blood, and glucose
[18] Such as human immunodeficiency virus (HIV), hepatitis B virus (HBV), hepatitis C virus (HCV)
[19] Specifically, serologies will be performed for Dengue and an oral history will be taken for Zika virus (ZIKV), Japanese encephalitis (JE) and yellow fever (YF) with cross checking in vaccine cards and travel history.A dengue rapid diagnostic test (combo test, IgG/IgM+NS1) will be performed before and after immunization to assess the usefulness of this exam for evaluating antibody generation in the context of future dengue vaccine trials.

7.3 Assignment to study groups

7.3.1 Randomisation

A total of 26 eligible participants will be randomized into 1 of the following 4 groups:

- 10 Low Dose (LD) PepGNP-Dengue (2.5 nmol)
- 3 Comparator (for LD)
- 10 High Dose (HD) PepGNP-Dengue (7.5 nmol)
- 3 Comparator (for HD)

Each group will then be randomized into two sub-groups:

- A smaller “**pioneer**” group with 3 participants who will trial the first exposure to the dose
  - 2 vaccine vera
  - 1 Comparator
- A larger “**follower**” group comprising the remaining 10 participants who will only be enrolled 5 days after the pioneer group if no holding rules were activated during this observation period.
  - 8 vaccine vera
  - 2 Comparator

Thus, 20/26 vaccine vera and 6/26 Comparator controls.

The second “booster” vaccination will be identical to the first that the patient previously received.

Allocations of vaccine vera vs Comparator for each group are double-blinded.

While the allocation of vaccine vera vs comparator will be double blinded and randomised, the allocation between LD or HD groups and the allocation to pioneer or follower groups will not be double blinded as the schedule reveals the allocation. Only the investigator would know this schedule, and so allocation of dosage (HD or LD) or allocation to the pioneer or follower group is single blinded to the volunteer.

Enrolment will follow a dose escalation strategy (LD--> HD) conditional to a Go/No Go DSMC as depicted in **Figure 2**

A blinded randomisation list will be prepared and a maintained by the pharmacy to assign groups and participants numbers at the clinical site. Thus, randomisation will be performed by the pharmacist.

Randomisation will only be performed once eligibility has been confirmed on Day 0.

The Pioneer group will be randomised individually, while the 10 participants in the follower group will be randomised by block of 5.

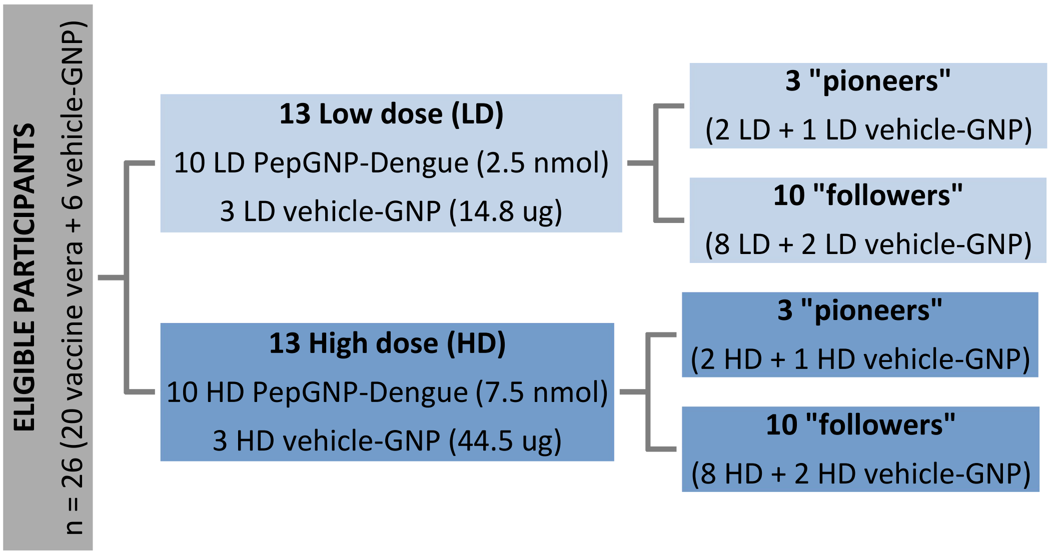

7.4 Criteria for withdrawal / discontinuation of participants

In accordance with the principles of the current revision of the Declaration of Helsinki and any other applicable regulations, a volunteer has the right to withdraw from the study at any time and for any reason and is not obliged to give his or her reasons for doing so. The investigator may withdraw the volunteer at any time in the interests of the volunteer's health and well-being. In addition, the volunteer may withdraw/be withdrawn for any of the following reasons:

- Administrative decision by the Investigator.
- Volunteer non-compliance with study requirements.
- Any event which requires discontinuation of the study involvement or results in inability to continue to comply with study procedures.
- Recommendation by the Pharmacovigilance CRO or DSMC The

withdrawal category will be recorded in the case report form (CRF).

Any volunteer who fails to attend for three or more physical follow-up visits during the study without a clear rationale will be deemed to have withdrawn from the study.

7.4.1 Replacement of withdrawn participants

If a volunteer withdraws or is withdrawn before day 28 (or withdraws after day 28 but did not provide sufficient data or biological material to complete the analyses of the primary objective of the safety analysis) he/she will be replaced if possible, within the specified time frame (appropriate timing, vaccine dose available etc.).

In the event that the participant withdraws without providing sufficient data, after further recruitment is no longer possible, they will not be replaced and the statistical power to identify safety events will be adjusted in subsequent reports and publications.

8. STUDY INTERVENTION

8.1 Identity of Investigational Medicinal Products

8.1.1 Experimental intervention (PepGNP-Dengue)

The nine peptides selected from the Dengue ligandome, are prepared as a quasi equimolar mix (i.e. almost 1:1:1:1:1:1:1:1:1). The concentration indicated for each dose group refers to the total concentration of all 9 peptides taken together with their GNP carrier molecules.

Form Sterile aqueous, buffered solutions of PepGNP-Dengue vaccine

Composition Each Low Dose (LD) 50 µl dose of peptide vaccine contains:

- 2.5 nmol of active substance (peptide)
- 14.8ug gold base particle
- Water For Injection (WFI)

Each High Dose (HD) 50 µl of peptide vaccine contains:

- 7.5 nmol of active substance (peptide)
- 44.5ug gold base particle
- Water For Injection (WFI)

8.1.2 Control intervention (Base particle Comparator, bpC)

Form The nano-shell vehicle is used for comparator

Composition Each Low Dose (LD) 50 µl of bpC contains:

- 14.8ug gold base particle
- Water For Injection (WFI)

Each High Dose (HD) 50 µl of bpC contains:

- 44.5ug gold base particle
- Water For Injection (WFI)

8.1.3 Packaging, Labelling and Supply

Vaccines will be diluted, labelled and dispensed to clinical team by the central Pharmacy at CHUV on the day of vaccination. This process entails reconstituting the freeze-dried powder with Water for Injection (WFI) with a volume stipulated in the IB. Once diluted, the vaccine/comparator is ready for drawing up in the syringe for administration.

The investigator or study nurse will collect the diluted product and draw up the required quantity for administration at the bedside.

Maintaining blinding during preparation: To maintain blinding and data protection, the pharmacist will not have access to the list linking the code to the volunteer identity. After reconstitution, there is no perceptible difference between prepared vials of vaccine vera and comparator and the volumes to be drawn into the syringe are also identical. The investigator will not be informed of the allocation.

8.1.4 Storage conditions

The vaccine and Base GNP will be stored in their freeze-dried form in a secure freezer at -20°C (temperature controlled) at the central pharmacy of CHUV.

Once reconstituted, the final product should be kept at room temperature.

8.2 Administration of interventions

The vaccine and Base GNP are to be injected using microneedles for intradermal injection into the deltoid region of the arm. The vaccine should not be administered intravascularly, subcutaneously or intramuscularly.

- Vaccinations will take place at the CTU CHUV-FBM in Lausanne where advanced life support drugs, resuscitation equipment and trained site staff will be immediately available for the possible

management of anaphylaxis.
- When choosing an arm for the injection, clinicians should consider whether there is a local injury, skin issue or significant tattoo that precludes administering the injection or will interfere with evaluating the arm after injection.
- If possible, the vaccines on day 0 and day 21 will be performed on different arms.

8.2.1 Experimental intervention (PepGNP-Dengue)

Two injections of 50 µl of PepGNP-Dengue vaccine or comparator will be administered on days 0 and 21 using the Nanopass (described in **section 3.3.4**) microneedles for intradermal injection.

Briefly, the site will be cleaned with 70% alcohol and allowed to dry. A study nurse or investigator specifically trained in the use of the Nanopass needle will then inject the product intradermally. The development of a vaccinal weal will be noted along with any evidence of leakage.

An occlusive bandage will them be placed over the weal and the volunteer will be instructed to not touch the site for 1 hour, during which time they will be under medical surveillance.

At the end of the surveillance, the injection site will be inspected, and any particularities will be noted (erythema etc).

8.2.2 Control intervention (bpC)

As a double blind study, this will be identical to section 8.2.1.

8.3 Dose modifications

Not applicable. If dose modifications are recommended by the DSMC, their use will be included in an amended version of the protocol subject to approval by the CEC and Swissmedic before implementation.

8.4 Compliance with study intervention

All doses in this vaccine study will be administered by the investigational team and recorded in the eCRF. The study medication will be at no time in the possession of the volunteer and compliance to dosage will not, therefore, be an issue.

Non-compliance is defined as missing >50% of physical exams or daily diary entries. These participants will still be considered in the ITT analysis.

Any volunteer who fails to attend the booster vaccination or has other significant protocol deviations, will be considered in the intention-to-treat analyses where appropriate.

8.5 Data Collection and Follow-up for withdrawn participants

If a volunteer withdraws from the study, blood samples collected before their withdrawal from the trial will be used/stored unless the volunteer specifically requests otherwise. All safety and immunogenicity data collected from a volunteer that withdraws after vaccination, or is withdrawn by the investigator, will be used in the per-protocol analysis (if time-point is before withdrawal), or in the intention-to-treat analysis (if time-point is after withdrawal). The blinded allocation of the withdrawn participant will only be revealed at the end of the trial on an opt-in basis.

The participant has the possibility to render their data anonymous.

8.6 Trial specific preventive measures

8.6.1 Medications

- No specific restrictions on medications or treatments are imposed, however, all medications taken during the trial will be logged at each contact in the eCRF.
- In case of fever or pain, paracetamol is recommended (preferred over NSAID) if the patient feels such an intervention is required.

8.6.2 Contraception

The method of contraception for female participants is at the choice of the participant and no specific methods

are contraindicated or favoured.

8.6.3 Vaccines

Vaccinations are part of the exclusion criteria and are not permitted during the trial unless a medical necessity. Any such vaccination will be recorded in the participant's study record.

8.7 Concomitant Interventions (treatments)

- No specific restrictions on routine/concomitant medications or treatments are imposed, however, all medications taken during the trial will be logged at each contact in the eCRF.
- Also, significant deviations in routine/concomitant medication will also be logged.

8.8 Vaccine Accountability

The supply, storage, distribution, return and potential destruction of the vaccines will be undertaken by CHUV pharmacy using GLP SOPs (including recording reception date, batch, expiration date, quantity received, shipment, date of dispensation, participant ID, quantity dispensed, quarantine and destruction or return of unused vials

8.9 Return or Destruction of Study Drug

Unused vaccine and base particle comparator will be destroyed according to GLP by CHUV pharmacy.

9. STUDY ASSESSMENTS

9.1 Table of study procedures and assessments

See next page

9.2 Assessments of outcomes

Please see section 5.

Table 3: Schedule of study procedures

Visits	1 Screening	2 Vaccination 1	3	4	5	6 Vaccination 2	7	8	9	10	11	12
Timeline (days)	Any time from -60 to -1	0	1	7	14	21	22	28	35	60	90	180
Tolerance (days)		0	±1	±1	±2	±2	±1	±1	±2	±7	±14	±14
**Summary of interventions**												
**Screening and trial procedures**												
**1. Inclusion/Exclusion criteria**	✓	✓ *(confirmation)*				✓						
**2. Informed consent**	✓	✓ *(confirmation)*										
**3. Medical history**	✓	✓ *(confirmation)*				✓				✓	✓	✓
**4. Concomitant medication**	✓	✓ *(confirmation)*	✓	✓	✓	✓	✓	✓	✓	✓	✓	✓
**5. Physical exam**	✓	✓ *(confirmation)*	(✓)	(✓)	(✓)	✓	(✓)	(✓)	(✓)	(✓)	(✓)	(✓)
**6. Compensation (CHF)**	0	100	0	100	100	100	0	100	100	0	100	100
**Intervention**												
**7. Study Vaccination**		✓				✓						
**Safety monitoring** *(Safety monitoring is continuous. Opt-in reporting is active on all days throughout study)*												
**8. Provide diary card**		✓ (1st)				✓ (2nd)						
**9. Collect diary card + photo (if any)**					✓ (1st)				✓ (2nd)			
**10. Solicited local reactogenicity**		✓	✓	✓		✓	✓	✓				
**11. Solicited systemic reactogenicity**		✓	✓	✓	✓	✓	✓	✓	✓			
**12. Review AE/SAE/AESI**		✓	✓	✓	✓	✓	✓	✓	✓	✓	✓	✓
**Samples taken for blood safety tests and serology**												
**13. Complete blood count**	2.7 ml			2.7 ml	2.7ml			2.7ml	2.7ml			
**14. Biochemistry**	2.6 ml			2.6ml	2.6ml			2.6ml	2.6ml			
**15. HIV, HBV, HCV**	4.9 ml											
**16. Dengue rapid tests** [a]	✓ POCT				✓				✓		2.7 ml	2.7 ml
**17. SARS-CoV2 test (PCR/rapid test)**		✓				✓						
**18. SARS-CoV2 serology rapid test**	✓ (POCT)				✓				✓		✓	✓
**19. βHCG, urine test**	✓ (urine)	✓ *(pre-vaccine)*				✓ *(pre-vaccine)*						
**20. Contraception**	✓	✓	✓	✓	✓	✓	✓	✓	✓	✓	✓	
**21. Urinary protein, blood, glucose**	✓ (urine)											
**22. Anti-nuclear antibodies** [b]	7.5 ml										7.5ml	

**Samples taken for secondary immunogenicity studies**												
**23. EDTA- K blood (HLA typing)**		4.9ml										
**24. PBMC & plasma**		67.5ml *(pre-vaccine)*				52.5ml *(pre-vaccine)*			67.5ml		52.5 ml	67.5 ml
**25. Serum (Antibody tests)**		9.8 ml *(pre-vaccine)*				9.8 ml *(pre-vaccine)*			9.8ml		9.8ml	9.8 ml
**Secondary Immunogenicity Outcomes**												
**26. Anti-denv2 IgG response**		✓				✓			✓		✓	✓
**27. CD8 cell response** [c, d]		✓				✓			✓		✓	✓
**Exploratory Immunogenicity Outcomes**												
**28. Ig functional assay** [e]		(✓)				(✓)			(✓)		(✓)	(✓)
**29. T and B cell responses assessment** [f]		(✓)				(✓)			(✓)		(✓)	(✓)
**TOTALS**												
**Summary of interventions**												
**Daily vol (ml)**	17.7	82.2	0	5.3	5.3	62.3	0	5.3	82.6	0	72.5	80
**Max cumulative vol (ml)**	17.7	99.9	99.9	105.2	110.5	172.8	172.8	178.1	260.7	260.7	333.2	413.2
**Compensation cumulative (CHF)**	0	100	100	200	300	400	400	500	600	600	700	800

Legend for Table 2

[a] Detection of IgG and IgM against the 4 DENV serotypes at D0 as an exclusion criterion. Those performed on D35, D90 and D180 are for immune response assessment. The development of DENV antibodies during the study period or in response to the study vaccination is not considered an adverse event and does not affect the participant's study schedule.

[b] As a surrogate for autoimmune reactions. A 4 fold increase from baseline along with a positive history of clinical signs/symptoms in line with autoimmunity, will be investigated as an adverse event.

[c] Frequency of peptide-specific CD8+ T cells on PBMCs by cytometry *ex vivo,* using staining with dengue specific dextramers, CD3, CD4, CD8 and memory markers (CD45RA, CCR7) at D0, D35 and D180;

[d] Frequency of peptide-specific CD8+ T cells by cytometry using activation-induced markers (AIM) CD69, CD137 and CD107a upon stimulation with dengue peptides, at D0, D21, D35, D90 and D180.

[e] Serum Ig functional assay, neutralization and enhancement, performed if positive DENV rapid test and/or anti-denv2 ELISA.

[f] Additional exploratory analyses of dengue-specific cellular responses at various time-points: Additional characterization of the vaccine induced response, cytokine secretion, dengue-specific cell functional assays.

	Physical consultation at study site
	Blood draw
	Vaccination
	Telephonic consultation

✓ Performed

(✓) Performed and adjusted according to the results obtained in 26 and 27.

+**photo (if any)** In the first 7 days post-vaccination participant will be asked to take a photo of the injection site in the event of signs/symptoms of local reactogenicity

9.3 Procedures at each visit

9.3.1 Intervention (Day 0 and 21)

- **Pre-vaccine baselines.**
    - Before vaccination, a baseline blood sample will be taken (analyses summarised in **Table 2**)
    - Additionally, medical history and eligibility criteria will be confirmed in case of changes from screening).
    - Volunteers will not be considered enrolled in the study until they have received the first vaccine.
- **Vaccination.** Two intradermal injections of the IMP or control will be administered in the upper arm of each consenting participant. The injections will take place on days 0 and 21 using the Nanopass MicronJet600 (https://www.nanopass.com/product/) microneedle.

  Risk will be minimised by:
    - **Dose escalation strategy (LD--> HD)**
        - Escalation from low to high dose enrolment is conditional to a Go/No Go DSMC review as depicted in **Figure 2**.
    - **Staggering enrolment**
        - Enrolment in either LD or HD will be split into a smaller "pioneer" group of 3 participants (2 vaccine vera) and a larger "follower" group (8 vaccine vera).
        - There will always be at least 24 hours between successive vaccinations in the pioneer group.
        - In the follower groups, up to 5 vaccinations can occur in a single 24h period with a period of at least an hour between each.
    - **Immediate post-vaccinal monitoring**
        - The participant will be monitored for 60 min after vaccination for adverse reactions.
        - Briefly, directly after vaccination, the injection site will be covered with an occlusive dressing and the volunteer will stay in the clinical unit for observation in case of immediate adverse events. Observations will be taken during the 60 minutes following vaccination (+/- 5 minutes). The dressing will then be removed, and the injection site inspected before the volunteer leaves.
        - Advanced Life Support drugs resuscitation equipment and trained site staff will be immediately available for the possible management of anaphylaxis
- **Preparation for follow-up.** A thermometer, tape measure, diary card and participant identification card will be given to each volunteer, with instructions on use, along with the 24-hour emergency telephone number to contact the on-call study physician if needed.

9.3.2 Follow-up (Day 0 to 180)

- **Contacts**
    - **Days 0-14 and Days 21 to 35.** A participant diary will be filled out daily for 14 days after each vaccination from day 0 and day 21 to capture adverse events, body temperature and concomitant medication.
    - In the event of local reactogenicity (pain/swelling/redness at injection site), the participant will be instructed to note all symptoms in the provided diary and take a photo of the injection site on each day of symptoms with the provided ruler placed on adjacent skin in the photographed frame. The patient is instructed to not include any identifying features in this photograph such as his/her face. This photo will be provided to investigators in the following visit and stored within the secured patient file on the NAS-CHUV.
    - **Day 1 and Day 22.** The participant will be called 1 day after each vaccination to screen for adverse events (the presence of adverse events or patient's concerns may trigger a physical examination).
    - **Days 7, 14, 28, 35, 90 and 180.** Six further physical consultations with blood draws will then take place over a 180-day (6-month) follow-up period.
    - **Day 60.** The participant will be called on day 60 to screen for adverse events (the presence of adverse events may trigger a physical examination).

9.3.3 Analyses (during and after the end of recruitment)

Blood and urine samples will be processed at the following sites:

1. At the CHUV haematology, immunology and biochemistry laboratories, using standard procedures (all participants):
   - **Haematology:** Full Blood Count (Hb, Ht, RBC, MCV, platelets, WBC, neutrophils, lymphocytes, monocytes, eosinophils, basophils)
   - **Biochemistry:** Creatinine, AST, ALT, total bilirubin, γ-GT, alkaline phosphatase
   - **Diagnostic serology:** HBsAg, HBcAb, HCV antibodies, HIV antibodies, DENV rapid test
   - **Urinalysis:** Urine will be tested for protein, blood and glucose at screening. For female volunteers only, urine will be tested for beta-human chorionic gonadotrophin (β-HCG) at screening and immediately prior to the vaccinations (Day0 if >7 days from screening and Day 21).
   - **Immunology**: Human Leukocyte Antigen (HLA) typing

2. At the CHUV immunology research laboratory and/or collaborating laboratories:

   Secondary outcomes :
   - Anti-Denv2 antibody (ELISA) (all subjects)
   - CD8 T cell responses by cytometry (dextramer staining), HLA A02, A24, A03 or B07 positive subjects.
   - Bystander activation markers by cytometry, (all subjects).

   Exploratory analysis (all subjects)
   - CD8 T cell responses to vaccine peptides by cytometry (ICS) could be done after cell stimulation and culture with peptides if the analysis by dextramer is not sufficiently sensitive.
   - IFN-γ ELISPOT response to individual vaccine peptides
   - Cytokine secretion in PBMC culture in response to vaccine peptides (Luminex)

   Additional exploratory analyses (in line with the aims of the protocol) of immunological response to dengue vaccine such as functional assays may be performed at the discretion of the investigators and Sponsor on a subset of selected samples.

   Handling possible suspected SARS-CoV-2 infections
   - All fevers or flu like illnesses will be assessed for COVID-19. SARS-CoV-2 antibody titres will also be taken before, during and after the study.
   - This includes any instance of fever ≧38.0°C

   Handling possible suspected natural Dengue infections
   - Planned travel to endemic or epidemic areas during the trial period is a listed exclusion criterion.
   - In the event that a participant does travel to an FV endemic/epidemic area during the trial, he/she will be managed according to the procedures in place locally. If an FV infection is suspected, the recommended tests will be performed locally (in particular, a DENV rapid test). The same will apply, should a participant become ill upon return (in Switzerland or elsewhere) with a suspected FV infection. Expanded immunological testing and serology will also be performed.

3. Sample repository is located at the CHUV immunology research laboratory. Samples will be frozen in liquid nitrogen and kept for 10 years. Collaboration with other specialist laboratories in Switzerland, Europe and outside of Europe for further exploratory laboratory tests related to the trial may occur. This would involve the transfer of serum or plasma and/or PBMC to perform analysis to these laboratories, but the samples would be identified only by a code number. The laboratory tests will be conducted according to the procedures established in the test laboratories.

10. SAFETY

The assessment of safety is the primary outcome of this study.

During the entire duration[20] of the study, all adverse events (AE) and all serious adverse events (SAEs) are collected, fully investigated and documented in participant diaries and case report forms (CRF).

The Sponsor's SOPs provide more detail on safety reporting.

10.1 Definition and assessment of adverse and other safety related events

10.1.1 Definition of Adverse events (AEs)

An AE is any untoward medical occurrence in a volunteer, which may occur during or after administration of an Investigational Medicinal Product (IMP) and does not necessarily have a causal relationship with the intervention. An AE can therefore be any unfavourable and unintended sign (including an abnormal laboratory finding), symptom or disease temporally associated with the study intervention, whether or not considered causally related to the study intervention.

The following guidelines will describe how an AE is recorded/categorised in the study/safety database:

10.1.1.1 ***<u>Solicited AEs (i.e. reactogenicity parameters)</u>***

Solicited AEs will be recorded by the patient on diary cards daily for 14 days after each vaccination (7 days for local AEs and 14 days for systemic AEs). The diary cards will be collected and entered into the eCRF 14 days after each vaccination.

- A consensus list of recognized solicited local and systemic AEs is recommended in the *Guidance for Industry Toxicity Grading Scale for Healthy Adult and Adolescent Volunteers Enrolled in Preventive Vaccine Clinical Trials* document produced by the U.S. Department of Health and Human Services Food and Drug Administration (FDA) in 2007[21]
- These recommendations have been adapted and complemented for this trial and summarised in the tables presented in **Appendix A** (solicited local and systemic clinical measures)

10.1.1.2 ***Unsolicited AEs***

Unsolicited AEs are adverse events that do not appear in the list of solicited AEs.

Unsolicited AEs of all severities will be recorded throughout the study period and will be entered in the study database (eCRF) using the corresponding "Preferred term" listed in the CTCAE [22] . The safety database managed by the Pharmacovigilance (PV) CRO will also capture the MedDRA terminology to ensure interoperability across studies.

10.1.1.3 ***Adverse events of special interest (AESI)***

Adverse events of special interest will be recorded at each contact.
The list of AEs considered of special interest for both Dengue and COVID constructs is adapted from the *D2.3 Priority List of Adverse Events of Special Interest: COVID-19* document, published by the SPEAC (Safety Platform for Emergency vACcines) in 2020 [23]. AESI will be captured using the CTCAE terminology.
AESI include (exhaustive list):

- Generalized convulsion
- Guillain-Barré Syndrome (GBS)
- Acute disseminated encephalomyelitis (ADEM)
- Thrombocytopenia
- Anaphylaxis
- Vasculitides
- AE grade 3

[20] Study duration encompassed the time from when the participant signs the informed consent until the last protocol-specific procedure has been completed, including a safety follow-up period (180 days in total).
[21] Available here: https://www.fda.gov/media/73679/download
[22] Available here: here: https://ctep.cancer.gov/protocolDevelopment/electronic_applications/docs/CTCAE_v5_Quick_Reference_5x7.pdf
[23] Available here: https://brightoncollaboration.us/wp-content/uploads/2020/06/SPEAC_D2.3_V2.0_COVID-19_20200525_public.pdf

10.1.1.4 *Serious Adverse events (SAEs)*

A SAE is an AE that results in any of the following outcomes, whether or not considered related to the study intervention.

- Death
- Life-threatening event (i.e. the volunteer was, in the view of the Investigator, at immediate risk of death from the event that occurred).
    - This does not include an AE that, if it occurred in a more severe form, might have caused death.
- Persistent or significant disability or incapacity (i.e. substantial disruption of one's ability to carry out normal life functions).
- Hospitalisation [24], regardless of length of stay, even if it is a precautionary measure for continued observation, or prolongation of existing hospitalisation.
- An important medical event (that may not cause death, be life threatening, or require hospitalisation) that may, based upon appropriate medical judgement, jeopardise the volunteer and/or require medical or surgical intervention to prevent one of the outcomes listed above.
    - Examples of such medical events include allergic reaction requiring intensive treatment in an emergency room or clinic, blood dyscrasias, or convulsions that do not result in inpatient hospitalisation.
- Congenital anomaly or birth defect [25].

SAEs should be followed until resolution or stabilisation. Participants with ongoing SAEs at study termination (including safety visit) will be further followed up until recovery or until stabilisation of the disease.

Should the investigator become aware of any SAEs experienced outside of the study follow up period that he/she thinks may be related to the IMP these will be reported to the pharmacovigilance support entity (Sponsor/CRO)

10.1.1.5 *Suspected Unexpected Serious Adverse Reactions (SUSARs)*

- The investigator evaluates any SAE that has been reported regarding seriousness, causality and expectedness. If the event is related to the investigational product and is both serious and unexpected, it is classified as a SUSAR.
- All SUSARS will be unblinded in order for their assessment. This assessment will be undertaken by the DSMC so that the allocation is not unnecessarily disclosed to the investigator, nor to the study staff, in order not to make the participant ineligible.

10.1.1.6 *Safety signals*

All suspected new risks and relevant new aspects of known adverse reactions that require safety-related measures.

10.1.1.7 *Safety holding rules*

The safety holding rules which will result in an immediate suspension of the trial (where restart is subject to DSMC review and CEC approval) are listed below:

Table 4: Safety holding rules

**Solicited local or systemic adverse events of grade 3**	> 34% of volunteers[26] within any group or subgroup develop a Grade 3 solicited local or systemic adverse event beginning within 2 days after vaccination (day of vaccination and one subsequent day) and persisting at Grade 3 for >48 hrs.
**Unsolicited adverse events**	> 34% of volunteers[26] within any group or subgroup develop a Grade 3 unsolicited adverse event (including a laboratory adverse event) that is considered possibly, probably or definitely related to either vaccination and persists at Grade 3 for > 48hrs.
**SAE**	> 34% of volunteers[26] within any group or subgroup develop an SAE

[24] **Note:** Hospitalisation (including inpatient or outpatient hospitalisation for an elective procedure) for a pre-existing condition that has not worsened unexpectedly **does not** constitute a serious AE (SAE).
[25] **Note:** rigorous testing, counselling and medical history taking will work to best ensure than pregnancies do not occur within the timeframe of this study.
[26] More than 34% of the 3 members of the pioneer subgroups is ≥ 2/3. More than 34% of the 13 members of the entire dosage group is ≥ 4/13.

**SUSAR**	A SUSAR related to the investigational peptide vaccination occurs
**Death**	Death related to the investigational peptide vaccination occurs

10.1.2 Assessment of AE

10.1.2.1 AE Causality

All AE will be evaluated for causality by the investigational team. In addition, Sponsor (through delegated PV CRO) make a causality assessment of all SAE.

Table 5 below is an adaptation of the ICH E2A guidelines providing more detail on the decision logic for assessing causality. Related AEs are graded as "*Possible*", "*Probable*" or "*Definite*".

Table 5. *Guidelines for assessing the relationship of vaccine administration to an AE.*

**Not related**	**No Relationship**	No temporal relationship to study product ***and***<br>Alternate aetiology (clinical state, environmental or other interventions); ***and***<br>Does not follow known pattern of response to study product
**Related**	**Possible**	Reasonable temporal relationship to study product; ***or***<br>Event not readily produced by clinical state, environmental or other interventions; ***or***<br>Similar pattern of response to that seen with other vaccines
	**Probable**	Reasonable temporal relationship to study product; ***and***<br>Event not readily produced by clinical state, environment, or other interventions ***or***<br>Known pattern of response seen with other vaccines
	**Definite**	Reasonable temporal relationship to study product; ***and***<br>Event not readily produced by clinical state, environment, or other interventions; ***and***<br>Known pattern of response seen with other vaccines

10.1.2.2 AE severity

10.1.2.2.1 Severity of solicited AE

- Severity of solicited AE is graded according to an adapted version of *Guidance for Industry Toxicity Grading Scale for Healthy Adult and Adolescent Volunteers Enrolled in Preventive Vaccine Clinical Trials* document, produced by the U.S. Department of Health and Human Services Food and Drug Administration (FDA) in 2007[27].
- The slight adaptation of these guidelines is that we will not be using "grade 4" severity (as grade 3 is considered a stopping rule).
- These recommendations are summarised in the tables presented in **Appendix A** (Solicited local and systemic AEs).

10.1.2.2.2 Severity of unsolicited AE and AESI

- Severity of Unsolicited AEs is graded according to the CTCAE[28] by the investigator.
- For laboratory measures, a modified version of the *Guidance for Industry Toxicity Grading Scale for Healthy Adult and Adolescent Volunteers Enrolled in Preventive Vaccine Clinical Trials* document and CTCAE will be used for severity grading as summarized in **Appendix B** (Unsolicited abnormal laboratory measures).

---

[27] Available here: https://www.fda.gov/media/73679/download
[28] Available here: here: https://ctep.cancer.gov/protocolDevelopment/electronic_applications/docs/CTCAE_v5_Quick_Reference_5x7.pdf

10.1.3 Follow up and actions taken in response to AEs

10.1.3.1 Follow up

- All non-serious AEs (up to and including grade 2) will be followed until resolution/stabilization or end of the trial (whichever is first). In case there are any non-serious AEs related to the IMP (up to and including grade 2) that are unresolved till the end of the primary clinical trial 6-month follow-up, these will be further followed up until resolution/stabilization up to a maximum of 12 months from the first vaccination.
- AESI, SAEs, SUSARS or any AE that results in a volunteer's withdrawal fromthe study will be followed up until a satisfactory resolution, or until a non-study related causality is assigned (if the volunteer consents to this).

10.1.3.2 Actions taken for individual participants

All clinically relevant AEs will be monitored and handled according to the medical judgement of the clinical investigator.

The volunteer may be followed up more intensively for the purposes of monitoring the evolution of the AE. For example, a telephonic contact may be transformed into a physical visit so that a clinical exam or further blood samples and investigations relevant for the resolution of the AE may be undertaken. Additionally, the volunteer may be referred for specialist assessment.

All actions will be logged and incorporated into the assessment of AE severity.

10.1.3.2.1 Abnormal laboratory measures

All clinically relevant deviations on laboratory measures will be investigated according to the medical judgement of the clinical-investigator.

10.1.3.3 Actions taken on all participants

10.1.3.3.1 Safety Holding Rules

If a holding rule is activated, then all further vaccinations will be held and the allocation of the relevant participants or (as a second step and only if needed) the whole dose group will be unblinded.

10.2 Reporting of serious adverse events (SAE) and other safety related events

Reporting rules are summarised in **Figure 5** below.

All AEs (occurring at any point in the trial) that are observed by the Investigator or reported by the volunteer, whether or not attributed to study medication, will be documented in the eCRF.

Outside of the reporting deadlines specified below (for SAEs, SUSARs, Safety Signals relevant to holding rules), all AEs will be transmitted to the Sponsor through a delegated Pharmacovigilance CRO (PV CRO) at regular intervals (aligned with steps 1a, 1b, 1c, and 1d in **Figure 2** as well as at the end of the study).

10.2.1 Reporting of SAEs

Note that the * indicates "since investigator is first made aware of the safety event in question".

- **PI → Sponsor/PV CRO (24h*):** The Principal investigator (or any delegated site staff) will report immediately and within 24 hours all SAE to the PV CRO. SAEs will be reported by email via the REDCap survey tool). All elements captured within the SAE form featured in the eCRF will be transmitted. This includes free text and auxiliary information required for appropriate assessment of the severity and causality of the SAE. A separate email containing relevant coded source documents (laboratory or radiological exams) will be sent simultaneously.
- **Sponsor/PV CRO → PI (72h*):** The CRO will re-evaluate the SAE and send clarifications/approval to the study team within 48hours by email.
- **Sponsor/PV CRO → DSMC (72h*):** The DSMC will be informed within 72h of the SAE by the Sponsor. The communication will be generated by the PV CRO via email.
- *If fatal:* **PI → CEC (7d*):** SAEs resulting in death are reported to the Ethics Committee via BASEC within 7 days by the principal investigator.
- *If not fatal:* **No further expedited reporting action on initial event.**
  - Periodic follow up on the SAE is then reported to the PV CRO follow-up on new relevant

information/issue modifications via the REDCap survey forms as before.

- The SAE will be followed until a satisfactory resolution/stabilisation occurs.

  - The non-fatal SAEs will be submitted to CEC in the annual safety report.

10.2.2 Reporting of SUSARs

- **PI → Sponsor/PV CRO (24h*):** The Principal investigator or any delegated site staff will report immediately and within 24 hours all SUSARs to the PV CRO. SUSARs will be reported by email via the REDCap survey tool. All elements captured within the SAE form featured in the eCRF will be transmitted. This includes free text and auxiliary information required for appropriate assessment of the severity and causality of the SUSAR. A separate email containing relevant coded source documents (laboratory or radiological exams) will be sent simultaneously.
- **Sponsor/PV CRO → PI (72h*):** The CRO will re-evaluate the SUSAR and send clarifications/approval to the study team within 48hours. Once validated, a CIOMS form will be generated (within 4 days of the event, at the latest). and communicated to PI for reporting to EC
- **Sponsor/PV CRO → DSMC (72h*):** The DSMC will be informed by email within 72h of the SUSAR by the Sponsor.
- *If fatal:*
  - **PI → CEC (7d*):** SUSARs resulting in death are reported to the Ethics Committee via BASEC within 7 days by the principal investigator (using the CIOMS form produced by the PV CRO).
  - **Sponsor/PV CRO → Swissmedic (7d*):** SUSARs resulting in death are reported to Swissmedic via email within 7 days by the Sponsor/PV CRO (using the CIOMS form produced by the PV CRO).
- *If not fatal:*
  - **PI → CEC (15d):** SUSARs not resulting in death are reported to the Ethics Committee via BASEC within 15 days by the principal investigator (using the CIOMS form produced by the PV CRO).
  - **Sponsor/CRO → Swissmedic (15d*):** SUSARs not resulting in death are reported to Swissmedic via email within 15 days by the Sponsor/CRO (using the CIOMS form produced by the PV CRO).
    - Periodic follow up on the SUSAR is then reported to the PV CRO
    - Follow-up information is captured in the REDCap survey forms as before.
    - The SUSAR will be followed until a satisfactory resolution/stabilisation occurs, or until a non-study related causality is assigned (if the volunteer consents to this).

10.2.3 Verification and Reporting of holding rule AEs and AESIs

See **Table 4** for holding rules

- **PI → Sponsor/PV CRO (24h*):** In addition to SAE and SUSAR, the Principal investigator (or any delegated site staff) will immediately report and within 24 hours all AESIs to the PV CRO as delegated by the Sponsor. This reporting will allow the PV CRO to actively screen for activation of one of the safety holding rules. All elements captured within the AE form featured in the eCRF will be transmitted. This includes free text and auxiliary information required for appropriate assessment of the severity and causality of the AE. A separate email containing relevant coded source documents (laboratory or radiological exams) will be sent simultaneously.
- **Sponsor/PV CRO → PI and DSMC (72h*):** The PV CRO will continually monitor the reported holding rule AEs and inform the PI and DSMC as soon as a holding rule is met via email (**Table 4**).
- *If it results in the activation of a holding rule:*
  - **PI → CEC (7d):** the holding rule will be reported to the Ethics Committee via BASEC within 7 days by the principal investigator.
  - **Sponsor/PV CRO → Swissmedic (7d*):** the holding rule will be reported to Swissmedic via email within 7 days by the Sponsor/PV CRO.

10.2.3.1 Reporting activation of holding rules

- In the case a holding rule is met, staff will be immediately informed, and the trial halted.
- If a holding rule is activated, then all further vaccinations will be held and the allocation of the relevant participants or the whole dose group (as a second step and if necessary for evaluation) will be unblinded.

10.2.3.2 Reporting trial termination/suspension

- If the trial is prematurely terminated or suspended, the PI will promptly inform the CEC and Swissmedic of the reason for termination or suspension.

- If the trial is prematurely terminated for any reason, the PI will promptly inform the trial participants and should assure appropriate care and follow-up.

10.2.4 Reporting of safety signals

All suspected new risks that require safety-related measures, i.e. so called safety signals, must be reported to the Sponsor within 24 hours. The Sponsor must report the safety signals within 7 days to the Ethics Committee via BASEC and to Swissmedic.

10.2.5 Reporting and Handling of Pregnancies

Pregnant participants will immediately be withdrawn from the clinical study. Any pregnancy during 180days of the trial will be reported to the Sponsor/PV CRO within 24 hours through dedicated form in REDCap sent by email through survey tool. The course and outcome of the pregnancy will be followed up carefully until outcome, and any abnormal outcome regarding the mother or the child will be documented and reported.

Follow-up for pregnancy and the new-born will be assessed on a case-by case basis by specialist. At a minimum this will entail documenting and reporting routine visits and pregnancy outcomes until the end of the neonatal period.

10.2.6 Assessment, notification and reporting on the use of radiation sources

No radiation exposure is anticipated in this study.

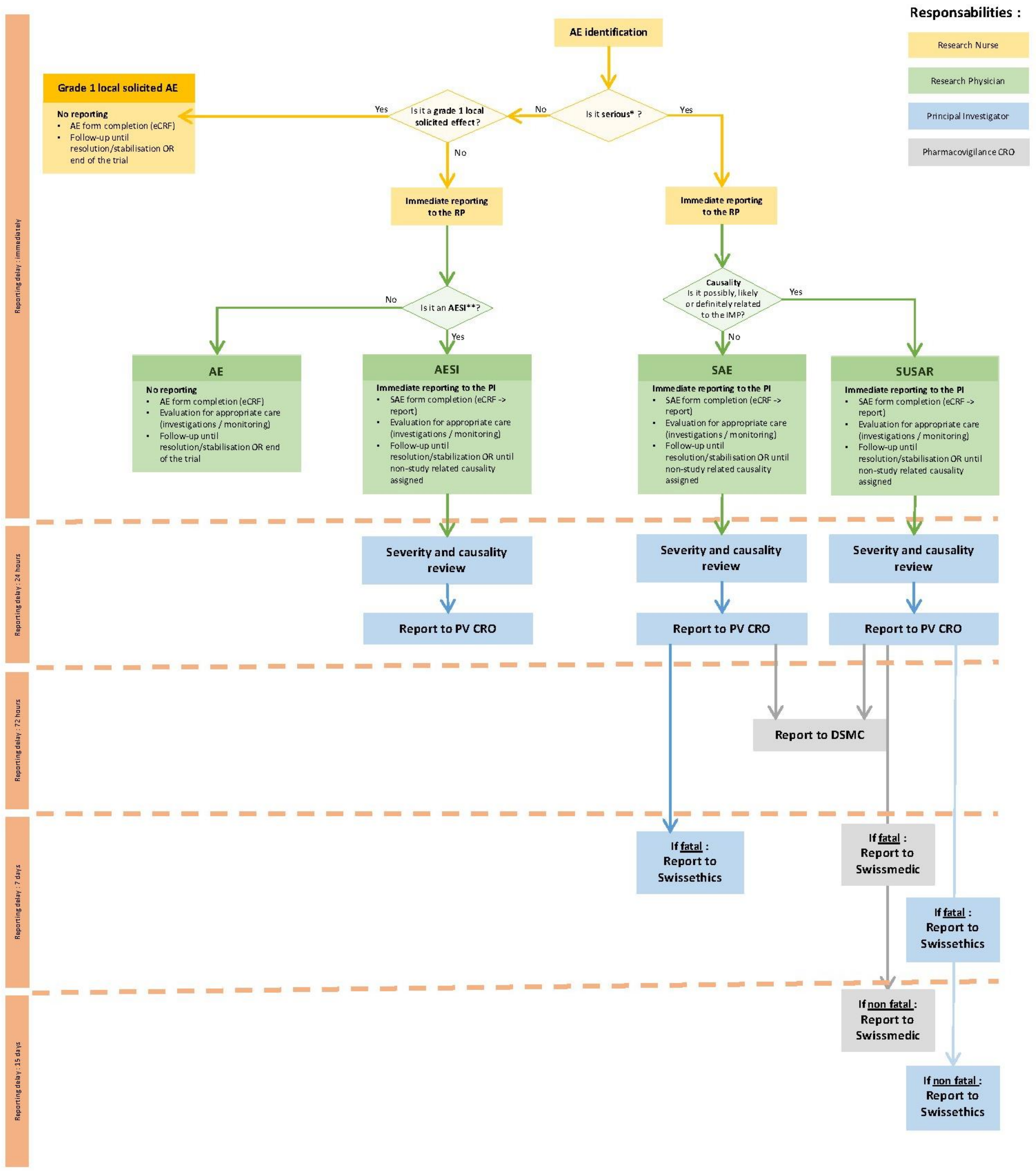

***An AE is serious** when it results in any of the following outcomes, whether or not considered related to the study intervention :

- Death
- Life-threatening event
- Persistent or significant disability or incapacity
- Hospitalisation, regardless of length of stay, even if it is a precautionary measure for continued observation, or prolongation of existing hospitalisation.
- An important medical event that may jeopardise the volunteer and/or require medical or surgical intervention to prevent one of the outcomes listed above.
- Congenital anomaly or birth defect

** **An AESI** is one of the following AE :

- Generalized convulsion
- Guillain-Barré Syndrome (GBS)
- Acute disseminated encephalomyelitis (ADEM)
- Thrombocytopenia
- Anaphylaxis
- Vasculitides
- AE grade 3

Figure 5 Reporting rules for safety events

10.3 Safety reviews

See **Figure 2** for overview.

Safety reviews will consider the following:

- The relationship of eventual AE or SAE to the vaccine.
- The relationship of eventual AE or SAE to the vaccine *dose*, or other possible causes of the event.
- If appropriate, additional screening or laboratory testing for other volunteers to identify those who may develop similar symptoms, and alterations to the current Participant Information Sheet are discussed.
- Eventual new, relevant safety information from ongoing research programs on the various components of the vaccine (i.e. the peptides and the nanoparticle).

Apart from planned analyses, safety data will be continuously monitored. Adverse events will be reviewed by the PI and PV CRO as they arise and according to the above reporting guidelines. The Sponsor will be consulted at each decisional checkpoint.

10.3.1 Data and Safety Monitoring Committee (DSMC) reviews

A Data and Safety Monitoring Committee (DSMC) will be established prior to the trial initiation and will include at least two independent clinicians and one epidemiologist/statistician with relevant expertise in the field or in vaccine development and evaluation of vaccine safety.

Planned interim analysis

An interim analysis by the DSMC will take place using a minimum of 7 days of follow-up data on at least 10/13 participants from the low dose naNO-DENGUE group.

If no holding rules are activated (see below) the DMSC will review the merits for

1) Escalating the dose of naNO-DENGUE from LD→ HD (i.e. enrolling 3 pioneer HD participants)
2) Starting with a second construct against SARS-CoV2 (naNO-COVID), this will be performed in light of the latest emerging scientific evidence which will be summarised in a dedicated subprotocol.
3) Proceeding with the booster vaccine

Unplanned reviews

The DSMC will also review any SAEs or as soon as a holding rule is activated.

The DSMC may also be contacted for advice and independent review by the Investigator or trial Sponsor for any other situation where the Investigator or trial Sponsor feels independent advice or review is important or necessary.

10.3.2 PV CRO Safety Reviews

The safety profile of the investigational product will be assessed on an ongoing basis by the investigators with 2 planned internal safety reviews performed by the PV CRO.

These reviews will serve as Go/No Go checkpoints to continue enrolment within a dose group once a small “pioneer” subset of three individuals have been vaccinated and observed for at least 24h. This serves to minimize risk exposure and initiate early safety responses.

An annual safety report is submitted once a year to the CEC via the Principal Investigator and to Swissmedic via the PV CRO/Sponsor.

11. STATISTICAL METHODS

As this study has no statistical hypothesis test, there is no formal power calculation.

Since this is a first-in-human study with focus on safety, the number of participants exposed to test products needs to be limited. Due to this limitation, only adverse events with high incidence rate will be detected as summarized in the table below.

11.1 Determination of Sample Size

Having ten participants per group (with 20 exposed to the investigational product at either dose), would allow 80% power of detecting an AE with a true incidence of:

- 5% across all exposed participants (LD and HD combined) or
- 20% within a single dose group (LD or HD)

Achievable statistical power (1-β) to observe at least 1 AE at various incidences (λ) within the investigational sample size

True incidence of the AE (λ)	Sample size required to detect a single AE at the statistical power (1-β) listed below		
	**50%**	**80%**	**95%**
**2.5%**	28	64.4	120
**5%**	14	32.2	60
**10%**	7	16.1	30
**20%**	3.5	8.05	15
**30%**	1.75	4.03	7.5

KEY

At least a single ***dose-dependent*** AE would be detectable at the given probability within a single dose group of 10 participants (either LD or HD)
At least a single ***exposure-dependent*** AE would be detectable at the given probability across all dose groups of 20 participants
AE not detectable at this probability within the investigational sample size

Thus, the proposed sample size provides reasonable confidence to assess safety of the novel peptide vaccine and is in line with similar studies previously approved for Phase I evaluation, and at the same time efficiently assessing the evidence of an immunologic response as a surrogate of protection against severe dengue disease.

11.2 Safety analysis

Safety analysis will be carried out for all vaccinated participants, regardless of whether or not they complete the study.

Analysis of the data from this study will be descriptive in nature and no formal statistical hypotheses will be tested. Mean, standard deviation, minimum, maximum (possibly median and quartiles) will be used for continuous variables and number and percentage will be used for categorical variables, unless otherwise specified in the protocol.

Analysis will be applied on per-protocol population, thus only those participants who receive vaccination will be included in the analysis.

The following statistical analyses will be performed on safety data:

1. An external DSMC-led review on a minimum of 24h follow-up data the first 13 participants in the LD group (following the first of two planned vaccinal doses).
2. A final analysis on 6-month follow-up data of all 26 patients spanning all vaccinal doses.

11.3 Statistical Methods for Primary Endpoints:

Event analyses will include the following

- Occurrence of each solicited local adverse event within a 7-day follow-up period (day of each vaccination and 7 subsequent days) after each vaccination.
- Occurrence of each solicited systemic adverse event within a 14-day follow-up period (day of vaccination and 14 subsequent days) after each vaccination.

- Occurrence of unsolicited adverse events within 21 days (day of vaccination and 21 subsequent days) after each vaccination[29].
- Occurrence of serious adverse events over the whole study duration.
- Occurrence of a serious adverse event from the first vaccination to the end of the study.
- Solicited and unsolicited AE data will be collected at each clinic visit. It will be collected from diary cards, clinical review, clinical examination (including observations) and laboratory results. This AE data will be tabulated, and frequency, duration and severity of AEs compared between groups.
- All SAEs will be reported. SAEs, AEs of special interest and withdrawal due to AE(s)/SAE(s) will be described in detail, and relatedness to vaccine will be assessed.
- Haematological and biochemical laboratory values will be presented according to toxicity grading scales and tabulated by group (**Appendix B**).
- The incidence, intensity, and relationship of individual solicited and unsolicited AEs to the vaccine administration will be calculated overall and by group.
- Presentations will include the number and percentage of participants with at least one solicited symptom (local or systemic), at least one local symptom, and at least one systemic symptom, as well as the incidence of each symptom individually.
- The number of participants with at least one report of an unsolicited adverse event reported up to 21 days after the vaccine will also be summarized overall and by group.

11.4 Immunogenicity analysis

For secondary immunogenicity endpoints, descriptive summaries and plots over the time course for both individual volunteer results and groups will be presented. Where appropriate, highly skewed data will be log-transformed and presented as geometric means with confidence intervals. The statistical analysis for immunogenicity will be based on both intention-to-treat and per-protocol principle. Response to vaccination will be evaluated by intra-group comparison of post / pre vaccination results. Assessment of optimal vaccine dose will be evaluated by inter-group comparisons of responses at various time-points.

[29] The occurrence of unsolicited adverse events will in fact be monitored until the end of the study, but only those events occurring within 21 days after the vaccination will be used for the analysis of this primary safety endpoint.

12. QUALITY ASSURANCE AND CONTROL

12.1 Data handling and record keeping / archiving

12.1.1 Case Report Forms

All protocol-required information will be entered in electronic CRFs by the Principal Investigator (or delegated site staff). All source data (such as patient diaries, laboratory results etc.) and volunteer CRFs will be stored securely.

Only the Sponsor, PIs, and delegated site staff will be allowed to access the eCRFs.

The Sponsor will have a limited view of the eCRFs that contain strictly no identifying information.

Once identifying information is no longer required for the basic functioning of the trial (i.e. the requirement to identify patient, address and telephone for follow-up interviews), all eCRFs will be strictly coded (removing the name, initials address and birth date) and only the participant number in combination with year of birth will remain.

This identifying information that is removed from eCRFs at the end of participant follow-up will be transferred to a separate secured file which is independent from the eCRFs. And this will be made available only under request to the PI (if the request pertains to pharmacovigilance monitoring, quality assurance reviews, audits and evaluation of the study safety or a medical concern of the patient, with the patients' consent).

Only the Sponsor representatives, Investigators, the clinical monitor, the CEC and regulatory authorities will have access to the records.

Once the follow-up period of a patient is terminated, all data will be coded: volunteer data will be identified by a unique study number in the eCRF and database.

The study identifier is a unique 3 digit number, automatically generated by REDCap, preceded by the letter "D" (for naNO-DENGUE). A -LD or -HD suffix is then added to specify whether the participant belongs to the low dose or high dose group (e.g.: D-011-LD).

A separate independent confidential file containing identifiable information will be stored in a secured location in accordance with data protection requirements.

12.1.2 Specification of source documents

Source documents are original documents, data, and records from which the volunteer's CRF data are obtained. For this study, medical notes (medical history, vital signs, physical examination, adverse event data, concomitant medication and randomisation number) collected at each visit will be directly entered in electronic CRF. Patient diaries, laboratory records and any additional relevant medical information (e.g. emergency department records and complementary exams in case of serious adverse event), will be collected and stored in paper format. Source data will be secured in Unisanté clinical archives with access granted only to medical professionals participating in the trial.

12.1.3 Record keeping / archiving

The Investigators will maintain appropriate medical and research records for this trial (minimum 10 years), in compliance with ICH E6 GCP and regulatory and institutional requirements for the protection of confidentiality of volunteers. The PI, co-Investigators and clinical research nurses, and medical professionals will have access to records. The Investigators will permit authorized representatives of the Sponsor, monitors, as well as ethicaland regulatory agencies to examine (and when required by applicable law, to copy) clinical records for the purposes of monitoring, quality assurance reviews, audits and evaluation of the study safety and progress.

All the study documents will be kept at site in an Investigator Site File (ISF) while the Trial Master File (TMF) will be kept up to date by Sponsor. All the regulatory submission documents to Swissmedic will be kept in a country specific TMF at site and transferred to Sponsor at the end of the study.

12.2 Data management

The Principal Investigator will have the responsibility for overseeing the receiving, entering, cleaning, querying, analysing and storing all data that accrues from the study by designated persons. Data management will be performed by Unisanté. Throughout, regular data collection and monitoring, clinical data reported on CRFs

and/or relevant serological/biological samples analysis results scheduled in the protocol will be integrated into the electronic Data Capture System (REDcap database). This includes safety data, safety laboratory data and outcome data. Immunological data will be kept in a separate file with the same level of security. The Safety Database is “AB Cube SafetyEasy PV“ which is hosted on AB Cube servers within the EU. AB Cube’s data centres are located at two sites in France, one site holds the primary database servers and the other holds back-up servers for disaster recovery purposes.

For each batch of data, quality control and triggers to computerized logic and/or consistency checks will be systematically applied in order to detect errors or omissions. After integration of all corrections in the complete set of data, the database will be locked and saved before being released for statistical analysis. Each step of this process will be monitored through the implementation of individual passwords and/or regular backups in order to maintain appropriate database access and to guarantee database integrity.

12.2.1 Data Management System

We will be using the REDcap software. The majority of collected data is directly entered into this eCRF and password protected.

REDCap is a secure web application for data collection, with audit trail.

12.3 External monitoring

Monitoring will be performed according to ICH Good Clinical Practice (GCP) by the Swiss Tropical and Public Health Institute under the direct supervision of the Sponsor. Following a Monitoring Plan and written SOPs, the monitors will verify that the clinical trial is conducted and data are generated, documented and reported in compliance with the protocol, GCP and the applicable regulatory requirements. They will perform this role by regular visits to the trial site, direct observation of a participant advancing through the trial pipeline and regularly reviewing reporting documentation.

12.4 Audits and inspections

The study documentation and the source data/documents are accessible to auditors/inspectors (also CEC and CA) and questions are answered during inspections. All involved parties must keep the participant data strictly confidential.

12.5 Confidentiality, Data Protection

Once the follow up period of a patient is terminated, all data will be coded: volunteer data will be identified by a unique study number in the CRF and database. A separate independent confidential file containing identifiable information will be stored in a secured location in accordance with data protection requirements. Only the Sponsor representatives, Investigators, the clinical monitor, the CEC and regulatory authorities will have access to the records.

Photographs taken of local vaccination reactions, rashes or adverse events requiring such documentation (if required, with the volunteer’s written informed consent) will not include the volunteer’s face and will be identified by the date, trial code and participant’s unique coded identifier. Photographs will be stored as confidential records, as above. This material may be shown to other professional staff, used for educational purposes, or included in a scientific publication.

If a study participant is volunteering to communicate with the media, he/she is free to do so on his/her own initiative and responsibility.

The study protocol, documentation, data and all other information generated will be held in strict confidence. No information concerning the study or the data will be released to any unauthorized third party, without prior written approval of the Sponsor. All this information will be given without any names or confidential personal information.

12.6 Storage of biological material and related health data

Biological samples will be kept for 10 years in coded tubes in a secured medical-grade laboratory in CHUV (Department of immunology, CHUV, Lausanne), specific analyses may be outsourced to specialised

collaborating laboratories.

All transfers will be subject to an MTA with conditions specifying the users respect the procedures and ethical standards of this protocol and legal jurisdiction.

According to ClinO art. 45, the Sponsor will keep all data relating to the clinical trial until the expiry date of the last batch of the tested IMP or for at least ten years from the end or the stopping the clinical trial.

13. PUBLICATION AND DISSEMINATION POLICY

The Investigators will be involved in writing and/or reviewing drafts of the manuscripts, abstracts, press releases and any other publications arising from the study. Apart from obvious flaws to the conduct of the study, which may preclude data publication, safety and efficacy data will be published under the supervision and authorization of PI and Sponsor.

Authorship follows the ICJME principles (57)

14. FUNDING AND SUPPORT

The study will be entirely funded by the Sponsor according to the contract signed between Emergex Vaccines Holding Limited and Unisanté.

15. INSURANCE

The potential damages caused to participants will be supported by Newline, represented in Switzerland by Lloyd Switzerland (Seefeldstrasse 7, 8008 Zurich) underwriting contracted by the Sponsor of the study, in accordance with applicable law.

16. REFERENCES

1. Lurie N, Saville M, Hatchett R, Halton J. Developing Covid-19 Vaccines at Pandemic Speed. N Engl J Med. 2020;382(21):1969-73.
2. WHO. Global Strategy for Dengue Prevention and Control: 2012-2020. 2012.
3. WHO. Dengue and Severe Dengue: Fact Sheet 2019 [Available from: www.who.int/news-room/fact-sheets/detail/dengue-and-severe-dengue.
4. Ryan SJ, Carlson CJ, Mordecai EA, Johnson LR. Global expansion and redistribution of Aedes-borne virus transmission risk with climate change. PLoS Negl Trop Dis. 2019;13(3):e0007213.
5. Wilder-Smith A, Ooi EE, Horstick O, Wills B. Dengue. Lancet. 2019;393(10169):350-63.
6. Martelli CM, Siqueira JB, Jr., Parente MP, Zara AL, Oliveira CS, Braga C, et al. Economic Impact of Dengue: Multicenter Study across Four Brazilian Regions. PLoS Negl Trop Dis. 2015;9(9):e0004042.
7. L'Azou M, Moureau A, Sarti E, Nealon J, Zambrano B, Wartel TA, et al. Symptomatic Dengue in Children in 10 Asian and Latin American Countries. N Engl J Med. 2016;374(12):1155-66.
8. Schwartz E, Weld LH, Wilder-Smith A, von Sonnenburg F, Keystone JS, Kain KC, et al. Seasonality, annual trends, and characteristics of dengue among ill returned travelers, 1997-2006. Emerg Infect Dis. 2008;14(7):1081-8.
9. Buss I, Genton B, D'Acremont V. Aetiology of fever in returning travelers and migrants: a systematic review and meta-analysis. J Trav Med 2020 Dec 23;27(8):taaa207. doi: 10.1093/jtm/taaa207
10. Halstead S, Wilder-Smith A. Severe dengue in travellers: pathogenesis, risk and clinical management. J Travel Med. 2019.
11. Rooth I, Bjorkman A. Fever episodes in a holoendemic malaria area of Tanzania: parasitological and clinical findings and diagnostic aspects related to malaria. Trans R Soc Trop Med Hyg. 1992;86(5):479-82.
12. Yan G, Lee CK, Lam LTM, Yan B, Chua YX, Lim AYN, et al. Covert COVID-19 and false-positive dengue serology in Singapore. Lancet Infect Dis. 2020;20(5):536.
13. Smith N. Worst dengue outbreak for seven years in Singapore linked to coronavirus lockdown: The Telegraph; 2020 [Available from: https://www.telegraph.co.uk/global-health/science-and-disease/worst-dengue-outbreak-seven-years-singapore-linked-coronavirus/.
14. WHO. Tailoring malaria interventions in the COVID-19 response: Global Malaria Programme; 2020 [Available from: https://www.who.int/docs/default-source/documents/publications/gmp/tailoring-malaria-interventions-covid-19.pdf.
15. Soucheray S. Sanofi restricts dengue vaccine but downplays antibody enhancement: CIDRAP News; 2017 [Available from: www.cidrap.umn.edu/news-perspective/2017/12/sanofi-restricts-dengue-vaccine-downplays-antibody-enhancement.
16. da Silveira LTC, Tura B, Santos M. Systematic review of dengue vaccine efficacy. BMC Infect Dis. 2019;19(1):750.
17. Zellweger RM, Eddy WE, Tang WW, Miller R, Shresta S. CD8+ T cells prevent antigen-induced antibody-dependent enhancement of dengue disease in mice. J Immunol. 2014;193(8):4117-24.
18. Zellweger RM, Tang WW, Eddy WE, King K, Sanchez MC, Shresta S. CD8+ T Cells Can Mediate Short-Term Protection against Heterotypic Dengue Virus Reinfection in Mice. J Virol. 2015;89(12):6494-505.
19. Guy B, Jackson N. Dengue vaccine: hypotheses to understand CYD-TDV-induced protection. Nat Rev Microbiol. 2016;14(1):45-54.
20. Dejnirattisai W, Supasa P, Wongwiwat W, Rouvinski A, Barba-Spaeth G, Duangchinda T, et al. Dengue virus sero-cross-reactivity drives antibody-dependent enhancement of infection with zika virus. Nat Immunol. 2016;17(9):1102-8.
21. Stettler K, Beltramello M, Espinosa DA, Graham V, Cassotta A, Bianchi S, et al. Specificity, cross-reactivity, and function of antibodies elicited by Zika virus infection. Science. 2016;353(6301):823-6.
22. Slon Campos JL, Mongkolsapaya J, Screaton GR. The immune response against flaviviruses. Nat Immunol. 2018;19(11):1189-98.
23. Appay V, Dunbar PR, Callan M, Klenerman P, Gillespie GM, Papagno L, et al. Memory CD8+ T cells

vary in differentiation phenotype in different persistent virus infections. Nat Med. 2002;8(4):379-85.

24. Kirkpatrick BD, Whitehead SS, Pierce KK, Tibery CM, Grier PL, Hynes NA, et al. The live attenuated dengue vaccine TV003 elicits complete protection against dengue in a human challenge model. Sci Transl Med. 2016;8(330):330ra36.
25. Tan W, Lu Y, Zhang J, Wang J, Dan Y, Tan Z, et al. Viral Kinetics and Antibody Responses in Patients with COVID-19. medRxiv. 2020:2020.03.24.20042382.
26. Jiang H-w, Li Y, Zhang H-n, Wang W, Yang X, Qi H, et al. SARS-CoV-2 proteome microarray for global profiling of COVID-19 specific IgG and IgM responses. Nature Communications. 2020;11(1):3581.
27. Mitch L. T cells found in COVID-19 patients 'bode well' for long-term immunity. Science. 2020.
28. Grifoni A, Weiskopf D, Ramirez SI, Mateus J, Dan JM, Moderbacher CR, et al. Targets of T Cell Responses to SARS-CoV-2 Coronavirus in Humans with COVID-19 Disease and Unexposed Individuals. Cell. 2020; 181,1489-1501.
29. WHO. Dengue vaccine: WHO position paper, September 2018 - Recommendations. Vaccine. 2019;37(35):4848-9.
30. Sridhar S, Luedtke A, Langevin E, Zhu M, Bonaparte M, Machabert T, et al. Effect of Dengue Serostatus on Dengue Vaccine Safety and Efficacy. N Engl J Med. 2018;379(4):327-40.
31. Halstead SB, Russell PK. Protective and immunological behavior of chimeric yellow fever dengue vaccine. Vaccine. 2016;34(14):1643-7.
32. Arien KK, Wilder-Smith A. Dengue vaccine: reliably determining previous exposure. Lancet Glob Health. 2018;6(8):e830-e1.
33. Guy B, Barrere B, Malinowski C, Saville M, Teyssou R, Lang J. From research to phase III: preclinical, industrial and clinical development of the Sanofi Pasteur tetravalent dengue vaccine. Vaccine. 2011;29(42):7229-41.
34. Demkowicz WE, Jr., Ennis FA. Vaccinia virus-specific CD8+ cytotoxic T lymphocytes in humans. J Virol. 1993;67(3):1538-44.
35. Demkowicz WE, Jr., Littaua RA, Wang J, Ennis FA. Human cytotoxic T-cell memory: long-lived responses to vaccinia virus. J Virol. 1996;70(4):2627-31.
36. Littaua RA, Takeda A, Cruz J, Ennis FA. Vaccinia virus-specific human CD4+ cytotoxic T-lymphocyte clones. J Virol. 1992;66(4):2274-80.
37. Mc CK, Downie AW, Bradley WH. The antibody response in man following infection with viruses of the pox group. II. Antibody response following vaccination. J Hyg (Lond). 1958;56(4):466-78.
38. Rock MT, Yoder SM, Talbot TR, Edwards KM, Crowe JE, Jr. Cellular immune responses to diluted and undiluted aventis pasteur smallpox vaccine. J Infect Dis. 2006;194(4):435-43.
39. Crotty S, Felgner P, Davies H, Glidewell J, Villarreal L, Ahmed R. Cutting edge: long-term B cell memory in humans after smallpox vaccination. J Immunol. 2003;171(10):4969-73.
40. Hammarlund E, Lewis MW, Hansen SG, Strelow LI, Nelson JA, Sexton GJ, et al. Duration of antiviral immunity after smallpox vaccination. Nat Med. 2003;9(9):1131-7.
41. Miller JD, van der Most RG, Akondy RS, Glidewell JT, Albott S, Masopust D, et al. Human effector and memory CD8+ T cell responses to smallpox and yellow fever vaccines. Immunity. 2008;28(5):710-22.
42. Pati R, Shevtsov M, Sonawane A. Nanoparticle Vaccines Against Infectious Diseases. Front Immunol. 2018;9:2224.
43. Tao W, Gill HS. M2e-immobilized gold nanoparticles as influenza A vaccine: Role of soluble M2e and longevity of protection. Vaccine. 2015;33(20):2307-15.
44. Xu L, Liu Y, Chen Z, Li W, Liu Y, Wang L, et al. Surface-engineered gold nanorods: promising DNA vaccine adjuvant for HIV-1 treatment. Nano Lett. 2012;12(4):2003-12.
45. Chen YS, Hung YC, Lin WH, Huang GS. Assessment of gold nanoparticles as a size-dependent vaccine carrier for enhancing the antibody response against synthetic foot-and-mouth disease virus peptide. Nanotechnology. 2010;21(19):195101.
46. Speiser DE, Schwarz K, Baumgaertner P, Manolova V, Devevre E, Sterry W, et al. Memory and effector CD8 T-cell responses after nanoparticle vaccination of melanoma patients. J Immunother.2010;33(8):848-58
47. Testa JS, Shetty V, Sinnathamby G, Nickens Z, Hafner J, Kamal S, et al. Conserved MHC class I-

presented dengue virus epitopes identified by immunoproteomics analysis are targets for cross-serotype reactive T-cell response. J Infect Dis. 2012;205(4):647-55.

48. Comber JD, Karabudak A, Huang X, Piazza PA, Marques ET, Philip R. Dengue virus specific dual HLA binding T cell epitopes induce CD8+ T cell responses in seropositive individuals. Hum Vaccin Immunother. 2014;10(12):3531-43.

49. Huang X, Karabudak A, Comber JD, Philip M, Morcol T, Philip R. A novel immunization approach for dengue infection based on conserved T cell epitopes formulated in calcium phosphate nanoparticles. Hum Vaccin Immunother. 2017;13(11):2612-25.

50. Testa JS, Shetty V, Hafner J, Nickens Z, Kamal S, Sinnathamby G, et al. MHC class I-presented T cell epitopes identified by immunoproteomics analysis are targets for a cross reactive influenza-specific T cell response. PLoS One. 2012;7(11):e48484.

51. Comber JD, Karabudak A, Shetty V, Testa JS, Huang X, Philip R. MHC Class I Presented T Cell Epitopes as Potential Antigens for Therapeutic Vaccine against HBV Chronic Infection. Hepat Res Treat. 2014;2014:860562.

52. Testa JS, Philip R. Role of T-cell epitope-based vaccine in prophylactic and therapeutic applications. Future Virol. 2012;7(11):1077-88.

53. Manning JE, Cantaert T. Time to Micromanage the Pathogen-Host-Vector Interface: Considerations for Vaccine Development. Vaccines (Basel). 2019;7(1).

54. Qiu Y, Guo L, Mao P, Gao Y. Dissolving Microneedle Arrays for Intradermal Immunization of Hepatitis B Virus DNA Vaccine. Procedia in Vaccinology. 2015.

55. Leggatt GR. Peptide Dose and/or Structure in Vaccines as a Determinant of T Cell Responses. Vaccines (Basel). 2014;2(3):537-48.

56. Counotte MJ, Kim CR, Wang J, Bernstein K, Deal CD, Broutet NJN, et al. Sexual transmission of Zika virus and other flaviviruses: A living systematic review. PLoS Med. 2018;15(7):e1002611.

57. Zarin DA, Tse T, Williams RJ, Rajakannan T. Update on Trial Registration 11 Years after the ICMJE Policy Was Established. N Engl J Med. 2017;376(4):383-91.

58. FDA. Toxicity Grading Scale for Healthy Adult & Adolescent Volunteers Enrolled in Preventative Vaccine Clinical Trials.

17. APPENDICES

17.1 APPENDIX A: Solicited local and systemic AE

Adapted from (58), available here: https://www.fda.gov/media/73679/download

17.1.1 Solicited Local AE

Local Reaction to Injectable Product	Mild (Grade 1)	Moderate (Grade 2)	Severe (Grade 3)
**Pain**	Does not interfere with activity	Repeated use of nonnarcotic pain reliever > 24 hours or interferes with activity	Any use of narcotic pain reliever or prevents daily activity
**Tenderness**	Mild discomfort to touch	Discomfort with movement	Significant discomfort at rest
**Erythema/Redness ***	2.5 – 5 cm	5.1 – 10 cm	> 10 cm
**Induration/Swelling ****	2.5 – 5 cm and does not interfere with activity	5.1 – 10 cm or interferes with activity	> 10 cm or prevents daily activity

* In addition to grading the measured local reaction at the greatest single diameter, the measurement should be recorded as a continuous variable.
** Induration/Swelling should be evaluated and graded using the functional scale as well as the actual measurement.

17.1.2 Solicited Systemic AE

Systemic (General)	Mild (Grade 1)	Moderate (Grade 2)	Severe (Grade 3)
**Nausea/vomiting**	No interference with activity or 1 – 2 episodes/24 hours	Some interference with activity or > 2 episodes/24 hours	Prevents daily activity, requires outpatient IV hydration
**Diarrhea**	2 – 3 loose stools or < 400 gms/24 hours	4 – 5 stools or 400 – 800 gms/24 hours	6 or more watery stools or > 800gms/24 hours or requires outpatient IV hydration
**Headache**	No interference with activity	Repeated use of nonnarcotic pain reliever > 24 hours or some interference with activity	Significant; any use of narcotic pain reliever or prevents daily activity
**Fatigue**	No interference with activity	Some interference with activity	Significant; prevents daily activity
**Myalgia**	No interference with activity	Some interference with activity	Significant; prevents daily activity
**Fever (°C) by axillary temperature**	37.5 – 37.9	38.0 – 38.4	>38.5

17.2 APPENDIX B: Severity grading for abnormal laboratory measures

Adapted from CTCAE v5.0, available here: https://ctep.cancer.gov/protocolDevelopment/electronic_applications/docs/CTCAE_v5_Quick_Reference_5x7.pdf and from (58), available here: https://www.fda.gov/media/73679/download

LABORATORY TEST	*UNIT*	*REF RANGE*	GRADE 1	GRADE 2	GRADE 3	GRADE 4	GRADE 5
**Hb ↓ (anaemia)**	*g/l*	*♀ 117-157 ♂ 133-177*	<117 – 100	<100 – 80	< 80	Life threatening consequences , urgent intervention indicated	Death
**WBC ↑ (leucocytosis)**	*G/l*	*4.0-10*	10.8 – 15.0	15.1 – 20.0	20.0 – 25.0		
**WBC ↓ (leukopenia)**	*G/l*	*4.0-10*	< 3.5 – 2.5	<2.5 – 1.5	<1.5		
**Neutrophils ↓ (neutropenia)**	*G/l*	*1.8-7.5*	<1.8 – 1.5	<1.5 – 1.0	<1.0		
**Lymphocytes ↓ (lymphopenia)**	*G/l*	*1.5- 4.0*	<1.5 – 0.8	<0.8 – 0.5	<0.5		
**Eosinophils ↑ (eosinophilia)**	*G/l*	*0.05-0.5*	0.5 – 1.5	1.5 – 5.0	>5.0		
**Platelets ↓ (thrombopenia)**	*G/l*	*150-350*	<150 – 125	<125 – 75	<75 OR presence of clinical signs and symptoms of spontaneous bleeding		
**Creatinine ↑**	*umol/l*	*♀ 44-80 ♂ 62-106*	>ULN –1.5 x ULN	>1.5–2.5 ULN	>2.5 x ULN		
**Urea ↑**	*mmol/l*	*♀ 2.9-6.4 ♂ 2.9-7.7*	8.2 – 8.9	9.0 – 11.0	>11.0		
**ALT, AST ↑**	*U/L*	*9-50*	51 – 150	>150 – 250	>250		
**Alkaline phosphate ↑**	*U/L*	*36-120*	121 – 300	>300 – 600	>600		
**GGT ↑**	*U/l*	*6-42*	43 – 130	131 – 210	>210		
**Total Bilirubin ↑**	*umol/l*	*0-21*	22 – 31	32 – 63	>63		
**C reactive protein↑**	*mg/l*	*0-10*	10 – 30	30-80	>80		
**Antinuclear antibodies**	*Titer*	*Patient baseline*	1/1280 or >4 fold increase from baseline	-	-		

Note: the standard analysis panel for “full blood count” includes several measures that are taken are not listed here as part of the analysis of abnormalities (i.e. haematocrit, RBC, MCV, monocytes, basophils)

These additional measures will help indicate the clinical subtype of a given anomaly or provide clinical context, but are not specifically reported.

Urinalysis at screening will be assessed as per the table below:

**URINALYSIS**	
Protein*	1+
Blood**	1+ on two dipstick tests
Glucose	1+

**In the event of the dipstick testing positive for protein with ≥1+ protein urine should be sent for a protein creatinine ratio.*

*** In the event of urine dipstick testing positive for ≥1+ blood with, or without, protein in volunteers a repeat dipstick test will be carried out to confirm haematuria. In female volunteers, a menstrual history will be taken to elicit whether the subject is currently menstruating and if they are, urine dipstick will be repeated after 1 - 2 weeks. If blood and/or proteinuria persist in any volunteer, they will be excluded from the trial, and the appropriate follow-up arranged.*
